# Leaching of Carbon Reinforced Concrete—Part 1: Experimental Investigations

**DOI:** 10.3390/ma13194405

**Published:** 2020-10-02

**Authors:** Lia Weiler, Anya Vollpracht

**Affiliations:** Institute of Building Materials Research, RWTH (Rheinisch-Westfälische Technische Hochschule) Aachen University, 52062 Aachen, Germany; vollpracht@ibac.rwth-aachen.de

**Keywords:** leaching, carbon concrete composite, irrigated construction elements, environmental compatibility, irrigated building materials

## Abstract

The composite material ‘carbon concrete composite (C^3^)’ is currently capturing the building sector as an ‘innovative’ and ‘sustainable’ alternative to steel reinforced concrete. In this work, its environmental compatibility was investigated. The focus of this research was the leaching behavior of C^3^, especially for the application as irrigated façade elements. Laboratory and outdoor exposure tests were run to determine and assess the heavy metal and trace element emissions. In the wake of this work, the validity of laboratory experiments and the transferability to outdoor behavior were investigated. The experimental results show very low releases of environmental harmful substances from carbon concrete composite. Most heavy metal concentrations were in the range of <0.1–8 µg/L, and higher concentrations (up to 32 µg/L) were found for barium, chromium, and copper. Vanadium and zinc concentrations were in the range of 0.1–60 µg/L, boron and nickel concentrations were clearly exceeding 100 µg/L. Most of the high concentrations were found to be a result of the rainfall background concentrations. The material C^3^ is therefore considered to be environmentally friendly. There is no general correlation between laboratory leaching data and outdoor emissions. The results depend on the examined substance and used method. The prediction and evaluation of the leaching of building elements submitted to rain is therefore challenging. This topic is debated in the second part of this publication.

## 1. Introduction

The use of composite building materials offers a wide range of advantages for the building industry as for example new functions, savings in weight and costs, or new design options. This leads to an increasing use of new materials or material combinations in construction with uncertain recycling methods and unknown emission behavior: Physical and chemical bonds between the components, for example, might impede the materials separation for recovery of recyclables after their service life and therefore lower the resource efficiency. Mutual influences or reactions of newly combined substances can change emissions despite an unchanged content level [1,2]. In the context of an existing and rising environmental awareness, the environmental compatibility of building materials is also rising in importance. To reconcile both, it is necessary to consider and avoid a possibly harmful release of substances from the outset.

An important environmental aspect is the leaching of potentially harmful substances from construction elements that are in permanent or temporary contact with water, followed by an entry into the environmental compartments soil, ground- and/or surface waters. Depending on the type of material used, these substances can be inorganic (heavy metals and trace elements) or organic (e.g., unreacted monomers, additives, impurities, degradation products or biocides). The use of construction products without prior testing can therefore lead to environment and health risks. So far, these effects are often not considered in an appropriate way. A detailed study including 100 organic and inorganic substances in the rainwater discharge of Berlin has shown that the discharge of rainwater into rivers can raise concentrations of some contaminants by a factor of ten [3]. The heavy metals chromium, nickel and vanadium are named as construction material, mainly concrete, born emissions [3,4]. This also applies to organic emissions such as nonylphenolic compounds [5]. Zinc and copper from metal roofs, façade coatings and renders were also found to be relevant. It has to be mentioned that a precise allocation of substance leaching to its sources is certainly difficult as concentrations are measured in the runoffs and possible sources are identified by comparison of catchments.

The annual mean concentrations of zinc, copper, lead and sometimes also cadmium in German, French and Austrian rainwater discharge are exceeding the European EQS values (environmental quality standards) [6], and, in case of zinc and copper, the recommendations of the German Federal Environment Agency for the EQS. Irrigated façades and roofs were identified as one source [3,7,8].

Throughout their whole life irrigated construction elements are exposed to an intermittent wet–dry stress. Compared to construction elements that are in permanent contact with water, this can cause a deviating leaching behavior with increased or lower release, depending on the building material and the leached substance. Chromium, for example, seems to be leached in higher amounts in outdoor and irrigation experiments, and barium and selenium are leached to a higher extend in permanent water contact [9]. At the moment there is no broad database available concerning the correlations between standardized laboratory and laboratory irrigation tests; even less is known about correlations with outdoor leaching behavior.

Table 1 shows a summary of previous research projects with laboratory irrigation with a focus on simulated rain events. Studies describing the leaching behavior of biocides from paints and varnishes after EN 16105:2011 [10] or using permanent water contact to replace irrigation are not outlined as they do not meet the desired ‘rain’ criteria.

It is obvious that a broad variety of testing conditions and examined substances was chosen by the researchers. This leads to difficulties in the assessment and aggregation of the available data. It is furthermore revealed (and was also stated by Schoknecht [16] and Nebel [17]) that there are difficulties in adjusting realistic rain intensities, respectively, rain drop sizes at a laboratory scale.

Corresponding conclusions from different studies concerning influencing parameters on leaching of intermittent moistened construction materials pertain to:

- Wet–dry stress: In porous building materials, the drying phases cause a transport of water and dissolved substances to the surface by capillary transport during drying dissolved substances precipitate at the surface. This leads to an increased availability for leaching or wash-off in the following rain [11,13]. The microstructure plays a major role for the transport processes, especially under wet–dry stress. This might lead to changing release patterns over time, as moisture transport additionally fosters transformations of the matrix structure by causing pore changes through, e.g., micro-cracking or shrinkage [11,13,15,18].
- Material composition: Different substances contained in a building material can interact, which can cause bonding but also an increase in the release. The content itself is not decisive for the release of substances [2,11,13,19,20].
- Substance-dependent leaching: Leaching rates depend on physical and chemical properties of the substances and the building material, especially the solubility of the particular substances, which can be pH dependent [9,17,19,21,22]. For intermittent moistened construction elements, the pH of the material, not of the leachate, is pivotal [13,19]. Wet–dry stress, temperature changes and air contact can alter the matrices over time; for example, by carbonation, which leads to decreasing pH values. This influences the long-term leaching [11,19,21,22].

Further parameters and processes such as rain intensities, orientation of test specimen, temperature or leachate composition are relevant too, but were not investigated in detail in laboratory irrigation, whereas several studies, e.g., [13,23,24] are discussing these factors for outdoor exposure. Standardized laboratory leaching tests are not appropriate methods to consider these factors. The disregarding may lead to an over- or underestimation of the real leachate concentrations, depending on the composition of the building material [9,11,13,15,21].

In this paper, investigations concerning the leaching behavior of carbon textile reinforced concrete (C^3^) are described. The use of carbon textiles for concrete reinforcement introduces not only a new material to the system but also allows a fundamental change in the use of the matrix raw materials. In [25], the opportunities of new binder systems and concrete compositions are described. Unlike steel reinforcement, carbon reinforcement does not need an alkaline environment and concrete covering to avoid corrosion [25]. New materials like the organic polymer coating of the reinforcement on the one hand and a potentially lower pH or thin concrete covering on the other hand may cause increasing emissions by leaching.

Due to the novelty of the composite material and the complexity of collecting and assessing long time data concerning, e.g., durability, the development of new matrix compositions is still in progress [25]. This work focuses on C^3^ with already technically approved components. C^3^ as a new composite material combines inorganic and organic components. Heavy metals and trace elements leached from the concrete might interact with organic substances, especially monomers, from the coating of the reinforcement. Especially dissolved organic carbon (DOC) can influence the emission process [2,13]. Biocides for example were found to be retained by the organic reinforcement of fiber cement sheets in Lupsea et al. [1].

To investigate the emission behavior, different test specimens are irrigated using a new laboratory irrigation stand; the released substances are determined and compared to the results of a standardized laboratory test [26], namely the dynamic surface leaching test (DSLT) and outdoor weathering data.

Within the framework of a joint research project, the DSLT data were collected by our partner Verein Deutscher Zementwerke (VDZ) gGmbH and the test specimens were centrally manufactured by the partner Hentschke Bau GmbH.

## 2. Materials and Methods

### 2.1. Materials

Preliminary tests were conducted to identify a matrix-reinforcement combination with comparably high leaching. Based on the results, a fine-grained, ready-mixed concrete for the matrix and a carbon fiber grid for the reinforcement were chosen.

#### 2.1.1. Concrete Composition

**Figure 1:**
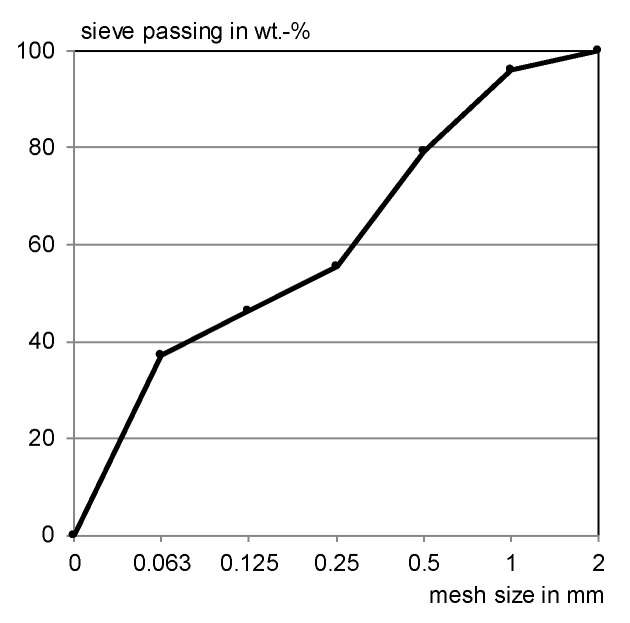
Grading curve of the ready-mixed concrete.

The ready-mixed dry concrete includes fine grained aggregate with a nominal maximum size of 1 mm. Figure 1 shows the sieving analysis results.

The dry mix consists of approximately 57 wt.% sand and 43% binder. The compositions of each fraction determined by X-ray diffraction are given in Table 2.

**Figure 2:**
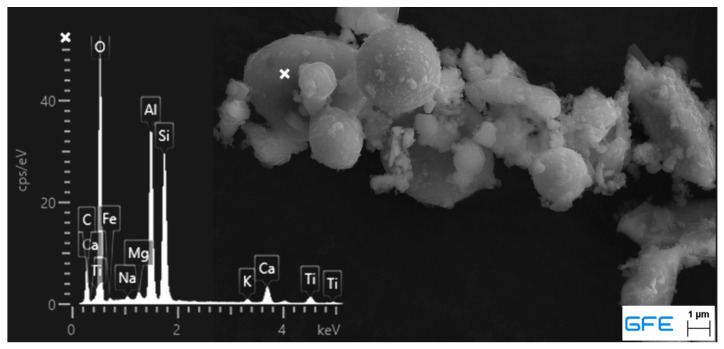
Scan electron microscopy (SEM) picture and corresponding energy dispersive X-ray (EDX) spectrum of ready dry mix <125 µm, fly ash.

**Figure 3:**
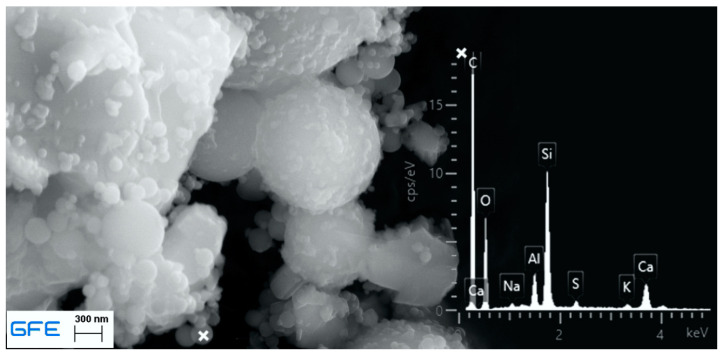
SEM picture and corresponding EDX spectrum of ready dry mix <125 µm, silica fume.

The binder consists of Portland cement clinker and amorphous components. The presence of Mullite and Hematite indicates that the dry mix contains fly ash, which was confirmed by scan electron microscopy (SEM) in combination with energy dispersive X-ray spectroscopy (EDX). Figure 2 shows the typical spherical shape of fly ash particles with the corresponding EDX spectrum for silicon, aluminum and iron. Moreover, silica fume was found to be part of the sample. Figure 3 shows the also spherical-shaped particles in typical agglomerates and with a significant size difference to fly ash. The EDX spectrum shows no iron and less aluminum content, which might also be a result of the excited environment.

Since no granulated blast-furnace slag was found by selective dissolving with HNO_3_/EDTA and only traces were assumed under light microscopy, the whole amorphous share determined by X-ray diffraction is assigned to fly ash and silica fume.

The concrete mixture was obtained with 14 L water per 100 kg dry mix, which corresponds to a water binder ratio of approximately 0.32. During mixing, liquefaction could be observed which most likely traces back to the effect of a superplasticizer in the dry mix.

The fraction <0.125 mm consists mainly of the cementitious binder. This fraction was analyzed for its chemical composition by X-ray fluorescence spectroscopy (XRF), carbon/sulfur analyzer (CSA), and silver nitrate titration (for chloride content). Its trace element and heavy metal contents were analyzed by inductively coupled plasma mass spectrometry (ICP-MS) after aqua regia digestion. The acquired data are presented in Table 3 and Table 4.

Table 4 shows the content of heavy metals and trace elements of both materials determined by ICP-MS after aqua regia digestion. Compared to the average of German cements, the cement used for the ready mix shows no significant deviations in trace elements and heavy metal content. The reinforcement textile contains considerable amounts of copper, nickel and especially chromium in relation to the cement.

#### 2.1.2. Reinforcement

**Figure 4:**
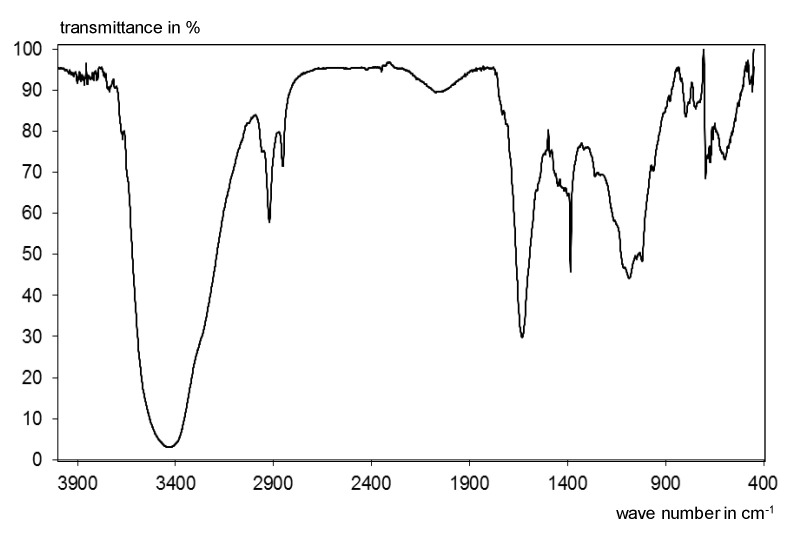
Infrared spectrum of the styrene-butadiene coating of the textile [29].

The textile is a carbon fiber grid with a mesh size of 10.7 mm in warp and 14.3 mm in the weft direction. The textile is coated with 13 to 18 wt.% of a coating agent on a styrene-butadiene rubber base. The corresponding IR spectrum is shown in Figure 4.

### 2.2. Test Specimens

**Figure 5:**
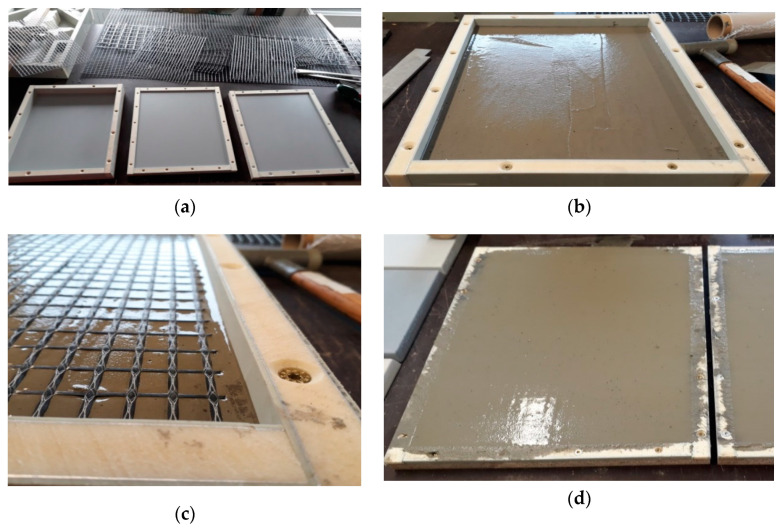

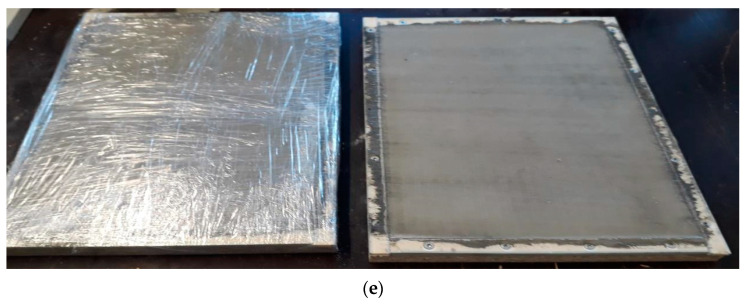
Photo documentation of the test specimens molding process [30]; (**a**) empty formwork and cut textiles; (**b**) first concrete layer; (**c**) textile layer; (**d**) filled formwork; (**e**) sealed (left) and unpacked specimens in formwork.

The test specimens were manufactured by our project partner Hentschke Bau GmbH. For all tests, outdoor, laboratory irrigation, and DSLT tests, the test specimens were laminated into a stainless steel and plastic formwork using pre-cut textile pieces for the reinforcement and polytetrafluorethylene (PTFE) spray as a release agent. After demolding, the samples were wrapped airtight in polyethylene (PE) foil and sealed with adhesive tape to prevent carbonation or moisture loss. The wrapped specimens were stored at 20 °C until the beginning of the testing. Figure 5 summarizes the casting process. Table 5 shows the dimensions and production conditions of the investigated test specimens.

### 2.3. Methods

#### 2.3.1. pH Dependence Test (pH_stat_)

The leaching characteristics of the relevant trace elements from the concrete ready-mix mortar have been determined as a function of the pH value in the range from pH 3.5 to the natural pH of 12.4 according to EN 14429 [31].

Six mortar prisms of 40 × 40 × 160 mm^3^ were produced from the ready mixed concrete and stored sealed in PE foil for 28 days at 20 °C and 65% RH. The prisms were then ground to <1 mm for the testing.

The leaching was carried out using 30 g of the crashed mortar, deionized water and 5 molar HNO_3_ for titration. The eluates were filtered through a 0.45 µm syringe filter from PET and then analyzed by ICP-OES, flame photometry and ion chromatography.

#### 2.3.2. Dynamic Surface Leaching Test (DSLT)

**Figure 6:**
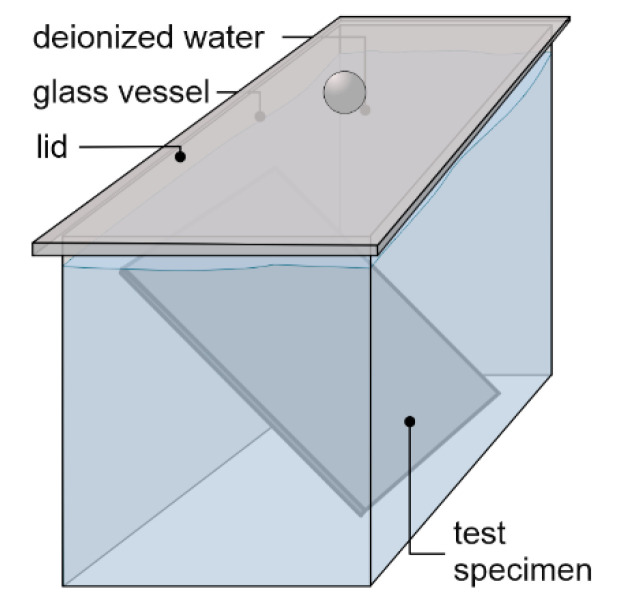
Schematic presentation of the dynamic surface leaching test (DSLT) setup.

The tank leaching tests were carried out by our project partner VDZ gGmbH, based on the European harmonized standard CEN/TS 16637-2 [26]. As pictured in Figure 6, the test specimens were positioned in glass chromatography vessels and then covered with 2.3 L of deionized water and closed with a lid. The ratio of eluate volume divided by the surface of the test specimens (V/A) therefore differed, depending on the thickness of each specimen. The samples of D 1-2 were leached with 47.6 L/m^2^, D 4-2 with 42.1 L/m^2^ and D 1-20 with 29.5 L/m^2^ in each step.

The leaching water was renewed in intervals of 0.25, 1, 2.25, 4, 9, 16, 36 and 64 days, as specified in CEN/TS 16637-2 [26]. From each eluate fraction samples were taken and analyzed. The raw data were provided by the VDZ for further comparison calculations in this work.

#### 2.3.3. Laboratory Irrigation

**Figure 7:**
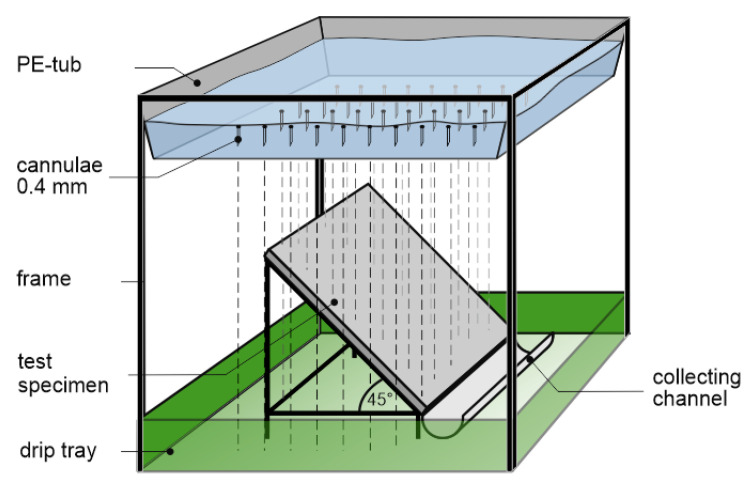
Schematic presentation of the laboratory irrigation stand.

A typical European rain has intensities of 2.5–10 mm/h with drop diameters of 0.5–5 mm, but 1–4 mm for most rain events [32,33,34]. To reach realistic rain scenarios meeting these conditions, the irrigation stand, pictured in Figure 7, has been developed [35].

**Figure 8:**
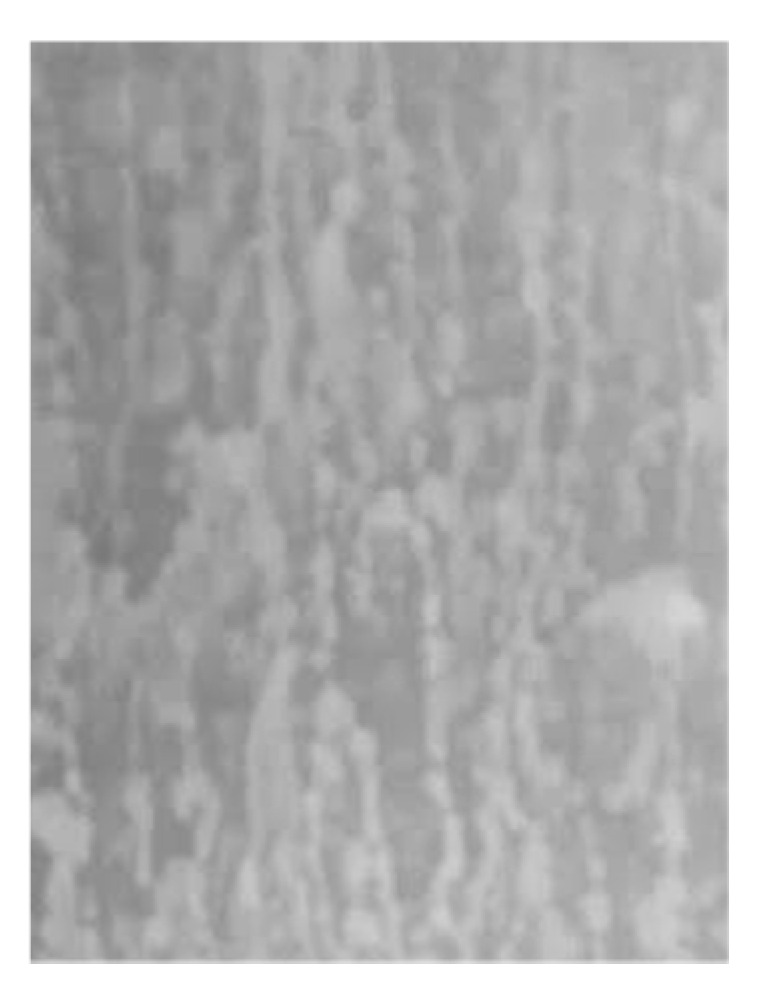
Drop distribution over the test specimen of the laboratory irrigation.

It consists of an irrigation unit made of a PE-tub, equipped with 70, respectively, 100 cannulae evenly spaced out over a base area of 283–300 mm^2^ and a frame to place the sample and collect the runoff. The tub is filled with deionized water to a predefined level. The filling level and the number of cannulae determine the rain intensities. An electric motor moves the irrigation unit every 30 min over 20 mm in a horizontal direction to ensure an equal wetting across the whole test specimen (see Figure 8). The test specimens with dimensions of 300–400 mm^2^ are located in a 45° angle below the irrigation unit.

Pre-tests showed that cannulae of 0.4 mm diameter and 20 mm length are producing constant drops of 2.2 mm diameter and are applicable to create intensities between 1 and 5 mm/h.

The runoff is saved in PE gutters that lead the water to 15 L collection containers made of glass.

**Figure 9:**
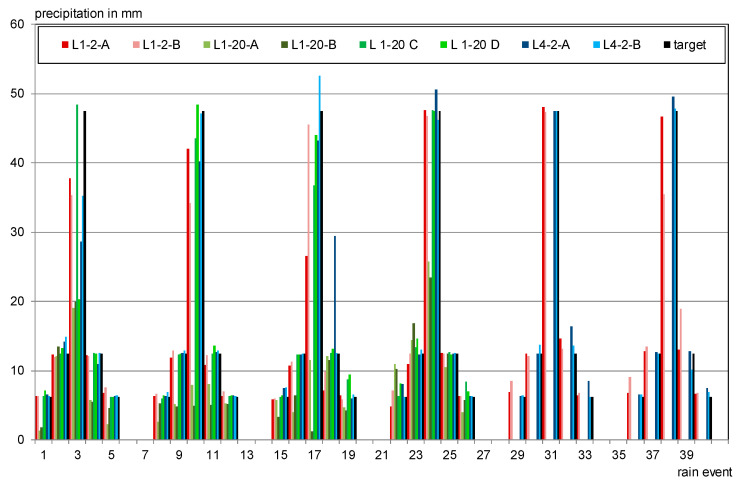
Wetting cycle for the laboratory irrigation.

The target irrigation cycle of the investigations of this work is shown in Figure 9. The cycle has been developed using meteorological data from all over Germany in a previous study [35]. The chosen intensities and amounts of rain are based on weather dates of Holzkirchen for the month of July so the rainiest time and area of Germany is reflected in a testing period of one month.

The desired rain intensities of 1, 2 and 5 mm/h were achieved using filling levels of 4.5 (4.5 mbar pressure), 7 (7 mbar) and 12 cm (12 mbar). Some deviations cannot be avoided due to decreasing pressure over the rain events (especially for high intensities) and due to blocked needles primarily at low pressures. Figure 9 therefore also shows the actual rain cycles that occurred during testing.

The developed method for laboratory irrigation showed well reproducible rain amounts and can be used for further investigations. It turned out to be a relatively simple and cost-efficient test for simulating rain in intensions and drop sizes close to reality. The aimed rain amounts were met with an average deviation of 9.0% (median 3.5%), whereby the high intensities give more weight with 14% as they are not always reached (see Figure 9).

The eluates were homogenized and samples of 3 × 50 mL were taken in PE tubes. One subsample was used to determine pH value and conductivity as well as the concentrations of sodium, potassium and calcium (by flame photometry) and chloride and sulfate (by ion chromatography). The other two subsamples were acidified with 2.5 vol.% supra pure nitric acid. One was used for the analysis of antimony, arsenic, barium, boron, cadmium, chromium, cobalt, copper, lead, molybdenum, mercury, nickel, selenium, thallium, vanadium and zinc (by ICP-MS) the other one was kept as a retain sample. All samples were stored at 4 ± 2 °C until analysis.

#### 2.3.4. Outdoor Testing

For the outdoor testing, the specimens were exposed on a roof in Aachen, Germany (position: 50°46′52.2″ N 6°02′56.8″ E), facing west with a 45° angle to the ground.

**Figure 10:**
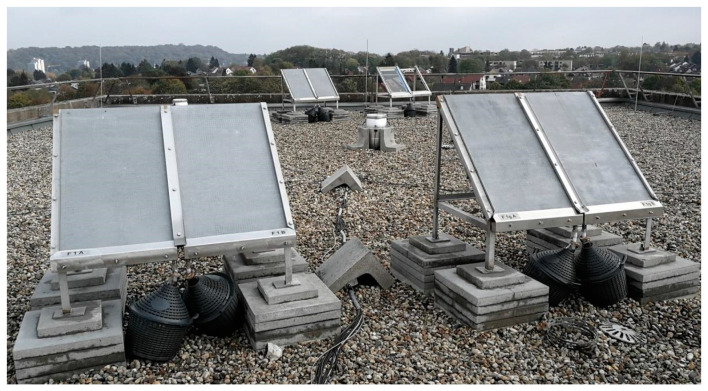
Outdoor testing stands on the roof of the RWTH Aachen University.

**Figure 11:**
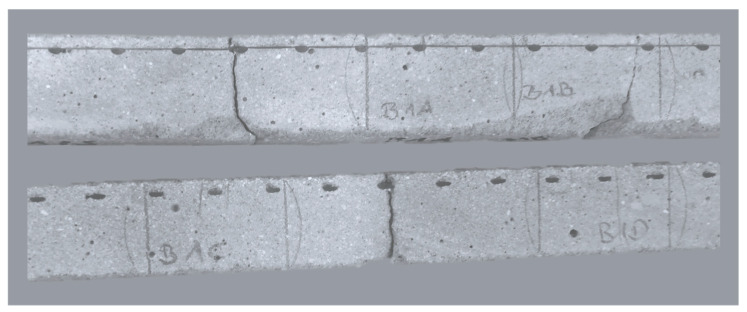
Cracked test specimen F1B.

Each specimen was attached to the sample holder pictured in Figure 10. For the cracked specimens (see also Figure 11), a special clamping was used to keep the cracks open, what should create a worst case scenario with direct contact of rainwater to the coated reinforcement.

The sample holders are made of stainless steel. The runoff from the test specimens is collected in a stainless steel gutter covered with an adhesive PTFE film leading to 15 L, respectively, 25 L glass bottles that are protected from sunlight by a plastic cover.

The leachates of the outdoor weathering were collected in fractions of one week from which the laboratory samples were taken. In case of long-lasting heavy rainfalls, the glass bottles were changed earlier and kept closed and cool until the scheduled change date. Both fractions were then combined for one weekly eluate and the laboratory samples were taken from this mixture.

The analysis preparation and analytics were conducted analogous to the laboratory irrigation procedure.

For the evaluation of the outdoor testing, data from the weather station ‘Aachen Hörn’ (resolution: 10 min) were used [36,37]. The weather data are compared to the amount of leachate collected for every test specimen (wide bar ≙ glass panel).

**Figure 12:**
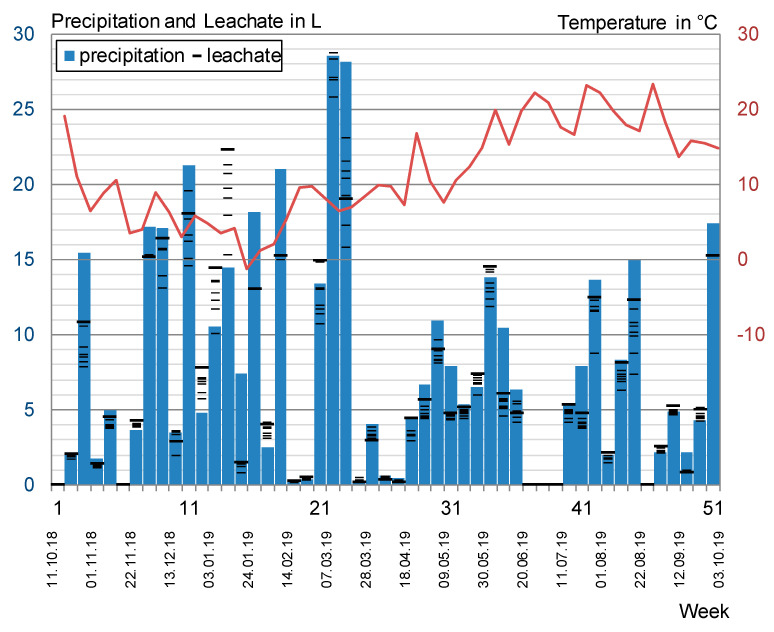
Average temperature, weekly precipitation and collected leachate in L per test specimen.

The wind-driven rain amounts were calculated based on the recorded normal rain amounts after Equation (1) derived from ISO 15927-3 [38].

(1) $$R_{D}=\;\frac{2}{9}\cdot \nu \cdot R_{N}^{\frac{8}{9}}\cdot \cos\left(D-\Theta \right)$$

where *R_D_* = hourly wind-driven rain amount in mm, ν = hourly wind speed average in m/s, *R_N_* = hourly normal rain amount in mm, *D* = hourly average of the wind direction with reference to North in °, Θ = wall orientation with reference to North in °. Figure 12 shows the recorded weekly average temperature and the weekly calculated rain amounts per inclined positioned test specimen over the testing period of one year.

Due to the prominent position and the proximity to the weather station, no topography factors were applied. Furthermore, as they are not hitting the surface and therefore are not contributing to leaching, negative wind-driven rain amounts were considered as zero.

The test specimens were irrigated 9.6% of the time. The yearly normal rainfall sums up to a total amount of 718 mm, leading to a calculated amount of 398 L/a per test specimen of which 305 L are attributed to normal and 93 L to wind-driven rain.

The collected leachate amounts varied from 75% (F1A) to 90% (glass panel), on average 80%, of the calculated precipitation, showing a decreasing tendency over the testing period. Moreover, 0% was collected for weekly precipitation sums of <0.2 L/m^2^; Eluate amounts of >100% of the calculated rain amounts are attributed to mainly snowfall (week 12 to 14) and probably imprecise calculated wind-driven rain. The detailed results for the amounts and analysis of the collected eluates are shown in the electronic supplement.

Apart from one incident of frost-damaged bottles (Series No. 14) and three overflow events (Series No. 16, 20, 49), all eluates were collected steadily over the testing period of one year from 11 October 2018 to 10 October 2019. The conformity of the collected leachate amounts and analyzed concentrations is considered as appropriate for an outdoor experiment.

### 2.4. Testing Program

To determine the influence of the reinforcement, especially its coating, test specimens with different concrete coverings were examined in double determination for both laboratory tests. Under outdoor conditions also cracked test specimens (F 1g and F 4g) were investigated. The direct contact of rainwater with the reinforcement might have an influence on the leaching behavior. Table 6 gives an overview over the testing programs conditions.

Due to technical reasons the specimens of the laboratory irrigation were tested at different ages: L1-20 A + B, 15 weeks; L1-2 20 weeks; L4-2 25 weeks. To detect differences caused by age and to investigate the repeatability of the method, a second run with L 1-20 test specimens (samples C and D) at an age of 8 weeks was conducted. In addition, the test specimens L 1-2 and L 4-2 were irrigated for two more weeks after a drying phase of half a year to find out about the long-term behavior and possible influences of carbonation.

## 3. Results

### 3.1. Preliminary Note

To give a first statement concerning the environmental compatibility of C^3^, the concentrations in the collected eluates and leachates, which could have an immediate impact on the environment, are taken into account. The concentration development is investigated regarding indications for prevalent leaching mechanisms and influencing factors determining the emissions for the respective leaching experiment. The cumulated emissions and further assessment approaches are discussed in part 2 of this publication.

### 3.2. Overview of Eluate Concentrations in the Different Tests

Four variants of the same material have been leached using four different methods, resulting in different eluate concentrations. Table 7 gives an overview over the range of the respective concentrations. The detailed test results and further comparisons are given in the following chapters. All measured eluate and leachate concentrations are given in the electronic supplement, Tables S1–S48.

As expected, the maximum concentrations of all substances are obtained in the pH_stat_ test. This test method is used to determine the respective solubility. The concentrations are significantly higher than in the other leaching experiments, indicating a dilution in the leaching tests on the monolithic samples. Outdoor and laboratory irrigation as well as the DSLT show different relations among each other, dependent on the observed substance.

**Figure 13:**
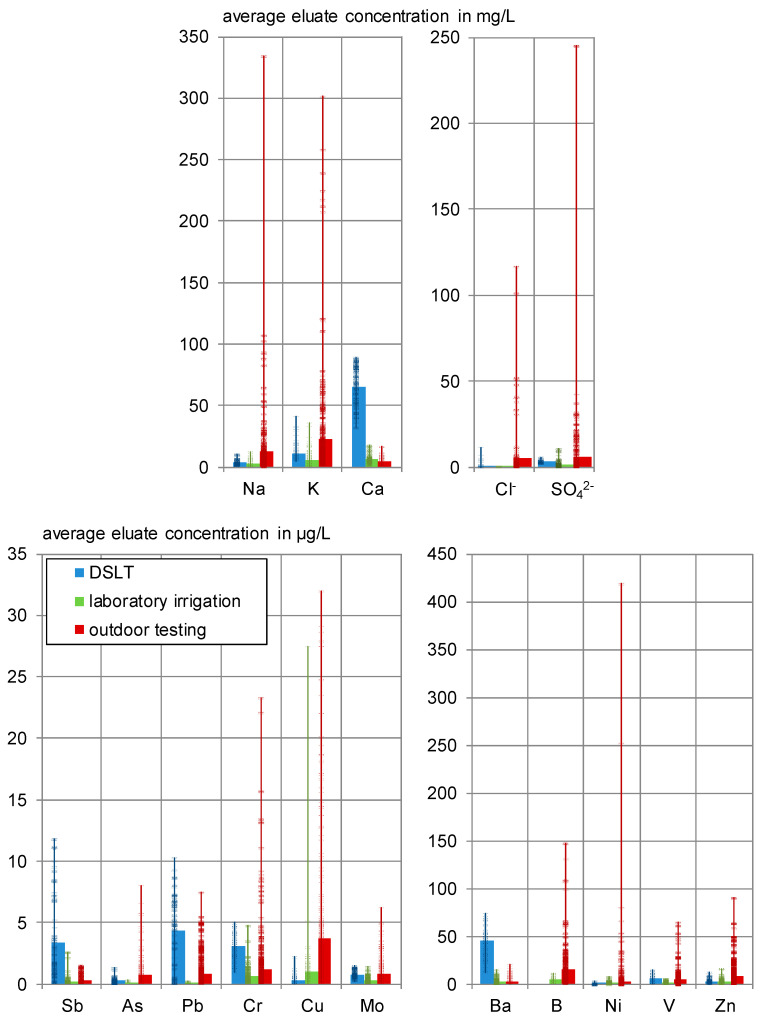
Average eluate concentrations and value distribution of DSLT, laboratory irrigation, and outdoor testing.

High concentrations in comparison to the average, especially for the outdoor irrigation, are often reached only once or twice a year and mainly at the beginning of the test. This is emphasized by Figure 13, showing the average runoff concentrations and their value distribution for the analyzed elements. Some elements are not displayed in Figure 13 and are also excluded from further evaluation due to low prevalence or high concentrations in the blind tests:

- Cadmium and thallium (determination limit: 0.1 µg/L) could not be detected in any eluate in the laboratory irrigation and were detected in maximum 10% of the leachates due to the background concentration outdoor. They were at no time leached from the specimens.
- Cobalt (determination limit: 0.1 µg/L) could not be detected in any eluate in the laboratory irrigation and was found in 92% of the outdoor leachates, always in the range of the background concentrations. A slightly increasing tendency was observed outdoor.
- In the laboratory mercury was detected in concentrations close to the detection limit (0.01 < c ≤ 0.023 µg/L) in 29% of the eluates. Five of eight test specimens (L 4-2 A + B, L 1-2 A, and L 1-20 C + D) and the outdoor experiments showed no detectable mercury leaching at all.
- Outdoor antimony (detected in 74% of the eluates), lead (93%) and zinc (100%) were released from C^3^ in only 6 to 10% of the samples.
- Chloride (determination limit: 0.1 mg/L) was detected in concentrations of 0.1 < c ≤ 0.4 mg/L in only 13% of the laboratory eluates. In 94% of the outdoor leachates, emissions with an average concentration of 2.28 mg/L were measured.
- Copper and zinc showed relatively high releases in the laboratory irrigations’ blind testing, which has to be considered in the interpretation of the results. It is assumed that zinc as a ubiquitous element is introduced through several pathways. Copper could be leached from the cannulae that are indicated as stainless steel. This steel might have copper residues or is alloyed with it to enhance corrosion and acid resistance [39].
- Arsenic, lead and selenium were detected in 18% (As, Se), respectively, 7% (Pb) of the laboratory eluates in concentrations of 0.1 < c < 0.3 µg/L. Outdoors, arsenic is leached and lead is adsorbed. Selenium is mainly leached in concentrations below 0.1 µg/L.
- Sodium, potassium, calcium, barium, boron, chromium, and vanadium were leached in 70 to 100% of the outdoor samples and detected in 100% of the laboratory eluates.

The substances named in the last two points are therefore main subjects to further interpretation.

Neither the number of reinforcement layers nor the thickness of the concrete cover showed significant influence on the leaching behavior [27,35]. Figure 10 and the figures for the discussion therefore show the averaged substance release of the different test specimens.

### 3.3. Laboratory Irrigation

**Figure 14:**
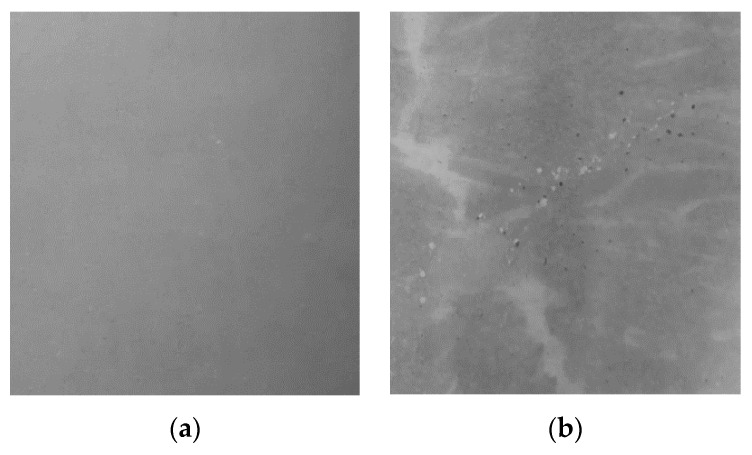
(**a**) Surface of test specimen L1-20 A before the laboratory irrigation; (**b**) surface of test specimen L 1-20 A after 4 weeks of laboratory irrigation.

During the laboratory irrigation, only slight changes in the test specimens’ surface were observed. In the first week of the testing, a droplet pattern developed, lightening up the surface when converging over time. Figure 14 exemplarily shows the smooth surface of specimen L 1-20 A before the testing and the surface after 4 weeks of irrigation, which led to a slightly rougher surface and a few calcite-filled pores.

**Figure 15:**
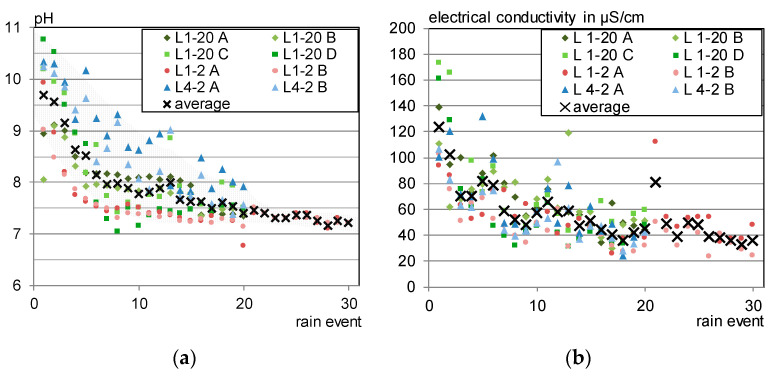
(**a**) Development of the eluate pH in the laboratory irrigation experiment; (**b**) development of the electrical conductivity in the laboratory irrigation experiment.

The eluate pH development differed slightly between the test specimens as can be seen in Figure 15. All tests show a decreasing pH over the testing period. Most eluates had a starting pH of 10 to 11, two started at around pH 9 and one at only pH 8. This might be a consequence of inaccurate sealing and therefore pre carbonation of the test specimens’ surface. The different ages of the samples had no systematic effect on the pH. It is noticeable that the specimens starting at a lower pH show a more stable and constantly decreasing development than the specimens coming from a higher pH. All eluates level at a pH around 7.5 after three weeks of irrigation, which also continued in the subsequent irrigation after six months.

The conductivity of all eluates varied according to the amount of water applied in one step and showed a decreasing tendency from 100 to 180 µS/cm in the first week to 30 to 60 µS/cm in the fourth week of testing.

**Figure 16:**
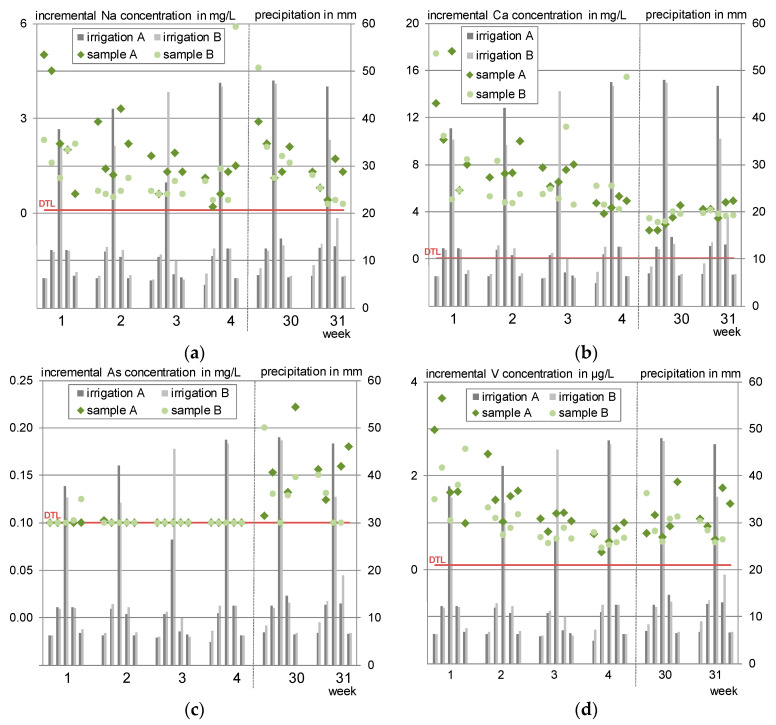
Incremental eluate concentrations of the test specimens L 1-2. (**a**) sodium; (**b**) calcium; (**c**) arsenic; (**d**) vanadium.

Figure 16 exemplarily shows the course of the incremental concentrations measured in the eluates for sodium, calcium, arsenic and vanadium.

A decreasing tendency in the eluate concentrations can be observed for all substances over the initial testing period of four weeks. Leaching of arsenic could not be detected during these four cycles. The concentrations of all substances are lower when more water is applied which shows dilution and is hinting at a diffusion-controlled leaching. However, calcium appears to level at concentrations around 4 mg/l after three weeks.

Contrary to the conception expectation that drying phases will increase the following eluate concentrations, the scheduled weekly irrigation breaks do not lead to changed, higher, concentrations compared to the rain event with the same conditions of the previous cycle. Only the long drying phase leads to increased concentrations for all shown substances except for calcium, suggesting that the conception of the drying phases is too short or rather not forceful enough to depict effects that are usually evoked by intermittent wetting.

A change in pH by further advanced carbonation processes can be excluded as a cause for the course changes from week 30. Figure 15 pictures the very similar pH of 7.5 before the break, showing that the samples are already carbonated, dropping to minimum pH 7.2 after the break. Moreover, this slight difference would not affect the examined substances even if they were leached pH dependent and close to their maximum solubility.

### 3.4. Outdoor Testing

**Figure 17:**
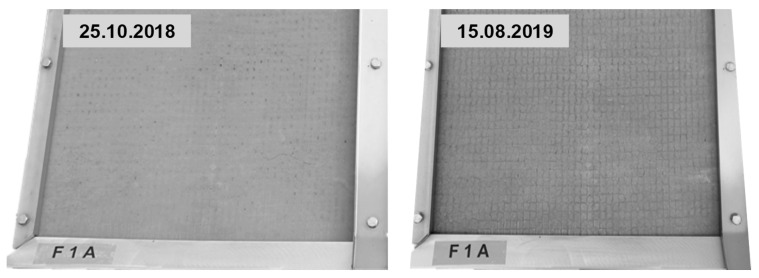
Surface of outdoor test specimen F1A after two and 44 weeks of exposure.

All outdoor test specimens developed the same optical changes over the time of exposure, pictured exemplarily for test specimen F1A in Figure 17.

After one year of weathering, the surface appeared more porous and the reinforcement which was covered by only 2 mm of concrete became clearly visible. Some pores were filled with calcite. The dark lines above the textile are attributed to water flow along the reinforcement due to less suction depth of the concrete and probably cavities next to the textile.

**Figure 18:**
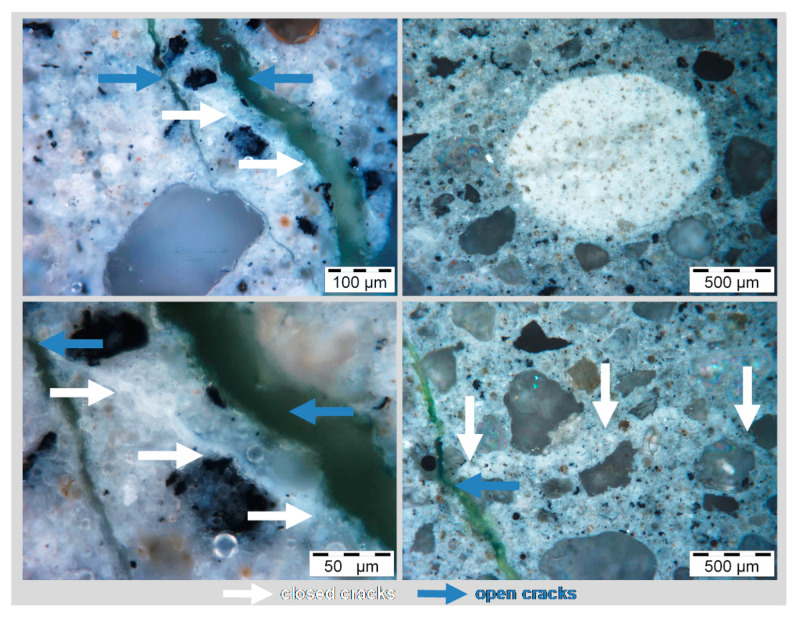
Cross section of tests specimen F1gB after one year of exposure; closed cracks and pores. Blue: main cracks, open; white: transverse cracks, closed.

Apparently, the crack width of the cracked surfaces remained constant over the testing time. It was measured in week 4, 8 and 44 with a crack measuring gauge. Nevertheless, light microscope images taken from cross sections after the test revealed that micro cracks, positioned transversely towards the main cracks, and also pores were closed over time as can be seen in Figure 18.

**Figure 19:**
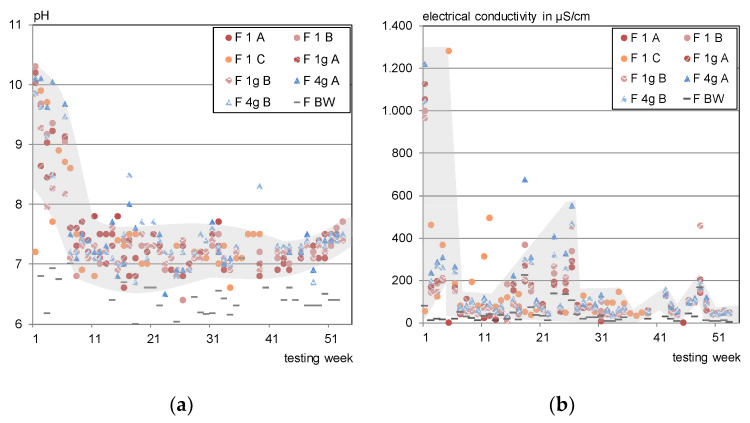
(**a**) Eluate pH in the outdoor testing; (**b**) electrical conductivity in the outdoor testing.

The eluate pH, imaged in Figure 19a, developed the same way for all test specimens. It started at around pH 10 and dropped to pH 8 within the first 5 weeks, after a drying phase it increased to pH 9.5 again but levelled at pH 7 ± 0.75.

The conductivity of the eluates varied as per Figure 19b. Except from the first rain events (1000 to 1200 µS/cm dropped to 200 to 400 µS/cm), no decreasing tendency was observed.

**Figure 20:**
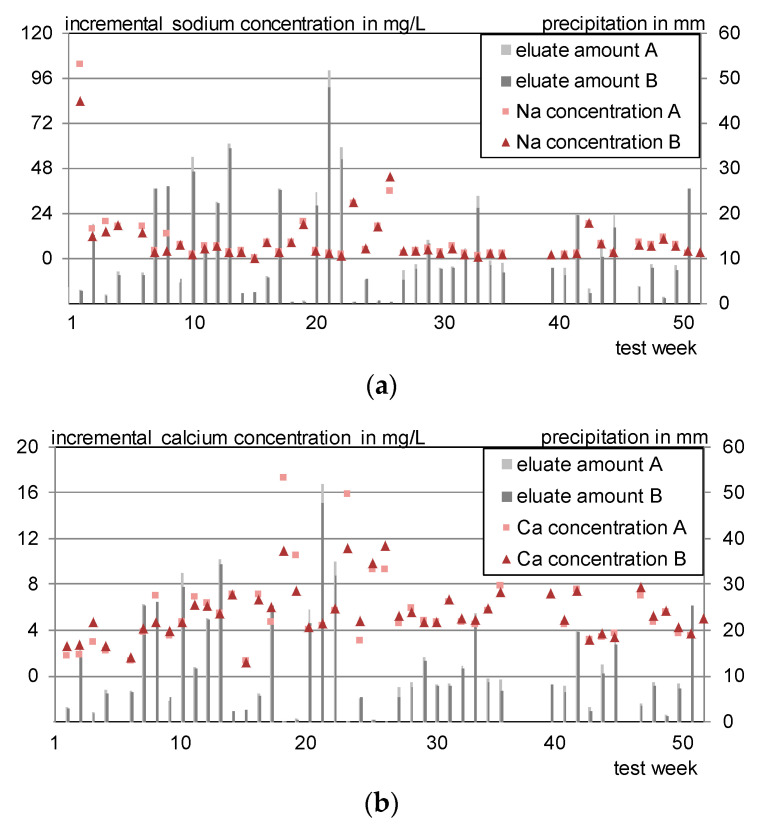

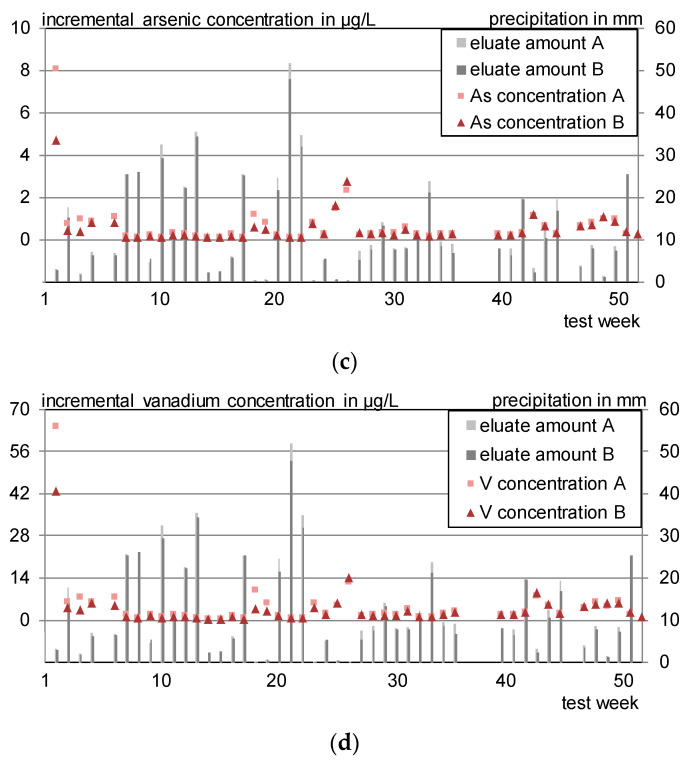
Incremental leachate concentrations of the test specimens F 1g. (**a**) Sodium; (**b**) calcium; (**c**) arsenic; (**d**) vanadium.

Figure 20 exemplarily shows the individual concentrations of sodium, calcium, arsenic and vanadium for the outdoor test specimen F 1g A and F 1g B. Sodium, arsenic and vanadium developed a nearly identical concentration pattern. Sodium shows a slightly higher response to dry or semi-dry phases, which is attributed to its lower bonding to the concrete matrix.

Calcium concentrations are increasing in the beginning and level, similar to the laboratory irrigation, between 4–8 mg/L. (Higher concentrations in cases of very low rainfall can be assigned to high blank values.) 

### 3.5. Standardized Leaching Tests

In the DSLTs, the eluate pH for all test specimens was stable at 11.2 to 11.8 with a maximal difference of 0.3 between the specimens of one composition.

The established DSLT is based on a water-to-surface ratio of 80 L/m^2^. The amount of water applied in the tests described in this work was lower (D 1-2 47.6 L/m^2^, D 4-2 42.1 L/m^2^), which is irrelevant in this tests, as long as the release is controlled by diffusion. However, if the pH is significantly different or concentrations reach equilibrium, the release will be changed.

To determine the leaching mechanism of the evaluated substances under standardized laboratory conditions, the evaluation method described in [26] was used. If the leaching process of the DSLT is diffusion controlled, granted that the diffusion coefficient does not change over the process, the release is in a linear relation to the root of time. The defined leaching steps lead to eluate concentrations that allow for distinguishing the leaching mechanism, mainly diffusion or solubility.

**Figure 21:**
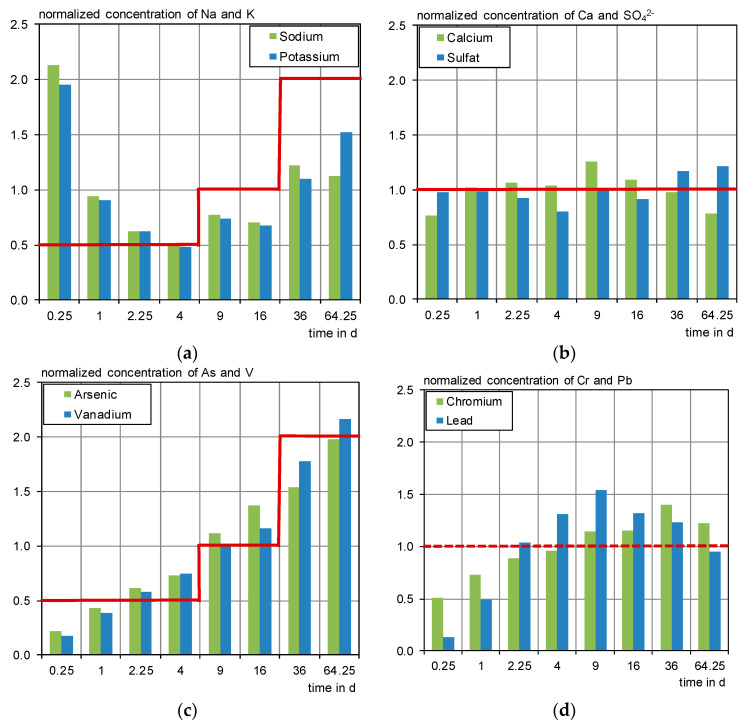
Leaching mechanism, three-step diagram after CEN/TS 16637-2. (**a**) Sodium and potassium, diffusion controlled with initial wash-off effect; (**b**) calcium and sulfate, solubility controlled; (**c**) arsenic and vanadium, diffusion controlled; (**d**) chromium and lead, no mechanism determined.

For the evaluation, a normalized concentration is calculated. The eluate concentrations of each step are divided by the averaged concentration of all steps and plotted as a column diagram. A diffusion-controlled process will picture a three-step diagram (see Figure 21c), while a solubility-controlled process shows a continuous normalized concentration of one (see Figure 21b) as it is constantly leached in case of a stable pH.

It is obvious that the described pattern is never exactly met and some mechanism overly each other. This is a known phenomenon; the criteria for a diffusion-controlled process are therefore defined as valid for the root of the mean square error ($\sqrt{MSE}$) below 0.4 for each eluate concentration [26].

For the evaluation of the DSLT, the results of D 1-2 and D 4-2 were averaged and subsequently analyzed following Annex B of CEN/TS 16637-2 [26]. Sodium, potassium (Figure 21a), arsenic and vanadium (Figure 21c) were determined as diffusion controlled, whereas the alkalis seem superimposed by an initial wash-off effect. Consistent with the findings of [40], calcium and sulfate leach mainly solubility controlled (Figure 21b). In this work, the standard deviations between the single eluate concentrations were >0.25; the leaching mechanism is therefore not calculative affirmed within the meanings of [26]. For chromium and lead (Figure 21d) no leaching mechanism can be identified according to [26]. In [40], chromium is found to be diffusion controlled, lead is depleting over the duration of the tank test. Looking at the normalized concentrations, it is assumed that chromium and lead leach diffusion controlled followed by depletion or by equilibrium concentrations.

**Figure 22:**
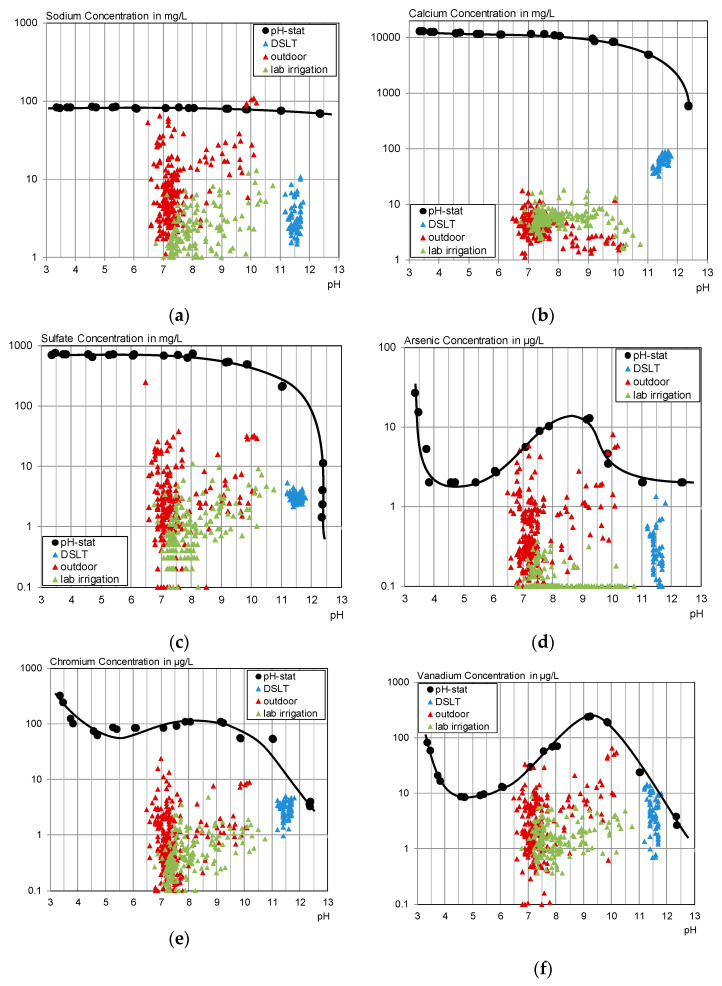
pH-dependent leaching of selected substances from C^3^ compared to the leachate concentrations of the DSLT, the laboratory irrigation and the outdoor testing. (**a**) Sodium; (**b**) calcium; (**c**) sulfate; (**d**) arsenic; (**e**) chromium; (**f**) vanadium.

One approach to check if concentrations are approaching equilibrium is to compare them to the concentrations reached in the pH_stat_ test. The pH_stat_ test (2.3.1) indicates the solubility of the respective elements at different pH values. Figure 22 pictures the results of the pH dependence test compared to the concentrations of the other leaching experiments of this work.

For permanent water contact, it becomes visible that the concentrations of the solubility-controlled substances calcium and sulfate remain in a closer range compared to the concentrations of the other substances. However, they stay clearly below their possible maximum concentrations. This might be an effect of leaching steps that are not long enough to reach equilibrium concentrations combined with decreasing concentration gradients due to physical barriers through the concrete.

Only vanadium, and, considering analytical and computational uncertainties, possibly chromium, reached the determined pH_stat_ concentrations in the last two time steps of some DSLTs. A higher water to surface ratio would probably lead to a higher total release at the same or lower concentrations in this case.

During intermittent wetting in the laboratory, a concentration gradient seems to be kept for all substances. The concentrations are mainly below or in the range of the DSLT.

Outdoors, all concentrations except for calcium exceed both laboratory experiments’ concentrations, which is partially a result of the rainwater contamination. However, the net values between the test specimens’ concentration and the glass panel results show that the outdoor leaching sometimes leads to significantly increased leaching compared to the laboratory testing. This can be a result of harsher conditions as the material experiences longer drying phases, wind is carrying moisture from the surface, and temperature changes contribute to faster drying. However, it also has to be considered that the outdoor specimens were larger than the laboratory ones, which might extend the contact time for each droplet. For diffusion-controlled leaching, a longer time span results in higher concentrations.

- Sodium leaches strongest under outdoor conditions, which confirms the findings concerning the influence of dry phases from the laboratory irrigation. It is assumed that capillary transport mechanisms transport dissolved substances to the surface as described in [11,13] and therefore cause an increased availability of sodium on the test specimens’ surface.
- Sulphate also shows higher outdoor concentrations but considering the blank, the net concentrations of sulphate were mainly below the DSLT and in the range of the lab data.
- Calcium concentrations show a good concordance between laboratory and outdoor irrigation.
- Arsenic, chromium and vanadium leach more strongly in the outdoor irrigation compared to the laboratory, especially for higher pH values.

There is no general correlation between the laboratory leaching data and outdoor emissions. A direct prediction of the outdoor emissions seems only possible for solubility-controlled substances with low background concentration in the eluent.

## 4. Discussion

**Figure 23:**
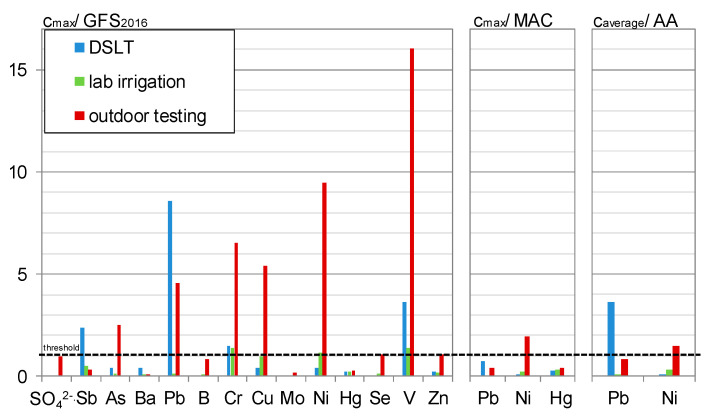
Maximum leachate concentrations related to the de-minimis thresholds after [41] and [6].

To classify the measured concentrations and allow a statement concerning the environmental compatibility of C^3^, the results are compared to reference values. Figure 23 shows the maximum concentrations reached in the irrigation experiments related to current threshold values of Groundwater in Germany [41] and for surface water bodies in Europe [6]. The concentrations of the DSLT eluates are pictured comparatively.

The MACs (maximum allowable concentrations) and AAs (annual averages) after Article 3, Point 1, together with Part A of Annex I of the European directive on environmental quality standards for surface water qualities [6,42] only applicable for lead, nickel, and mercury, are only exceeded for nickel during the outdoor investigation. The MAC of 34 µg/L was exceeded in 3% of the time, the annual average is at 110% of the threshold of 4 µg/L. Considering the average background concentration of 1.3 µg/L in the rainwater, this exceedance is not only attributable to the C^3^. A direct discharge to a surface water compartment can be considered without negative effects.

As pictured in Figure 20, during the laboratory irrigation, only chromium (1.25% of 160 eluates), nickel (1%), and vanadium (5%) occasionally exceeded the de-Minimis threshold for groundwater of the German Working Group on water issues of the Federal States and the Federal Government represented by the Federal Environment Ministry (LAWA) [41]. Since the experiment was conducted using aggressive deionized water and furthermore the thresholds are designed for groundwater not seepage water, a direct infiltration in the soil is considered to be environmentally harmless.

Outdoor, arsenic (4%), lead (25%), copper (17%), nickel (14%), vanadium (32%), and zinc (1%) were measured in higher concentrations, whereby lead, copper, and zinc concentrations originated only from the rain water and were even adsorbed by the concrete. These findings are similar to a study of Schiopu et al., where leachate concentrations from irrigated concrete slabs were only higher than the rainwater concentrations in 47%, respectively, 21% of the eluates for copper and zinc [24].

Arsenic, nickel and vanadium are leached from the C^3^ and contribute to the rainwater discharge concentrations. It has to be taken into account that exceeding the threshold values after [41] means, that a direct entry into the groundwater in high amounts could possibly affect the environment. The retention by the covering soil layers and the dilution with unaffected rainwater leads to decreasing concentrations.

Generally speaking, it is a known problem that municipal rainwater discharge contains, amongst others, too much heavy metals and trace elements. The study of [3], for example, showed that all heavy metals except from vanadium and nickel exceed the MACs and AAs in the rainwater runoff in Berlin.

Furthermore, it has to be mentioned that it is an internationally discussed topic for currently more than 10 years to except concrete from further testing on the release of regulated dangerous substances. Schiopu et al., for example, studied irrigated concrete slabs and only found leachate concentrations close to the rainwater concentrations for sodium, potassium, calcium, sulfate, copper, boron and zinc [24]. Table 8 shows the results of this work for the critical substances chromium and vanadium compared to the concentrations measured in the studies of Scherer and Vollpracht [15,21,43], to a study of Schiopu [44] and to the collection of concrete and mortar DSLT-Data by Dijkstra and v. d. Sloot from the project ECRICEM [45]. The dossier [45] was intended as a proposal for the without further testing (WFT) qualification of concrete whereby the DSLT values should confirm these properties. C^3^ is clearly below the maximum values of [45] and shows low concentrations compared to [21,43].

Considering the outdoor data of Table 8, a contribution from C^3^ to the total load might only be assumed for vanadium. Looking at the raw materials’ vanadium content (Table 4), the vanadium is most likely to be leached from the cement not the reinforcement.

After the comparison to general and actual rainwater background concentrations, German and European ground and surface water thresholds, as well as other mineral building products, it can be concluded that the concentrations, measured for all leaching experiments conducted, showed no environmental critical results. However, it also has to be mentioned that any emission from any material might be of concern as it is contributing to the total contaminant load and fostering potentially harmful concentrations in the rainwater discharge. The reduction and prevention of substances release must stay a main objective.

## 5. Summary and Conclusions

The use of composite building materials is currently increasing, which leads to the use of new materials or material combinations with unknown leaching behavior. Mutual influences or reactions of combined substances can change emissions despite an unchanged content level. To avoid a possibly harmful release of substances from the outset, it is necessary to prove their environmental compatibility.

In this research, the leaching behavior of C^3^, especially for the application as irrigated façade elements, was investigated. Laboratory and outdoor exposure tests were run to determine and assess the heavy metal and trace element emissions.

A laboratory irrigation stand and an outdoor experimental setup with reproducible result production were introduced. Investigations through this stand on C^3^, compared to each other, to the standardized laboratory test DSLT, and to reference values, indicated that this innovative composite material as environmentally compatible concerning its heavy metal and trace elements leaching behavior. Further research on organic trace substances, as, e.g., bisphenol A used as a raw material or amines used as hardeners in epoxy resins, would complement this classification.

The concentration thresholds for German groundwater [41] and the EU surface waters [6,41] were exceeded by arsenic, nickel and vanadium at times; only vanadium leached more strongly than an average concrete. In comparison to leachate concentrations of other irrigated mineral building materials, C^3^ is neglectable. Moreover, zinc, lead, and copper were found not to be leached but rather to be adsorbed from concrete construction elements. As these substances are the ones with the comparably highest concentrations in rain waters, building materials other than concrete, for example, metal sheets or renders, must be considered as a source of the emissions from façades described in [3]. Considering the data of [45] for the WFT qualification of concrete reinforces this assumption; although it has to be kept in mind that this study is related to DSLT results while intermittent wetting causes altered leaching behaviors.

The non-traditional reinforcement showed no discernible influence on the leaching behavior of the substances observed. However, different leaching patterns, dependent on outdoor factors, could be identified for different elements, providing a foundation for further assessment method development.

In addition to the leaching behavior of C^3^, the validity of laboratory experiments and the transferability to the outdoor behavior were investigated. No general correlation between laboratory leaching data and outdoor emissions were found. The results depend on the examined substance and the method used.

For further research regarding the laboratory irrigation, the wetting cycles have to be optimized to represent outdoor influencing factors. Although the test delivered reproducible results, the outdoor behavior was not sufficiently reproduced. The full irrigation cycle as a time- and effort-saving prediction method is not practicable and does not seem expedient. However, it could be used to vary single factors and better understand outdoor leaching behavior. In [46], a 24 h weathering cycle for the leaching of organic materials is introduced by Bandow et al. As inorganic constituents are partially incorporated into the leached material and generally show another emission behavior than organics, an adaption on concrete would have to be evaluated.

A transfer function or model to predict outdoor emissions of irrigated building materials from laboratory data would be desirable for a fast and reasonable assessment of materials that are of concern for the environment. In part 2 of this research, this topic will be debated. A first step towards an assessment method would be to check existing assessment concepts on their suitability by using the data collected for this work. If there is no direct application possibility, the investigation of the transferability, for example, by transfer functions, would be a further approach. Lastly, if necessary, a new approach on how to estimate outdoor emissions based on known leaching mechanisms and present data has to be developed.

## Figures and Tables

**Table 1:** Overview of previous research projects with laboratory irrigation.

Leached Material	Investigated Emissions	Irrigation Unit	Intensity and Irrigation Procedure	Reference
concrete façades/exposed concrete	chromium, zinc	plastic tray with perforated bottom	25 mm/h,1.5 h rain, 2 h break, 1.5 h rain, 37 h drying time, total: 650 mm	2005 [11]
mineral construction materials	sulfate, chloride, fluoride, cyanide, 16 trace elements	spray mechanism (compressed air + nozzle)	0.7–5 mm/h, finely dispersed droplets (mist)	2008 [9]
synthetic resin render with biocides	biocides	weathering chamber	85 mm/h, 20 irrigation intervals of 1 h in 5 d, T = 50 °C–60 °C, total: 6800 mm	2009 [12]
renders/render-paint-systems with biocides	biocides	“water pressure and flow rate were controlled”	75 mm/h, 2 min/d	2009 [13]
metal building materials (stainless steel, copper, zinc)	heavy metals, trace elements	spray mechanism (compressed air + nozzle)	0.7 mm/h–3.5 mm/h, finely dispersed droplets (mist)	2011 [14]
renders, mortars	sodium, potassium, sulfate, chloride, 10 trace elements	spray mechanism (compressed air + nozzle)	0.7–5 mm/h, total: 60 mm, finely dispersed droplets (mist)	2012 [15]

**Table 2:** Mineralogical analysis of the sieve fractions of the ready-mixed dry concrete.

Component	Content of the Sieving Fractions in wt.%					
	0–0.063 mm	0.063–0.125 mm	0.125–0.25 mm	0.25–0.5 mm	0.5–1 mm	1–2 mm
C_3_S	30	31.4	–	–	–	–
C_2_S	13.8	20	–	–	–	–
C_3_A	5.8	6.7	–	–	–	–
Brownmillerite	1.3	1.2	–	–	–	–
Quartz	1.8	25.6	85.3	90.9	91.9	93.9
Ca-langbeinite	0.5	–	–	–	–	–
Anhydrite	4.3	2.5	–	–	–	–
Hemihydrate	0.6	0.3	–	–	–	–
Gypsum	0.8	0.9	–	–	–	–
Calcite	1	1.4	–	–	–	–
Portlandite	0.2	0.4	–	–	–	–
Mullite	3	6.3	–	–	–	–
Hematite	0.4	0.9	–	–	–	–
Microcline	–	2.3	9.7	7.2	5.8	4.1
Fluorphlogopite	–	0.3	0.3	0.5	0.3	–
Anorthite	–	–	3.8	1.4	2.1	2
Clinochlore	–	–	0.8	–	–	–
Pseudobrookite	–	–	0.1	–	–	–
Amorphous	36.5	–	–	–	–	–

**Table 3:** Main components of the <125 µm fraction of the concrete ready mix.

Parameter	Content in wt.%
loss on ignition	2.47
insoluble in HCl	26.24
SO_3_	2.08
Na_2_O	0.39
K_2_O	1.13
chloride	0.036
MgO	0.96
Al_2_O_3_	9.59
SiO_2_	36.57
P_2_O_5_	0.29
CaO	44.37
TiO_2_	0.48
MnO	0.05
Fe_2_O_3_	2.63

**Table 4:** Contents of heavy metals and trace elements of the raw materials.

Parameter	Concrete Mixture [27]	Fine Fraction (<125 µm)	Reinforcement Textile Incl. Coating [27]	Average of German Cements [28]
	mg/kg			
antimony	1.1	1.50	0.9	2.9
arsenic	7.2	13.0	2.1	7
barium	252	295	11.0	-
lead	26.5	19.9	0.9	17
cadmium	0.3	0.226	<0.1	0.4
chromium	64.4	27.2	206.0	41
cobalt	6.9	6.84	2.4	8.7
copper	32.1	15.5	45.7	31
molybdenum	3.7	4.39	4.8	-
nickel	38.9	20.5	57.9	23
mercury	0.1	0.068	<0.02	0.06
thallium	1.5	0.275	<0.05	0.4
vanadium	52.8	60.7	1.25	50
zinc	63.1	44.4	93.8	192

**Table 5:** Dimensions and production conditions of the investigated test specimens [30].

Testing Method	Label *	Width/ Length	Thickness	Layers of Reinforce-Ment	Concrete Cover	Room Temp	Concrete Temp	Flow Spread
		mm	mm	n	mm	°C	°C	mm
DSLT	D1-1-2 **	150/150	5.50	1	2	20.2	16.5	270
	D1-4-2 **	150/150	16.0	4	2	21.3	17.3	260
	D1-1-20 **	150/150	40.0	1	20	22.0	17.8	260
lab irrigation	L 1-2 A	300/400	5.50	1	2	20.2	16.8	275
	L 1-2 B	300/400	5.50	1	2	19.8	16.6	250
	L 4-2 A	300/400	16.0	4	2	21.6	17.0	255
	L 4-2 B	300/400	16.0	4	2	21.3	17.3	260
	L 1-20 A	300/400	40.0	1	20	22.3	17.5	270
	L 1-20 B	300/400	40.0	1	20	22.8	17.1	265
	L 1-20 C	300/400	40.0	1	20	22.5	24.2	230
	L 1-20 D	300/400	40.0	1	20	23.1	23.3	237.5
outdoor testing	F 1 A	1000/600	20.0	1	2	20.2	21.9	205
	F 1 B	1000/600	20.0	1	2	21.7	24.2	210
	F 1 C	1000/600	20.0	1	2	n. d.	n. d.	n. d.
	F 1g A	1000/600	20.0	1	2	22.0	23.5	247.5
	F 1g B	1000/600	20.0	1	2	21.9	23.1	242.5
	F 4g A	1000/600	20.0	4	2	21.9	23.2	235
	F 4g B	1000/600	20.0	4	2	21.2	23.4	230

* Label systematics: Exposition—Level of Reinforcement—Concrete Cover (no number ≙ 2 mm)—Replicate. ** Three test specimens each.

**Table 6:** Testing program conditions.

Characteristic	DSLT	Laboratory Irrigation	Outdoor Testing
blank	empty leaching vessel	irrigated glass panel	irrigated glass panel
duration	64 d	28 d (+12 d)	365 d
total amount of water	240 to 380 L/m^2^	344 L/m^2^	663 L/m^2^ *
conditions	permanent water contact	scheduled irrigation and drying phases; drop size: about 2.2 mm; intensities: 1, 2, 5 mm/h	outdoor conditions 45° angle to ground facing west

* calculated amount of water, that hit the test specimens.

**Table 7:** Concentration range in the leaching tests.

Substance	Concen-Tration	pH_stat_		DSLT		Laboratory Irrigation		Outdoor Irrigation		Outdoor Background	
		Min	Max	Min	Max	Min	Max	Min	Max	Min	Max
Na	mg/L	68	83	1.46	10.6	<0.2	12.7	0.4	334	<0.1	18.4
K		136	174	4.69	41.2	0.8	35.4	1.0	302	<0.1	2.2
Ca		4830	12,700	32.0	88.7	1.6	17.6	1.1	17.2	<0.1	9.4
Cl^−^		0.2	14.3	<0.1	11.3	<0.1	0.4	<0.1	117	<0.1	39.4
SO_4_^2−^		1.4	742	2.1	5.3	1.2	10.9	<0.1	245	<0.1	19.0
Sb	µg/L	n.d.	n.d.	<0.1	11.8	<0.1	2.58	<0.1	1.52	<0.1	1.03
As		<2	26.9	<0.02	1.33	<0.1	0.361	<0.1	8.04	<0.1	1.25
Ba		408	2178	12.8	74.1	0.350	14.3	0.520	20.9	0.560	20.8
Pb		<1	172	<0.05	10.3	0.046	0.270	0.079	7.46	<0.1	15.8
B		<5	2,532	n.d.	n.d.	<1	11.4	<1	147	0.62	15.7
Cd		<0.1	6.76	<0.01	0.080	<0.1	<0.1	<0.1	0.210	<0.1	0.660
Cr		3.21	320	0.960	5.02	<0.1	4.71	<0.1	23.3	<0.1	2.26
Co		<1	153	<0.01	0.050	<0.1	<0.1	<0.1	3.88	<0.1	0.980
Cu		<1	451	<0.04	2.19	0.046	27.5	<0.1	32.0	0.290	29.1
Mo		5.10	90.4	<0.2	1.50	<0.1	1.44	<0.1	6.20	<0.1	1.55
Ni		<0.5	486	<0.04	2.75	<0.2	8.11	<0.1	419	<0.1	6.96
Hg		n.d.	n.d.	<0.02	<0.02	<0.01	0.023	<0.01	0.028	<0.01	0.020
Se		5.87	30.9	n.d.	n.d.	0.079	0.331	<0.1	3.19	<0.1	1.14
Tl		<1	8.44	<0.01	0.110	<0.1	<0.1	<0.1	<0.1	<0.1	<0.1
V		2.31	240	<0.7	14.5	0.358	5.55	<0.1	64.2	<0.1	2.19
Zn		<10	1423	<0.7	11.9	<1	15.7	0.578	90.0	3.02	154

n.d. = not determined.

**Table 8:** Leachate concentrations of chromium and vanadium for different mineral building materials.

Source	Examined Material	Eluate/Leachate Concentration in µg/L					
		Cr			V		
		Outdoor	Lab. Irr.	DSLT	Outdoor	Lab. Irr.	DSLT
[15,21]	reinforcement fiber plaster	<0.08–68.9	<0.5–26.4	<0.5–2.8	0.86–29.4	<0.5–8.3	<0.5–6.4
	lime-cement plaster	<0.08–141	<0.5–30.1	<0.5–5	<0.08–8.81	0.6–47.6	<0.5–5.1
	face masonry mortar	<0.08–93.2	<0.5–42.4	<0.5–7.8	<0.08–35.0	0.8–44.3	<0.5–8.4
[4]	concrete new	16–39	-	-	-	-	-
	concrete old	<1–6	-	-	-	-	-
[43]	unspecified mineral building materials	-	0.1–150	<1–49	-	<0.2–132	1–128
[44]	concrete slabs	<2–104	-	<2 *	-	-	-
[45]	ECRICEM mortar statistics	-	-	0.45–14	-	-	0.15–50
**this work**	**C^3^**	**<0.1–13.4**	**<0.1–4.71**	**0.96–5.02**	**<0.1–64.2**	**0.358–5.55**	**0.7–14.5**

* results of a multi batch test similar to the DSLT (64 days, 50 L/(m^2^ ·water renewal), eluate collection at every renewal after 2, 4, 8 h and 1, 2, 4, 9, 16, 36 and 64 days).

## Supplementary Materials

The following are available online at https://www.mdpi.com/1996-1944/13/19/4405/s1, Table S1: Laboratory irrigation results of the test specimen L 1-2 A: leachate amount, pH value, electrical conductivity and concentrations of sodium, potassium, calcium, chloride, and sulfate, Table S2: Laboratory irrigation results of the test specimen L 1-2 A: heavy metal and trace element concentrations, part 1, Table S3: Laboratory irrigation results of the test specimen L 1-2 A: heavy metal and trace element concentrations, part 2, Table S4: Laboratory irrigation results of the test specimen L 1-2 B: leachate amount, pH value, electrical conductivity and concentrations of sodium, potassium, calcium, chloride, and sulfate, Table S5: Laboratory irrigation results of the test specimen L 1-2 B: heavy metal and trace element concentrations, part 1, Table S6: Laboratory irrigation results of the test specimen L 1-2 B: heavy metal and trace element concentrations, part 2, Table S7: Laboratory irrigation results of the test specimen L 4-2 A: leachate amount, pH value, electrical conductivity and concentrations of sodium, potassium, calcium, chloride, and sulfate, Table S8: Laboratory irrigation results of the test specimen L 4-2 A: heavy metal and trace element concentrations, part 1, Table S9: Laboratory irrigation results of the test specimen L 4-2 A: heavy metal and trace element concentrations, part 2, Table S10: Laboratory irrigation results of the test specimen L 4-2 B: leachate amount, pH value, electrical conductivity and concentrations of sodium, potassium, calcium, chloride, and sulfate, Table S11: Laboratory irrigation results of the test specimen L 4-2 B: heavy metal and trace element concentrations, part 1, Table S12: Laboratory irrigation results of the test specimen L 4-2 B: heavy metal and trace element concentrations, part 2, Table S13: Laboratory irrigation results of the test specimen L 1-20 A: leachate amount, pH value, electrical conductivity and concentrations of sodium, potassium, calcium, chloride, and sulfate, Table S14: Laboratory irrigation results of the test specimen L 1-20 A: heavy metal and trace element concentrations, part 1, Table S15: Laboratory irrigation results of the test specimen L 1-20 A: heavy metal and trace element concentrations, part 2, Table S16: Laboratory irrigation results of the test specimen L 1-20 B: leachate amount, pH value, electrical conductivity and concentrations of sodium, potassium, calcium, chloride, and sulfate, Table S17: Laboratory irrigation results of the test specimen L 1-20 B: heavy metal and trace element concentrations, part 1, Table S18: Laboratory irrigation results of the test specimen L 1-20 B: heavy metal and trace element concentrations, part 2, Table S19: Laboratory irrigation results of the test specimen L 1-20 C: leachate amount, pH value, electrical conductivity and concentrations of sodium, potassium, calcium, chloride, and sulfate, Table S20: Laboratory irrigation results of the test specimen L 1-20 C: heavy metal and trace element concentrations, part 1, Table S21: Laboratory irrigation results of the test specimen L 1-20 C: heavy metal and trace element concentrations, part 2, Table S22: Laboratory irrigation results of the test specimen L 1-20 D: leachate amount, pH value, electrical conductivity and concentrations of sodium, potassium, calcium, chloride, and sulfate, Table S23: Laboratory irrigation results of the test specimen L 1-20 D: heavy metal and trace element concentrations, part 1, Table S24: Laboratory irrigation results of the test specimen L 1-20 D: heavy metal and trace element concentrations, part 2, Table S25: Outdoor irrigation results of the test specimen F BW: leachate amount, pH value, electrical conductivity and concentrations of sodium, potassium, calcium, chloride, and sulfate, Table S26: Outdoor irrigation results of the test specimen F BW: heavy metal and trace element concentrations, part 1, Table S27: Outdoor irrigation results of the test specimen F BW: heavy metal and trace element concentrations, part 2, Table S28: Outdoor irrigation results of the test specimen F 1 A: leachate amount, pH value, electrical conductivity and concentrations of sodium, potassium, calcium, chloride, and sulfate, Table S29: Outdoor irrigation results of the test specimen F 1 A: heavy metal and trace element concentrations, part 1, Table S30: Outdoor irrigation results of the test specimen F 1 A: heavy metal and trace element concentrations, part 2, Table S31: Outdoor irrigation results of the test specimen F 1 B: leachate amount, pH value, electrical conductivity and concentrations of sodium, potassium, calcium, chloride, and sulfate, Table S32: Outdoor irrigation results of the test specimen F 1 B: heavy metal and trace element concentrations, part 1, Table S33: Outdoor irrigation results of the test specimen F 1 B: heavy metal and trace element concentrations, part 2, Table S34: Outdoor irrigation results of the test specimen F 1 C: leachate amount, pH value, electrical conductivity and concentrations of sodium, potassium, calcium, chloride, and sulfate, Table S35: Outdoor irrigation results of the test specimen F 1 C: heavy metal and trace element concentrations, part 1, Table S36: Outdoor irrigation results of the test specimen F 1 C: heavy metal and trace element concentrations, part 2, Table S37: Outdoor irrigation results of the test specimen F 1g A: leachate amount, pH value, electrical conductivity and concentrations of sodium, potassium, calcium, chloride, and sulfate, Table S38: Outdoor irrigation results of the test specimen F 1g A: heavy metal and trace element concentrations, part 1, Table S39: Outdoor irrigation results of the test specimen F 1g A: heavy metal and trace element concentrations, part 2, Table S40: Outdoor irrigation results of the test specimen F 1g B: leachate amount, pH value, electrical conductivity and concentrations of sodium, potassium, calcium, chloride, and sulfate, Table S41: Outdoor irrigation results of the test specimen F 1g B: heavy metal and trace element concentrations, part 1, Table S42: Outdoor irrigation results of the test specimen F 1g B: heavy metal and trace element concentrations, part 2, Table S43: Outdoor irrigation results of the test specimen F 4g A: leachate amount, pH value, electrical conductivity, and concentrations of sodium, potassium, calcium, chloride, and sulfate, Table S44: Outdoor irrigation results of the test specimen F 4g A: heavy metal and trace element concentrations, part 1, Table S45: Outdoor irrigation results of the test specimen F 4g A: heavy metal and trace element concentrations, part 2, Table S46: Outdoor irrigation results of the test specimen F 4g B: leachate amount, pH value, electrical conductivity, and concentrations of sodium, potassium, calcium, chloride, and sulfate, Table S47: Outdoor irrigation results of the test specimen F 4g B: heavy metal and trace element concentrations, part 1, Table S48: Outdoor irrigation results of the test specimen F 4g B: heavy metal and trace element concentrations, part 2.
