# Supplementary material for: Leaching of Carbon Reinforced Concrete—Part 1: Experimental Investigations
Source: Materials (Basel). 2020 Oct 2;13(19):4405. doi: 10.3390/ma13194405 (PMC7579653; doi:10.3390/ma13194405)
Supplement: Supplementary file 1 [file materials-13-04405-s001.pdf]

materials

MDPI

Electronic Supplement

Table S1. Laboratory irrigation results of the test specimen L 1-2 A: leachate amount, pH value, electrical conductivity and concentrations of sodium, potassium, calcium, chloride, and sulfate.

Day of Testing	Leachate	Amount of Leachate L/m² *	pH -	EC µS/cm	Na mg/L	K mg/L	Ca mg/L	Cl^- mg/L	SO_4^{2-} mg/L
1	L 1-2 A1	6.32	9.92	93.3	5.0	13.5	13.2	< 0.1	4.9
2	L 1-2 A2	12.3	8.96	85.6	4.5	12.6	10.1	< 0.1	3.2
3	L 1-2 A3	37.9	8.20	62.8	2.2	5.5	17.6	< 0.1	0.3
4	L 1-2 A4	12.2	7.75	52.5	2.0	5.2	5.8	< 0.1	1.1
5	L 1-2 A5	6.79	6.89	37.25	0.6	1.2	8.0	< 0.1	1.9
8	L 1-2 A6	6.34	7.52	73.9	2.9	6.9	6.9	< 0.1	1.7
9	L 1-2 A7	11.9	7.44	74.4	1.4	3.2	16.5	< 0.1	0.7
10	L 1-2 A8	42.0	7.49	54.1	1.2	3.2	7.2	< 0.1	0.5
11	L 1-2 A9	10.8	7.56	64.2	3.3	6.9	7.3	< 0.1	0.8
12	L 1-2 A10	6.28	7.50	56.8	2.2	4.9	10.0	< 0.1	1.5
15	L 1-2 A11	5.88	7.40	58.1	1.8	4.3	7.7	< 0.1	0.5
16	L 1-2 A12	10.7	7.42	40.9	0.6	1.6	6.1	< 0.1	0.4
17	L 1-2 A13	26.5	7.36	46.9	1.3	3.2	6.5	0.3	0.5
18	L 1-2 A14	7.13	7.31	52.2	1.9	4.0	7.5	< 0.1	0.7
19	L 1-2 A15	6.41	7.24	45.2	1.3	3.0	8.0	0.2	0.8
22	L 1-2 A16	4.83	7.25	40.9	1.1	2.7	4.7	< 0.1	0.7
23	L 1-2 A17	10.9	7.34	25.7	0.2	0.9	3.8	< 0.1	0.3
24	L 1-2 A18	47.6	7.28	37.3	0.6	1.6	4.3	< 0.1	0.1
25	L 1-2 A19	12.5	7.24	40	1.3	2.4	5.3	< 0.1	0.3
26	L 1-2 A20	6.28	6.77	37.6	1.5	2.9	4.9	< 0.1	2.3
29	L 1-2 A21	6.88	7.40	112	2.9	5.2	2.4	< 0.1	0.3
30	L 1-2 A22	12.5	7.40	54	2.2	4.2	2.4	< 0.1	0.3
31	L 1-2 A23	48.1	7.30	46	1.1	2.6	2.9	< 0.1	0.2
32	L 1-2 A24	14.7	7.30	53	1.3	3.2	3.5	0.5	0.5
33	L 1-2 A25	6.44	7.40	54	2.1	5.0	4.5	0.5	1.1
36	L 1-2 A26	6.76	7.40	54	1.3	3.0	4.2	0.7	0.3
37	L 1-2 A27	12.8	7.20	35	0.8	2.5	4.2	0.6	0.2
38	L 1-2 A28	46.7	7.10	37	0.4	1.4	3.4	0.5	0.3
39	L 1-2 A29	13.1	7.30	37	1.7	4.2	4.8	0.4	0.8
40	L 1-2 A30	6.66	7.20	48	1.3	3.8	4.9	0.3	0.4

* corresponding to m² of the test specimens´ surface.

Table S2. Laboratory irrigation results of the test specimen L 1-2 A: heavy metal and trace element concentrations, part 1.

Leachate	Sb µg/l	As µg/l	Ba µg/l	Pb µg/l	B µg/l	Cd µg/l	Cr µg/l	Co µg/l
L 1-2 A1	0.218	< 0.1	7.69	< 0.1	6.25	< 0.1	1.31	< 0.1
L 1-2 A2	0.241	< 0.1	4.53	< 0.1	7.15	< 0.1	1.64	< 0.1
L 1-2 A3	0.116	< 0.1	3.19	< 0.1	4.92	< 0.1	0.685	< 0.1
L 1-2 A4	0.219	< 0.1	1.90	< 0.1	4.77	< 0.1	0.762	< 0.1
L 1-2 A5	0.168	< 0.1	1.29	< 0.1	4.11	< 0.1	0.450	< 0.1
L 1-2 A6	0.141	0.102	1.39	< 0.1	6.78	< 0.1	0.867	< 0.1

L 1-2 A7	0.100	< 0.1	1.40	< 0.1	3.94	< 0.1	0.475	< 0.1
L 1-2 A8	0.100	< 0.1	1.54	< 0.1	3.11	< 0.1	0.320	< 0.1
L 1-2 A9	0.241	< 0.1	1.76	< 0.1	4.51	< 0.1	0.535	< 0.1
L 1-2 A10	0.100	< 0.1	1.48	< 0.1	5.45	< 0.1	0.615	< 0.1
L 1-2 A11	0.100	< 0.1	1.16	< 0.1	3.40	< 0.1	0.286	< 0.1
L 1-2 A12	0.100	< 0.1	1.07	< 0.1	2.53	< 0.1	0.222	< 0.1
L 1-2 A13	0.106	< 0.1	1.42	< 0.1	3.59	< 0.1	0.371	< 0.1
L 1-2 A14	0.100	< 0.1	1.56	< 0.1	3.97	< 0.1	0.269	< 0.1
L 1-2 A15	0.100	< 0.1	1.35	< 0.1	3.93	< 0.1	0.270	< 0.1
L 1-2 A16	< 0.1	< 0.1	1.21	< 0.1	2.67	< 0.1	0.125	< 0.1
L 1-2 A17	< 0.1	< 0.1	0.99	< 0.1	1.59	< 0.1	0.100	< 0.1
L 1-2 A18	< 0.1	< 0.1	1.09	< 0.1	3.58	< 0.1	0.102	< 0.1
L 1-2 A19	< 0.1	< 0.1	1.26	< 0.1	3.74	< 0.1	0.184	< 0.1
L 1-2 A20	0.104	< 0.1	1.15	< 0.1	3.93	< 0.1	0.263	< 0.1
L 1-2 A21	< 0.1	0.107	0.77	< 0.1	3.54	< 0.1	0.232	< 0.1
L 1-2 A22	< 0.1	0.153	4.48	< 0.1	4.37	< 0.1	0.275	< 0.1
L 1-2 A23	< 0.1	0.100	3.42	< 0.1	3.00	< 0.1	0.357	< 0.1
L 1-2 A24	< 0.1	0.132	2.54	< 0.1	3.39	< 0.1	0.224	< 0.1
L 1-2 A25	< 0.1	0.222	1.98	< 0.1	5.96	< 0.1	0.357	< 0.1
L 1-2 A26	< 0.1	0.156	1.88	< 0.1	4.12	< 0.1	0.352	< 0.1
L 1-2 A27	< 0.1	0.124	1.47	< 0.1	3.12	< 0.1	0.249	< 0.1
L 1-2 A28	< 0.1	0.100	1.39	< 0.1	2.14	< 0.1	0.134	< 0.1
L 1-2 A29	< 0.1	0.159	1.47	< 0.1	4.95	< 0.1	0.411	< 0.1
L 1-2 A30	< 0.1	0.180	1.30	< 0.1	4.78	< 0.1	0.315	< 0.1

Table S3. Laboratory irrigation results of the test specimen L 1-2 A: heavy metal and trace element concentrations, part 2.

Leachate	Cu µg/l	Mo µg/l	Ni µg/l	Hg µg/l	Se µg/l	Tl µg/l	V µg/l	Zn µg/l
L 1-2 A1	0.312	0.729	1.58	< 0.01	0.287	< 0.1	2.97	< 1
L 1-2 A2	0.245	0.636	1.21	< 0.01	0.183	< 0.1	3.64	1.11
L 1-2 A3	0.321	0.440	2.19	< 0.01	0.100	< 0.1	1.64	1.02
L 1-2 A4	1.760	0.329	2.18	< 0.01	0.100	< 0.1	1.65	9.83
L 1-2 A5	1.765	0.185	2.49	< 0.01	0.079	< 0.1	0.985	2.00
L 1-2 A6	0.350	0.362	1.50	< 0.01	< 0.1	< 0.1	2.46	3.75
L 1-2 A7	0.397	0.198	1.32	< 0.01	< 0.1	< 0.1	1.48	1.21
L 1-2 A8	0.587	0.144	1.33	< 0.01	< 0.1	< 0.1	1.01	1.69
L 1-2 A9	0.642	0.189	1.29	< 0.01	< 0.1	< 0.1	1.55	2.27
L 1-2 A10	0.639	0.203	1.28	< 0.01	< 0.1	< 0.1	1.67	2.98
L 1-2 A11	0.472	0.122	1.22	< 0.01	< 0.1	< 0.1	1.07	1.47
L 1-2 A12	0.707	0.100	1.29	< 0.01	< 0.1	< 0.1	0.797	7.90
L 1-2 A13	1.700	0.125	1.11	< 0.01	< 0.1	< 0.1	1.19	1.89
L 1-2 A14	0.489	0.100	0.950	< 0.01	< 0.1	< 0.1	1.20	1.50
L 1-2 A15	0.419	0.100	0.935	< 0.01	< 0.1	< 0.1	1.02	1.99
L 1-2 A16	0.464	0.100	1.06	< 0.01	< 0.1	< 0.1	0.760	4.09
L 1-2 A17	0.163	0.100	1.16	< 0.01	< 0.1	< 0.1	0.364	1.82
L 1-2 A18	0.252	0.284	2.61	< 0.01	< 0.1	< 0.1	0.601	2.15
L 1-2 A19	0.355	0.206	2.03	< 0.01	< 0.1	< 0.1	0.864	3.41
L 1-2 A20	0.569	0.191	1.68	< 0.01	< 0.1	< 0.1	0.997	1.42
L 1-2 A21	0.254	0.263	0.254	< 0.01	< 0.1	< 0.1	0.775	0.554
L 1-2 A22	0.264	0.217	0.642	< 0.01	< 0.1	< 0.1	1.16	1.71
L 1-2 A23	0.233	0.142	0.816	< 0.01	< 0.1	< 0.1	0.684	0.725

L 1-2 A24	0.360	0.121	0.327	< 0.01	< 0.1	< 0.1	0.912	1.44
L 1-2 A25	0.329	0.240	0.224	< 0.01	< 0.1	< 0.1	1.86	0.570
L 1-2 A26	0.412	0.136	0.224	< 0.01	< 0.1	< 0.1	1.07	1.84
L 1-2 A27	0.300	0.100	0.207	< 0.01	< 0.1	< 0.1	0.914	1.06
L 1-2 A28	0.371	0.100	0.188	< 0.01	< 0.1	< 0.1	0.650	1.07
L 1-2 A29	1.170	0.133	0.361	< 0.01	< 0.1	< 0.1	1.74	1.06
L 1-2 A30	0.538	0.100	0.253	< 0.01	< 0.1	< 0.1	1.39	1.31

Table S4. Laboratory irrigation results of the test specimen L 1-2 B: leachate amount, pH value, electrical conductivity and concentrations of sodium, potassium, calcium, chloride, and sulfate.

Day of Testing	Leachate	Amount of Leachate L/m² *	pH	EC	Na	K	Ca	Cl^-	SO_4^{2-}
			-	µS/cm	mg/L	mg/L	mg/L	mg/L	mg/L
1	L 1-2 B1	6.31	9.01	103	2.3	5.9	17.4	0.2	2.9
2	L 1-2 B2	11.9	8.48	75.0	1.6	3.4	10.4	< 0.1	1.7
3	L 1-2 B3	35.4	8.13	51.0	1.1	3.1	5.0	< 0.1	1.1
4	L 1-2 B4	12.1	7.86	64.5	2.0	5.2	5.8	< 0.1	1.8
5	L 1-2 B5	7.56	7.68	68.1	2.2	5.1	8.4	< 0.1	2.1
8	L 1-2 B6	6.72	7.56	52.4	0.7	2.2	5.3	< 0.1	0.8
9	L 1-2 B7	12.9	7.49	46.9	0.6	1.7	8.3	0.2	0.6
10	L 1-2 B8	34.2	7.30	39.4	0.5	1.5	4.8	0.2	0.6
11	L 1-2 B9	12.2	7.39	34.3	0.7	1.9	4.7	< 0.1	0.5
12	L 1-2 B10	6.98	7.37	49.7	1.1	2.5	5.5	< 0.1	1.3
15	L 1-2 B11	5.99	7.37	43.0	0.7	1.8	5.5	0.2	0.4
16	L 1-2 B12	11.3	7.32	59.8	0.6	1.2	5.9	< 0.1	0.5
17	L 1-2 B13	45.5	7.37	30.9	0.6	1.4	5.1	< 0.1	0.6
18	L 1-2 B14	9.91	7.24	39.8	1.0	1.8	11.2	< 0.1	0.5
19	L 1-2 B15	5.91	7.23	49.3	0.6	1.4	4.6	< 0.1	0.5
22	L 1-2 B16	7.18	7.28	40.0	1.0	2.4	6.2	0.2	0.6
23	L 1-2 B17	12.5	7.21	31.5	0.4	1.0	4.6	< 0.1	0.2
24	L 1-2 B18	46.7	7.28	27.3	1.4	1.1	6.2	< 0.1	0.2
25	L 1-2 B19	12.5	7.26	27.5	0.4	1.0	4.2	0.2	0.3
26	L 1-2 B20	6.3	7.14	31.9	6.2	2.4	15.4	0.2	3.4
29	L 1-2 B21	8.46	7.50	50.0	4.6	7.8	3.4	< 0.1	1.3
30	L 1-2 B22	12.1	7.40	43.0	2.1	4.7	3.1	< 0.1	0.2
31	L 1-2 B23	47.4	7.30	32.0	1.1	3.0	3.2	< 0.1	0.4
32	L 1-2 B24	13.1	7.30	46.0	1.8	4.3	4.0	1.0	0.3
33	L 1-2 B25	6.73	7.30	41.9	1.6	3.9	3.8	1.0	0.2
36	L 1-2 B26	9.05	7.30	23.24	1.2	3.3	3.9	0.5	0.5
37	L 1-2 B27	13.5	7.30	41.1	0.8	2.6	4.1	0.7	0.3
38	L 1-2 B28	35.5	7.20	33.7	0.3	1.1	3.8	0.4	0.2
39	L 1-2 B29	19.0	7.20	28.5	0.4	1.2	3.6	0.4	0.3
40	L 1-2 B30	6.74	7.20	23.9	0.3	1.5	3.7	0.1	0.2

* corresponding to m² of the test specimens´ surface.

Table S5. Laboratory irrigation results of the test specimen L 1-2 B: heavy metal and trace element concentrations, part 1.

Leachate	Sb µg/l	As µg/l	Ba µg/l	Pb µg/l	B µg/l	Cd µg/l	Cr µg/l	Co µg/l
L 1-2 B1	0.144	< 0.1	5.90	< 0.1	5.01	< 0.1	0.854	< 0.1
L 1-2 B2	0.133	< 0.1	4.64	< 0.1	6.17	< 0.1	1.16	< 0.1
L 1-2 B3	< 0.1	< 0.1	2.19	< 0.1	3.70	< 0.1	0.640	< 0.1

L 1-2 B4	< 0.1	0.1020	1.74	< 0.1	5.18	< 0.1	0.926	< 0.1
L 1-2 B5	< 0.1	0.1250	1.66	< 0.1	6.62	< 0.1	1.280	< 0.1
L 1-2 B6	< 0.1	< 0.1	1.22	0.117	4.48	< 0.1	0.697	< 0.1
L 1-2 B7	< 0.1	< 0.1	1.86	0.164	3.76	< 0.1	0.569	< 0.1
L 1-2 B8	< 0.1	< 0.1	1.22	0.046	2.75	< 0.1	0.409	< 0.1
L 1-2 B9	< 0.1	< 0.1	1.13	< 0.1	3.60	< 0.1	0.289	< 0.1
L 1-2 B10	< 0.1	< 0.1	1.39	< 0.1	4.46	< 0.1	0.471	< 0.1
L 1-2 B11	< 0.1	< 0.1	0.95	0.112	2.54	< 0.1	0.221	< 0.1
L 1-2 B12	< 0.1	< 0.1	0.99	< 0.1	2.34	< 0.1	0.145	< 0.1
L 1-2 B13	< 0.1	< 0.1	0.94	< 0.1	2.30	< 0.1	0.171	< 0.1
L 1-2 B14	< 0.1	< 0.1	1.19	< 0.1	3.02	< 0.1	0.234	< 0.1
L 1-2 B15	< 0.1	< 0.1	1.04	< 0.1	2.48	< 0.1	0.191	< 0.1
L 1-2 B16	< 0.1	< 0.1	1.14	< 0.1	2.57	< 0.1	0.172	< 0.1
L 1-2 B17	< 0.1	< 0.1	0.81	< 0.1	1.66	< 0.1	< 0.1	< 0.1
L 1-2 B18	< 0.1	< 0.1	1.13	< 0.1	1.83	< 0.1	0.113	< 0.1
L 1-2 B19	< 0.1	< 0.1	1.11	< 0.1	1.88	< 0.1	0.150	< 0.1
L 1-2 B20	< 0.1	< 0.1	1.09	< 0.1	2.43	< 0.1	0.211	< 0.1
L 1-2 B21	< 0.1	0.200	1.12	< 0.1	5.32	< 0.1	0.477	< 0.1
L 1-2 B22	< 0.1	0.130	1.07	< 0.1	2.08	< 0.1	0.194	< 0.1
L 1-2 B23	< 0.1	< 0.1	1.66	< 0.1	1.33	< 0.1	0.124	< 0.1
L 1-2 B24	< 0.1	0.129	3.13	< 0.1	2.81	< 0.1	0.340	< 0.1
L 1-2 B25	< 0.1	0.148	2.02	< 0.1	4.69	< 0.1	0.377	< 0.1
L 1-2 B26	< 0.1	0.150	1.49	< 0.1	3.57	< 0.1	0.315	< 0.1
L 1-2 B27	< 0.1	0.131	1.60	< 0.1	2.87	< 0.1	0.232	< 0.1
L 1-2 B28	< 0.1	< 0.1	1.48	< 0.1	1.80	< 0.1	0.124	< 0.1
L 1-2 B29	< 0.1	< 0.1	1.42	< 0.1	1.77	< 0.1	0.132	< 0.1
L 1-2 B30	n. d.	n. d.	n. d.	n. d.	n. d.	n. d.	n. d.	n. d.

n. d. = not determined.

Table S6. Laboratory irrigation results of the test specimen L 1-2 B: heavy metal and trace element concentrations, part 2.

Leachate	Cu µg/l	Mo µg/l	Ni µg/l	Hg µg/l	Se µg/l	Tl µg/l	V µg/l	Zn µg/l
L 1-2 B1	0.897	0.758	4.23	0.0184	0.129	< 0.1	1.50	2.13
L 1-2 B2	0.826	0.296	2.43	0.0208	< 0.1	< 0.1	2.17	1.14
L 1-2 B3	0.866	0.154	2.41	0.0188	< 0.1	< 0.1	1.05	1.56
L 1-2 B4	1.37	0.219	2.74	0.0181	< 0.1	< 0.1	1.80	5.07
L 1-2 B5	0.986	0.267	2.42	0.0197	< 0.1	< 0.1	2.57	1.54
L 1-2 B6	0.974	0.109	2.71	0.0234	< 0.1	< 0.1	1.31	2.31
L 1-2 B7	1.43	< 0.1	3.10	0.0193	< 0.1	< 0.1	1.09	5.68
L 1-2 B8	0.866	< 0.1	3.21	0.0138	< 0.1	< 0.1	0.743	1.96
L 1-2 B9	0.428	0.217	4.84	< 0.01	< 0.1	< 0.1	0.885	1.86
L 1-2 B10	0.859	0.228	3.52	< 0.01	< 0.1	< 0.1	1.17	5.37
L 1-2 B11	1.01	0.129	2.92	< 0.01	< 0.1	< 0.1	0.683	3.20
L 1-2 B12	0.918	< 0.1	2.60	< 0.01	< 0.1	< 0.1	0.569	3.16
L 1-2 B13	0.464	< 0.1	2.48	< 0.01	< 0.1	< 0.1	0.665	3.24
L 1-2 B14	0.491	< 0.1	2.32	< 0.01	< 0.1	< 0.1	0.878	3.26
L 1-2 B15	0.813	< 0.1	2.14	< 0.01	< 0.1	< 0.1	0.657	2.03
L 1-2 B16	0.759	< 0.1	2.24	< 0.01	< 0.1	< 0.1	0.792	2.63
L 1-2 B17	0.312	< 0.1	2.05	< 0.01	< 0.1	< 0.1	0.467	1.59
L 1-2 B18	0.325	< 0.1	2.06	< 0.01	< 0.1	< 0.1	0.529	1.68
L 1-2 B19	0.418	< 0.1	2.07	< 0.01	< 0.1	< 0.1	0.575	3.03

L 1-2 B20	0.399	< 0.1	2.06	< 0.01	< 0.1	< 0.1	0.679	8.93
L 1-2 B21	0.510	0.181	0.221	< 0.01	< 0.1	< 0.1	1.62	0.696
L 1-2 B22	0.628	< 0.1	0.321	< 0.01	< 0.1	< 0.1	0.823	1.77
L 1-2 B23	0.549	< 0.1	0.226	< 0.01	< 0.1	< 0.1	0.597	1.03
L 1-2 B24	0.380	< 0.1	0.222	< 0.01	< 0.1	< 0.1	1.07	1.60
L 1-2 B25	0.472	0.269	1.84	< 0.01	< 0.1	< 0.1	1.13	1.20
L 1-2 B26	0.379	0.177	0.207	< 0.01	< 0.1	< 0.1	1.02	2.46
L 1-2 B27	0.446	0.118	0.242	< 0.01	< 0.1	< 0.1	0.828	< 1
L 1-2 B28	1.19	< 0.1	0.381	< 0.01	< 0.1	< 0.1	0.575	3.11
L 1-2 B29	0.879	< 0.1	0.336	< 0.01	< 0.1	< 0.1	0.647	2.63
L 1-2 B30	n. d.	n. d.	n. d.	n. d.	n. d.	n. d.	n. d.	n. d.

n. d. = not determined.

Table S7. Laboratory irrigation results of the test specimen L 4-2 A: leachate amount, pH value, electrical conductivity and concentrations of sodium, potassium, calcium, chloride, and sulfate.

Day of Testing	Leachate	Amount of Leachate	pH	EC	Na	K	Ca	Cl^-	SO_4^{2-}
		L/m² *	-	µS/cm	mg/L	mg/L	mg/L	mg/L	mg/L
1	L 4-2 A1	6.56	10.3	100	5.4	12.4	3.5	0.4	7.1
2	L 4-2 A2	14.2	10.3	120	6.6	17.3	1.9	< 0.1	3.3
3	L 4-2 A3	28.7	9.95	71.5	2.4	6.1	3.8	< 0.1	1.4
4	L 4-2 A4	10.9	9.22	70.0	2.9	6.6	8.0	0.1	1.6
5	L 4-2 A5	6.34	10.2	132	9.1	23.8	1.7	0.2	4.3
8	L 4-2 A6	6.38	9.23	98.8	6.8	16.1	6.1	< 0.1	4.3
9	L 4-2 A7	12.6	8.90	49.2	1.5	3.6	6.3	< 0.1	0.9
10	L 4-2 A8	40.2	9.31	48.2	1.2	2.7	6.7	< 0.1	0.9
11	L 4-2 A9	12.7	8.67	44.0	1.2	2.8	6.2	< 0.1	0.8
12	L 4-2 A10	6.48	8.63	58.5	2.6	5.5	6.4	0.2	1.2
15	L 4-2 A11	7.43	8.80	76.0	4.6	9.8	6.2	< 0.1	0.6
16	L 4-2 A12	12.4	8.94	49.4	2.2	5.2	5.1	0.2	1.7
17	L 4-2 A13	43.2	7.94	78.3	0.7	1.4	6.0	< 0.1	0.4
18	L 4-2 A14	29.4	7.84	41.8	0.9	1.8	6.8	< 0.1	0.4
19	L 4-2 A15	5.95	7.83	62.0	2.2	4.7	8.3	< 0.1	0.7
22	L 4-2 A16	8.09	8.47	43.2	1.7	3.6	5.5	< 0.1	0.7
23	L 4-2 A17	12.4	7.88	48.6	1.9	5.1	5.5	< 0.1	0.3
24	L 4-2 A18	50.5	8.25	24.4	0.4	1.0	3.6	< 0.1	0.1
25	L 4-2 A19	12.5	8.00	38.7	0.8	1.9	5.8	< 0.1	0.4
26	L 4-2 A20	6.36	7.91	42.2	1.3	3.1	5.6	< 0.1	0.5
29	L 4-2 A21	6.34	n. d.	n. d.	n. d.	n. d.	n. d.	n. d.	n. d.
30	L 4-2 A22	12.5	n. d.	n. d.	n. d.	n. d.	n. d.	n. d.	n. d.
31	L 4-2 A23	47.5	n. d.	n. d.	n. d.	n. d.	n. d.	n. d.	n. d.
32	L 4-2 A24	16.3	n. d.	n. d.	n. d.	n. d.	n. d.	n. d.	n. d.
33	L 4-2 A25	8.53	n. d.	n. d.	n. d.	n. d.	n. d.	n. d.	n. d.
36	L 4-2 A26	6.59	n. d.	n. d.	n. d.	n. d.	n. d.	n. d.	n. d.
37	L 4-2 A27	12.6	n. d.	n. d.	n. d.	n. d.	n. d.	n. d.	n. d.
38	L 4-2 A28	49.6	n. d.	n. d.	n. d.	n. d.	n. d.	n. d.	n. d.
39	L 4-2 A29	12.7	n. d.	n. d.	n. d.	n. d.	n. d.	n. d.	n. d.
40	L 4-2 A30	7.50	n. d.	n. d.	n. d.	n. d.	n. d.	n. d.	n. d.

* corresponding to m² of the test specimens´ surface; n. d. = not detemined.

Table S8. Laboratory irrigation results of the test specimen L 4-2 A: heavy metal and trace element concentrations, part 1.

Leachate	Sb µg/l	As µg/l	Ba µg/l	Pb µg/l	B µg/l	Cd µg/l	Cr µg/l	Co µg/l
L 4-2 A1	0.293	< 0.1	10.3	< 0.1	4.85	< 0.1	0.571	< 0.1
L 4-2 A2	0.181	< 0.1	5.01	< 0.1	4.85	< 0.1	0.949	< 0.1
L 4-2 A3	0.128	< 0.1	4.35	< 0.1	3.49	< 0.1	0.718	< 0.1
L 4-2 A4	0.141	< 0.1	3.03	< 0.1	4.34	< 0.1	0.660	< 0.1
L 4-2 A5	0.308	0.177	2.60	< 0.1	7.99	< 0.1	1.87	< 0.1
L 4-2 A6	0.356	0.309	1.63	< 0.1	10.6	< 0.1	1.70	< 0.1
L 4-2 A7	0.110	< 0.1	1.34	< 0.1	3.34	< 0.1	0.309	< 0.1
L 4-2 A8	0.101	< 0.1	1.55	< 0.1	3.38	< 0.1	0.259	< 0.1
L 4-2 A9	< 0.1	< 0.1	1.70	< 0.1	2.84	< 0.1	0.260	< 0.1
L 4-2 A10	0.151	< 0.1	2.40	< 0.1	5.00	< 0.1	0.390	< 0.1
L 4-2 A11	0.149	< 0.1	2.02	< 0.1	7.13	< 0.1	0.693	< 0.1
L 4-2 A12	< 0.1	< 0.1	1.41	< 0.1	4.54	< 0.1	0.341	< 0.1
L 4-2 A13	< 0.1	< 0.1	1.40	< 0.1	2.90	< 0.1	0.158	< 0.1
L 4-2 A14	0.173	0.131	2.50	< 0.1	4.67	< 0.1	0.399	< 0.1
L 4-2 A15	< 0.1	< 0.1	1.31	< 0.1	1.99	< 0.1	0.281	< 0.1
L 4-2 A16	< 0.1	< 0.1	1.53	< 0.1	2.82	< 0.1	0.249	< 0.1
L 4-2 A17	< 0.1	< 0.1	1.22	< 0.1	< 1	< 0.1	< 0.1	< 0.1
L 4-2 A18	< 0.1	< 0.1	1.22	< 0.1	< 1	< 0.1	< 0.1	< 0.1
L 4-2 A19	< 0.1	< 0.1	1.75	< 0.1	1.78	< 0.1	0.319	< 0.1
L 4-2 A20	< 0.1	< 0.1	1.76	0.113	1.93	< 0.1	0.177	< 0.1
L 4-2 A21	< 0.1	0.297	0.949	< 0.1	3.54	< 0.1	0.349	< 0.1
L 4-2 A22	< 0.1	0.169	0.550	< 0.1	2.21	< 0.1	0.152	< 0.1
L 4-2 A23	< 0.1	0.105	0.493	< 0.1	1.55	< 0.1	0.103	< 0.1
L 4-2 A24	< 0.1	< 0.1	4.21	< 0.1	1.05	< 0.1	0.100	< 0.1
L 4-2 A25	< 0.1	0.129	0.724	< 0.1	6.52	< 0.1	0.100	< 0.1
L 4-2 A26	< 0.1	0.123	0.544	< 0.1	4.22	< 0.1	0.114	< 0.1
L 4-2 A27	< 0.1	0.119	0.577	< 0.1	3.06	< 0.1	0.120	< 0.1
L 4-2 A28	< 0.1	0.196	0.563	< 0.1	4.06	< 0.1	0.182	< 0.1
L 4-2 A29	< 0.1	0.177	0.585	< 0.1	3.67	< 0.1	0.183	< 0.1
L 4-2 A30	< 0.1	0.111	0.594	< 0.1	2.19	< 0.1	0.101	< 0.1

Table S9. Laboratory irrigation results of the test specimen L 4-2 A: heavy metal and trace element concentrations, part 2.

Leachate	Cu µg/l	Mo µg/l	Ni µg/l	Hg µg/l	Se µg/l	Tl µg/l	V µg/l	Zn µg/l
L 4-2 A1	0.248	0.755	0.734	< 0.01	0.146	< 0.1	2.29	4.48
L 4-2 A2	0.127	0.483	0.698	< 0.01	0.138	< 0.1	2.68	< 1
L 4-2 A3	< 0.1	0.240	0.601	< 0.01	< 0.1	< 0.1	1.94	1.67
L 4-2 A4	0.046	0.266	0.567	< 0.01	< 0.1	< 0.1	2.14	< 1
L 4-2 A5	0.504	0.584	0.755	< 0.01	0.183	< 0.1	4.06	< 1
L 4-2 A6	0.712	0.539	0.789	< 0.01	0.222	< 0.1	4.91	< 1
L 4-2 A7	0.367	0.271	0.276	< 0.01	< 0.1	< 0.1	1.19	1.11
L 4-2 A8	0.492	0.211	0.373	< 0.01	< 0.1	< 0.1	1.03	< 1
L 4-2 A9	0.359	0.172	0.264	< 0.01	< 0.1	< 0.1	1.02	< 1
L 4-2 A10	0.570	0.297	0.264	< 0.01	< 0.1	< 0.1	1.74	2.22
L 4-2 A11	0.640	0.283	0.351	< 0.01	0.101	< 0.1	3.04	< 1
L 4-2 A12	0.830	0.147	0.364	< 0.01	< 0.1	< 0.1	1.67	1.08
L 4-2 A13	0.279	0.273	0.245	< 0.01	< 0.1	< 0.1	0.702	< 1
L 4-2 A14	1.480	0.170	0.754	< 0.01	< 0.1	< 0.1	1.80	6.08
L 4-2 A15	< 0.1	0.120	1.13	< 0.01	< 0.1	< 0.1	0.952	1.78

L 4-2 A16	< 0.1	< 0.1	2.19	< 0.01	< 0.1	< 0.1	1.31	< 1
L 4-2 A17	< 0.1	< 0.1	0.551	< 0.01	< 0.1	< 0.1	0.358	2.33
L 4-2 A18	< 0.1	< 0.1	0.655	< 0.01	< 0.1	< 0.1	0.358	< 1
L 4-2 A19	< 0.1	< 0.1	0.611	< 0.01	< 0.1	< 0.1	0.918	1.18
L 4-2 A20	0.105	< 0.1	0.682	< 0.01	< 0.1	< 0.1	0.808	4.13
L 4-2 A21	0.282	0.186	0.231	< 0.01	< 0.1	< 0.1	2.06	36.0
L 4-2 A22	0.143	< 0.1	0.154	< 0.01	< 0.1	< 0.1	1.10	3.46
L 4-2 A23	0.178	< 0.1	0.167	< 0.01	< 0.1	< 0.1	0.784	1.68
L 4-2 A24	0.102	< 0.1	0.163	< 0.01	< 0.1	< 0.1	0.513	4.24
L 4-2 A25	0.197	0.485	0.192	< 0.01	< 0.1	< 0.1	0.626	1.97
L 4-2 A26	0.174	0.287	0.242	< 0.01	< 0.1	< 0.1	0.744	3.37
L 4-2 A27	0.169	0.214	2.360	< 0.01	< 0.1	< 0.1	0.755	3.26
L 4-2 A28	0.241	0.189	0.180	< 0.01	< 0.1	< 0.1	1.37	1.59
L 4-2 A29	0.251	0.147	0.152	< 0.01	< 0.1	< 0.1	1.38	1.67
L 4-2 A30	0.348	< 0.1	0.841	< 0.01	< 0.1	< 0.1	0.689	3.91

Table S10. Laboratory irrigation results of the test specimen L 4-2 B: leachate amount, pH value, electrical conductivity and concentrations of sodium, potassium, calcium, chloride, and sulfate.

Day of Testing	Leachate	Amount of Leachate	pH	EC	Na	K	Ca	Cl^-	SO_4^{2-}
		L/m² *	-	µS/cm	mg/L	mg/L	mg/L	mg/L	mg/L
1	L 4-2 B1	6.28	10.24	107	4.8	11.7	3.3	0.2	9.0
2	L 4-2 B2	14.8	10.10	82.8	2.3	5.7	4.7	< 0.1	3.6
3	L 4-2 B3	35.2	9.85	62.9	1.1	2.8	5.6	< 0.1	1.9
4	L 4-2 B4	12.5	9.40	61.9	1.3	3.1	8.5	< 0.1	1.5
5	L 4-2 B5	6.43	9.63	74.1	2.3	5.9	6.7	< 0.1	1.7
8	L 4-2 B6	6.84	8.40	74.2	3.9	8.4	6.8	< 0.1	3.6
9	L 4-2 B7	12.9	8.66	44.2	1.3	3.3	5.2	< 0.1	1.2
10	L 4-2 B8	47.1	9.16	39.5	0.7	1.6	5.8	< 0.1	0.8
11	L 4-2 B9	12.9	8.34	43.3	1.0	2.3	6.1	< 0.1	0.8
12	L 4-2 B10	6.34	8.06	49.7	1.4	3.2	6.5	< 0.1	1.0
15	L 4-2 B11	7.63	7.86	52.8	1.7	4.0	6.2	0.2	1.1
16	L 4-2 B12	12.4	8.21	96.4	1.0	2.6	5.5	< 0.1	0.5
17	L 4-2 B13	52.6	9.01	60.5	1.0	2.5	5.7	< 0.1	0.7
18	L 4-2 B14	12.5	7.71	37.4	0.6	1.5	5.9	< 0.1	0.4
19	L 4-2 B15	6.52	7.72	47.0	1.3	3.0	6.5	< 0.1	0.6
22	L 4-2 B16	6.26	8.13	47.3	1.3	2.9	6.9	< 0.1	0.7
23	L 4-2 B17	13.1	7.58	38.1	1.2	2.6	5.2	< 0.1	0.5
24	L 4-2 B18	46.2	7.65	28.3	0.4	0.8	4.5	< 0.1	0.3
25	L 4-2 B19	12.5	7.38	33.6	0.5	1.0	5.4	< 0.1	0.3
26	L 4-2 B20	6.33	7.56	42.7	1.7	3.3	5.2	< 0.1	0.5
29	L 4-2 B21	6.43	n. d.	n. d.	n. d.	n. d.	n. d.	n. d.	n. d.
30	L 4-2 B22	13.7	n. d.	n. d.	n. d.	n. d.	n. d.	n. d.	n. d.
31	L 4-2 B23	47.5	n. d.	n. d.	n. d.	n. d.	n. d.	n. d.	n. d.
32	L 4-2 B24	13.6	n. d.	n. d.	n. d.	n. d.	n. d.	n. d.	n. d.
33	L 4-2 B25	6.18	n. d.	n. d.	n. d.	n. d.	n. d.	n. d.	n. d.
36	L 4-2 B26	6.5	n. d.	n. d.	n. d.	n. d.	n. d.	n. d.	n. d.
37	L 4-2 B27	12.5	n. d.	n. d.	n. d.	n. d.	n. d.	n. d.	n. d.
38	L 4-2 B28	47.8	n. d.	n. d.	n. d.	n. d.	n. d.	n. d.	n. d.
39	L 4-2 B29	10.1	n. d.	n. d.	n. d.	n. d.	n. d.	n. d.	n. d.
40	L 4-2 B30	6.93	n. d.	n. d.	n. d.	n. d.	n. d.	n. d.	n. d.

* corresponding to m² of the test specimens´ surface; n. d. = not determined.

Table S11. Laboratory irrigation results of the test specimen L 4-2 B: heavy metal and trace element concentrations, part 1.

Leachate	Sb µg/l	As µg/l	Ba µg/l	Pb µg/l	B µg/l	Cd µg/l	Cr µg/l	Co µg/l
L 4-2 B1	0.257	< 0.1	9.95	0.107	6.47	< 0.1	1.510	< 0.1
L 4-2 B2	0.151	< 0.1	4.92	< 0.1	6.39	< 0.1	1.40	< 0.1
L 4-2 B3	0.101	< 0.1	3.56	< 0.1	4.32	< 0.1	0.917	< 0.1
L 4-2 B4	0.113	< 0.1	2.76	< 0.1	5.15	< 0.1	0.908	< 0.1
L 4-2 B5	0.177	< 0.1	3.18	< 0.1	6.76	< 0.1	1.050	< 0.1
L 4-2 B6	0.147	0.202	1.91	< 0.1	10.0	< 0.1	1.42	< 0.1
L 4-2 B7	< 0.1	< 0.1	1.22	< 0.1	4.25	< 0.1	0.428	< 0.1
L 4-2 B8	< 0.1	< 0.1	1.20	< 0.1	2.82	< 0.1	0.245	< 0.1
L 4-2 B9	< 0.1	< 0.1	1.46	< 0.1	3.31	< 0.1	0.344	< 0.1
L 4-2 B10	< 0.1	< 0.1	2.01	< 0.1	4.16	< 0.1	0.436	< 0.1
L 4-2 B11	< 0.1	< 0.1	1.60	< 0.1	4.01	< 0.1	0.397	< 0.1
L 4-2 B12	< 0.1	< 0.1	1.32	< 0.1	2.69	< 0.1	0.224	< 0.1
L 4-2 B13	< 0.1	< 0.1	1.34	< 0.1	2.51	< 0.1	0.210	< 0.1
L 4-2 B14	< 0.1	< 0.1	1.63	< 0.1	1.13	< 0.1	0.426	< 0.1
L 4-2 B15	< 0.1	< 0.1	1.57	< 0.1	1.68	< 0.1	0.233	< 0.1
L 4-2 B16	< 0.1	< 0.1	1.67	< 0.1	2.40	< 0.1	0.301	< 0.1
L 4-2 B17	< 0.1	< 0.1	1.39	< 0.1	1.49	< 0.1	0.154	< 0.1
L 4-2 B18	< 0.1	< 0.1	1.20	< 0.1	< 1	< 0.1	< 0.1	< 0.1
L 4-2 B19	< 0.1	< 0.1	1.66	< 0.1	2.22	< 0.1	0.162	< 0.1
L 4-2 B20	< 0.1	< 0.1	2.00	< 0.1	3.63	< 0.1	0.265	< 0.1
L 4-2 B21	0.479	0.338	1.08	0.221	5.96	< 0.1	0.800	< 0.1
L 4-2 B22	0.160	0.197	0.65	< 0.1	7.90	< 0.1	0.443	< 0.1
L 4-2 B23	< 0.1	0.097	0.46	< 0.1	2.52	< 0.1	0.117	< 0.1
L 4-2 B24	< 0.1	0.102	0.52	< 0.1	2.15	< 0.1	0.160	< 0.1
L 4-2 B25	< 0.1	0.168	0.62	< 0.1	3.97	< 0.1	0.302	< 0.1
L 4-2 B26	< 0.1	0.142	0.65	< 0.1	3.75	< 0.1	0.225	< 0.1
L 4-2 B27	< 0.1	0.135	0.79	< 0.1	3.27	< 0.1	0.233	< 0.1
L 4-2 B28	< 0.1	0.109	0.58	< 0.1	2.28	< 0.1	0.171	< 0.1
L 4-2 B29	< 0.1	0.112	0.56	< 0.1	2.05	< 0.1	0.191	< 0.1
L 4-2 B30	< 0.1	< 0.1	0.59	< 0.1	1.95	< 0.1	0.277	< 0.1

Table S12. Laboratory irrigation results of the test specimen L 4-2 B: heavy metal and trace element concentrations, part 2.

Leachate	Cu µg/l	Mo µg/l	Ni µg/l	Hg µg/l	Se µg/l	Tl µg/l	V µg/l	Zn µg/l
L 4-2 B1	1.55	0.858	0.627	< 0.01	0.302	< 0.1	3.40	3.05
L 4-2 B2	0.146	0.479	0.573	< 0.01	0.239	< 0.1	3.77	2.82
L 4-2 B3	0.124	0.272	0.534	< 0.01	0.111	< 0.1	2.41	< 1
L 4-2 B4	< 0.1	0.443	0.517	< 0.01	0.100	< 0.1	2.21	< 1
L 4-2 B5	0.494	0.395	0.702	< 0.01	0.132	< 0.1	3.02	< 1
L 4-2 B6	0.278	0.533	0.822	< 0.01	0.195	< 0.1	3.80	< 1
L 4-2 B7	0.370	1.10	0.280	< 0.01	0.133	< 0.1	1.66	3.59
L 4-2 B8	1.30	0.172	0.291	< 0.01	< 0.1	< 0.1	0.964	< 1
L 4-2 B9	0.231	0.166	0.240	< 0.01	< 0.1	< 0.1	1.21	< 1
L 4-2 B10	0.443	0.183	0.281	< 0.01	< 0.1	< 0.1	1.40	1.72
L 4-2 B11	0.480	0.452	0.326	< 0.01	< 0.1	< 0.1	1.46	3.15

L 4-2 B12	0.382	0.118	0.282	< 0.01	< 0.1	< 0.1	0.909	< 1
L 4-2 B13	0.117	0.117	0.391	< 0.01	< 0.1	< 0.1	0.833	< 1
L 4-2 B14	< 0.1	0.107	0.815	< 0.01	< 0.1	< 0.1	0.711	3.57
L 4-2 B15	< 0.1	< 0.1	0.620	< 0.01	< 0.1	< 0.1	0.974	9.57
L 4-2 B16	< 0.1	< 0.1	0.627	< 0.01	< 0.1	< 0.1	0.995	2.51
L 4-2 B17	1.04	< 0.1	0.978	< 0.01	< 0.1	< 0.1	0.785	2.33
L 4-2 B18	< 0.1	< 0.1	0.549	< 0.01	< 0.1	< 0.1	0.415	< 1
L 4-2 B19	< 0.1	0.182	0.532	< 0.01	< 0.1	< 0.1	0.683	1.10
L 4-2 B20	< 0.1	0.229	0.673	< 0.01	< 0.1	< 0.1	1.27	1.82
L 4-2 B21	0.582	0.279	0.337	< 0.01	< 0.1	< 0.1	2.70	**43.4***
L 4-2 B22	0.310	0.332	0.228	< 0.01	< 0.1	< 0.1	1.82	4.71
L 4-2 B23	0.147	0.176	0.113	< 0.01	< 0.1	< 0.1	0.743	1.51
L 4-2 B24	0.189	0.146	0.154	< 0.01	< 0.1	< 0.1	0.770	5.19
L 4-2 B25	0.375	0.166	0.243	< 0.01	< 0.1	< 0.1	1.49	2.38
L 4-2 B26	0.686	0.134	0.140	< 0.01	< 0.1	< 0.1	1.22	2.04
L 4-2 B27	0.194	0.103	0.168	< 0.01	< 0.1	< 0.1	1.24	2.12
L 4-2 B28	< 0.2	< 0.1	0.161	< 0.01	< 0.1	< 0.1	0.989	1.49
L 4-2 B29	0.200	< 0.1	0.180	< 0.01	< 0.1	< 0.1	1.04	1.39
L 4-2 B30	0.281	0.100	0.146	< 0.01	< 0.1	< 0.1	0.738	1.93

* **Bold**: outlier due to contaminations or analytical errors.

Table S13. Laboratory irrigation results of the test specimen L 1-20 A: leachate amount, pH value, electrical conductivity and concentrations of sodium, potassium, calcium, chloride, and sulfate.

Day of Testing	Leachate	Amount of Leachate L/m² *	pH -	EC µS/cm	Na mg/L	K mg/L	Ca mg/L	Cl^- mg/L	SO_4^{2-} mg/L
1	L 1-20 A1	1.31	8.94	139	6.2	13.6	13.6	0.1	9.5
2	L 1-20 A2	12.1	9.10	94.6	3.0	7.9	6.0	< 0.1	2.4
3	L 1-20 A3	19.0	9.00	99.4	3.5	9.8	4.3	< 0.1	2.2
4	L 1-20 A4	5.78	8.50	73.3	3.0	8.3	5.9	< 0.1	1.1
5	L 1-20 A5	2.33	8.17	87.3	4.1	11.1	6.8	< 0.1	0.9
8	L 1-20 A6	2.65	8.16	101.5	6.3	16.7	5.4	< 0.1	1.1
9	L 1-20 A7	5.18	8.15	80.2	3.0	8.4	5.4	0.1	0.7
10	L 1-20 A8	7.91	8.14	69.5	2.8	7.6	4.5	0.1	0.9
11	L 1-20 A9	8.07	8.07	54.6	1.0	2.9	5.5	< 0.1	0.3
12	L 1-20 A10	5.33	8.09	60.9	2.3	6.4	5.0	< 0.1	0.4
15	L 1-20 A11	5.77	8.10	75.4	3.7	9.6	4.3	0.1	0.5
16	L 1-20 A12	3.96	8.05	58.7	2.6	6.8	3.7	< 0.1	0.5
17	L 1-20 A13	11.5	8.10	57.4	1.5	4.4	6.0	< 0.1	0.3
18	L 1-20 A14	12.1	8.03	55.2	0.5	1.4	5.4	< 0.1	0.2
19	L 1-20 A15	4.67	7.93	46.7	0.9	2.6	5.2	< 0.1	0.3
22	L 1-20 A16	10.9	7.36	34.2	0.7	2.1	4.9	0.1	0.2
23	L 1-20 A17	14.4	7.41	64.9	1.8	4.7	2.8	< 0.1	< 0.1
24	L 1-20 A18	25.7	7.37	49.7	1.0	3.0	4.3	< 0.1	0.7
25	L 1-20 A19	10.5	7.38	46.8	2.1	5.7	4.6	0.1	0.5
26	L 1-20 A20	4.04	7.33	43.7	1.5	4.2	5.4	0.1	0.3

* corresponding to m² of the test specimens´ surface.

Table S14. Laboratory irrigation results of the test specimen L 1-20 A: heavy metal and trace element concentrations, part 1.

Leachate	Sb µg/l	As µg/l	Ba µg/l	Pb µg/l	B µg/l	Cd µg/l	Cr µg/l	Co µg/l

L 1-20 A1	2.14	< 0.1	7.03	0.1430	5.39	< 0.1	0.670	< 0.1
L 1-20 A2	0.339	< 0.1	2.73	< 0.1	3.33	< 0.1	0.550	< 0.1
L 1-20 A3	0.256	< 0.1	3.63	< 0.1	4.96	< 0.1	0.558	< 0.1
L 1-20 A4	0.168	< 0.1	3.01	< 0.1	2.82	< 0.1	0.442	< 0.1
L 1-20 A5	0.476	< 0.1	3.70	< 0.1	3.34	< 0.1	0.470	< 0.1
L 1-20 A6	0.631	< 0.1	3.00	0.104	5.67	< 0.1	0.496	< 0.1
L 1-20 A7	0.128	< 0.1	2.31	0.100	3.77	< 0.1	0.402	< 0.1
L 1-20 A8	0.101	< 0.1	1.96	0.100	3.55	< 0.1	0.445	< 0.1
L 1-20 A9	< 0.1	< 0.1	1.97	< 0.1	2.25	< 0.1	0.336	< 0.1
L 1-20 A10	0.149	< 0.1	2.26	0.1240	4.35	< 0.1	0.365	< 0.1
L 1-20 A11	0.143	< 0.1	1.85	< 0.1	4.45	< 0.1	0.360	< 0.1
L 1-20 A12	< 0.1	< 0.1	1.67	< 0.1	3.63	< 0.1	0.330	< 0.1
L 1-20 A13	< 0.1	< 0.1	1.97	< 0.1	2.88	< 0.1	0.412	< 0.1
L 1-20 A14	< 0.1	< 0.1	1.84	< 0.1	2.23	< 0.1	0.350	< 0.1
L 1-20 A15	< 0.1	< 0.1	2.32	< 0.1	2.38	< 0.1	0.350	< 0.1
L 1-20 A16	< 0.1	< 0.1	1.83	< 0.1	2.02	< 0.1	0.327	< 0.1
L 1-20 A17	< 0.1	< 0.1	1.17	< 0.1	2.30	< 0.1	0.307	< 0.1
L 1-20 A18	< 0.1	< 0.1	1.88	< 0.1	2.46	< 0.1	0.387	< 0.1
L 1-20 A19	< 0.1	< 0.1	2.73	< 0.1	3.75	< 0.1	0.416	< 0.1
L 1-20 A20	< 0.1	< 0.1	2.43	< 0.1	3.47	< 0.1	0.381	< 0.1

Table S15. Laboratory irrigation results of the test specimen L 1-20 A: heavy metal and trace element concentrations, part 2.

Leachate	Cu µg/l	Mo µg/l	Ni µg/l	Hg µg/l	Se µg/l	Tl µg/l	V µg/l	Zn µg/l
L 1-20 A1	3.02	0.622	3.47	0.0101	0.118	< 0.1	1.30	3.98
L 1-20 A2	1.09	0.265	1.88	0.0121	0.131	< 0.1	1.23	2.13
L 1-20 A3	1.16	0.313	1.18	0.0189	0.111	< 0.1	1.34	1.74
L 1-20 A4	1.76	0.116	1.15	0.0136	< 0.1	< 0.1	0.716	1.80
L 1-20 A5	1.89	0.145	0.963	0.0117	< 0.1	< 0.1	1.02	4.48
L 1-20 A6	2.23	0.153	0.788	0.0187	< 0.1	< 0.1	1.39	3.90
L 1-20 A7	1.07	0.102	0.812	0.0233	< 0.1	< 0.1	0.867	2.06
L 1-20 A8	1.12	0.148	0.937	0.0151	< 0.1	< 0.1	1.25	3.54
L 1-20 A9	0.969	< 0.1	0.981	0.0192	< 0.1	< 0.1	0.415	3.16
L 1-20 A10	0.752	< 0.1	3.65	0.0151	< 0.1	< 0.1	0.799	2.61
L 1-20 A11	1.01	0.111	2.46	0.0105	< 0.1	< 0.1	1.40	2.53
L 1-20 A12	1.39	< 0.1	1.93	0.0181	< 0.1	< 0.1	0.912	6.02
L 1-20 A13	0.977	0.129	1.45	0.0100	< 0.1	< 0.1	0.799	1.13
L 1-20 A14	0.884	< 0.1	1.35	0.0108	< 0.1	< 0.1	0.487	2.96
L 1-20 A15	0.854	< 0.1	1.22	0.0117	< 0.1	< 0.1	0.552	3.89
L 1-20 A16	5.15	< 0.1	1.12	0.0112	< 0.1	< 0.1	0.461	2.41
L 1-20 A17	2.01	< 0.1	1.51	0.0161	< 0.1	< 0.1	0.732	1.65
L 1-20 A18	1.11	< 0.1	1.36	0.0145	< 0.1	< 0.1	0.592	1.29
L 1-20 A19	2.43	0.109	1.33	0.0114	< 0.1	< 0.1	1.25	3.42
L 1-20 A20	2.01	< 0.1	1.20	0.0149	< 0.1	< 0.1	0.703	4.67

Table S16. Laboratory irrigation results of the test specimen L 1-20 B: leachate amount, pH value, electrical conductivity and concentrations of sodium, potassium, calcium, chloride, and sulfate.

Day of Testing	Leachate	Amount of Leachate L/m² *	pH -	EC µS/cm	Na mg/L	K mg/L	Ca mg/L	Cl^- mg/L	SO_4^{2-} mg/L
1	L 1-20 B1	1.81	8.05	111	5.7	8.8	13.3	0.1	10.9

2	L 1-20 B2	13.5	9.09	61.2	1.9	5.1	4.1	< 0.1	3.0
3	L 1-20 B3	20.0	8.86	67.3	2.4	6.7	5.0	< 0.1	2.4
4	L 1-20 B4	5.55	8.30	75.5	2.7	7.3	7.3	< 0.1	1.4
5	L 1-20 B5	4.55	7.91	78.8	2.3	6.0	7.8	< 0.1	0.8
8	L 1-20 B6	5.27	7.96	88.8	4.6	12.4	6.0	< 0.1	1.3
9	L 1-20 B7	4.77	7.94	76.7	3.0	7.7	6.6	0.1	1.1
10	L 1-20 B8	4.9	7.88	80.6	2.9	7.9	6.9	0.1	0.8
11	L 1-20 B9	5.06	7.86	53.9	1.1	2.8	6.7	< 0.1	0.2
12	L 1-20 B10	5.2	7.83	67.5	2.5	6.5	6.3	< 0.1	0.4
15	L 1-20 B11	3.28	7.81	83.3	4.3	10.3	5.8	0.1	0.9
16	L 1-20 B12	6.46	7.87	61.1	2.4	6.4	5.2	< 0.1	0.4
17	L 1-20 B13	1.28	7.78	118.9	7.0	17.2	6.8	< 0.1	1.5
18	L 1-20 B14	11.5	7.81	57.9	0.8	2.1	6.4	< 0.1	0.2
19	L 1-20 B15	4.2	7.79	57.7	1.8	4.8	6.1	< 0.1	0.3
22	L 1-20 B16	10.3	7.34	38.1	0.8	2.4	5.5	0.1	0.3
23	L 1-20 B17	16.8	7.49	29.4	1.4	3.9	2.5	< 0.1	0.4
24	L 1-20 B18	23.4	7.47	47.7	1.1	3.3	4.8	< 0.1	0.4
25	L 1-20 B19	12.5	7.48	51.9	1.6	4.3	4.2	0.1	0.5
26	L 1-20 B20	5.77	7.40	50.6	1.3	3.9	5.0	0.1	0.2

* corresponding to m² of the test specimens´ surface.

Table S17. Laboratory irrigation results of the test specimen L 1-20 B: heavy metal and trace element concentrations, part 1.

Leachate	Sb µg/l	As µg/l	Ba µg/l	Pb µg/l	B µg/l	Cd µg/l	Cr µg/l	Co µg/l
L 1-20 B1	0	0.134	11.7	0.175	10.2	< 0.1	1.35	< 0.1
L 1-20 B2	0.496	< 0.1	5.35	0.270	6.27	< 0.1	0.963	< 0.1
L 1-20 B3	0.272	< 0.1	4.25	< 0.1	5.26	< 0.1	0.992	< 0.1
L 1-20 B4	0.258	< 0.1	3.54	< 0.1	5.06	< 0.1	0.753	< 0.1
L 1-20 B5	0.351	< 0.1	3.62	< 0.1	5.18	< 0.1	0.536	< 0.1
L 1-20 B6	0.164	0.103	2.82	< 0.1	5.39	< 0.1	0.608	< 0.1
L 1-20 B7	0.155	< 0.1	2.86	< 0.1	4.49	< 0.1	0.529	< 0.1
L 1-20 B8	0.153	< 0.1	2.41	< 0.1	4.41	< 0.1	0.555	< 0.1
L 1-20 B9	< 0.1	< 0.1	2.27	< 0.1	2.32	< 0.1	0.356	< 0.1
L 1-20 B10	< 0.1	< 0.1	2.49	< 0.1	3.12	< 0.1	0.377	< 0.1
L 1-20 B11	0.170	0.102	1.87	< 0.1	4.90	< 0.1	0.556	< 0.1
L 1-20 B12	< 0.1	< 0.1	1.69	< 0.1	3.38	< 0.1	0.390	< 0.1
L 1-20 B13	< 0.1	0.214	2.78	< 0.1	7.43	< 0.1	0.674	< 0.1
L 1-20 B14	< 0.1	< 0.1	1.63	< 0.1	2.40	< 0.1	0.388	< 0.1
L 1-20 B15	< 0.1	< 0.1	2.05	< 0.1	3.16	< 0.1	0.403	< 0.1
L 1-20 B16	< 0.1	< 0.1	1.64	< 0.1	2.09	< 0.1	0.332	< 0.1
L 1-20 B17	< 0.1	< 0.1	0.817	< 0.1	1.76	< 0.1	0.291	< 0.1
L 1-20 B18	< 0.1	< 0.1	1.45	< 0.1	3.19	< 0.1	0.354	< 0.1
L 1-20 B19	< 0.1	< 0.1	1.76	< 0.1	3.24	< 0.1	0.494	< 0.1
L 1-20 B20	< 0.1	< 0.1	2.14	0.106	4.42	< 0.1	0.385	< 0.1

Table S18. Laboratory irrigation results of the test specimen L 1-20 B: heavy metal and trace element concentrations, part 2.

Leachate	Cu µg/l	Mo µg/l	Ni µg/l	Hg µg/l	Se µg/l	Tl µg/l	V µg/l	Zn µg/l
L 1-20 B1	2.41	0.813	1.54	0.0136	0.140	< 0.1	2.70	1.56
L 1-20 B2	2.82	0.383	0.944	0.0101	0.175	< 0.1	1.96	7.23

L 1-20 B3	0.955	0.343	0.020	0.0124	< 0.1	< 0.1	2.00	< 1
L 1-20 B4	1.90	0.194	0.194	0.0100	< 0.1	< 0.1	1.61	1.51
L 1-20 B5	2.23	0.129	2.11	0.0131	< 0.1	< 0.1	1.08	2.11
L 1-20 B6	0.894	0.219	0.993	0.0106	< 0.1	< 0.1	1.69	1.00
L 1-20 B7	1.74	0.148	0.918	0.0138	< 0.1	< 0.1	1.11	9.95
L 1-20 B8	0.876	0.173	0.412	0.0208	< 0.1	< 0.1	1.44	< 1
L 1-20 B9	0.844	< 0.1	0.397	0.0141	< 0.1	< 0.1	0.455	2.03
L 1-20 B10	1.58	< 0.1	0.554	0.0148	< 0.1	< 0.1	0.894	1.84
L 1-20 B11	1.44	0.191	0.442	0.0104	< 0.1	< 0.1	1.94	1.50
L 1-20 B12	1.61	< 0.1	0.538	0.0136	< 0.1	< 0.1	0.978	8.09
L 1-20 B13	1.71	0.292	0.448	0.0168	< 0.1	< 0.1	3.35	1.56
L 1-20 B14	1.05	< 0.1	0.429	0.0134	< 0.1	< 0.1	0.666	2.84
L 1-20 B15	1.88	< 0.1	1.25	0.0181	< 0.1	< 0.1	0.898	3.77
L 1-20 B16	1.05	< 0.1	1.61	0.0192	< 0.1	< 0.1	0.568	2.76
L 1-20 B17	1.33	< 0.1	2.57	0.0136	< 0.1	< 0.1	0.687	1.35
L 1-20 B18	1.65	< 0.1	3.01	0.0153	< 0.1	< 0.1	0.714	< 1
L 1-20 B19	2.18	< 0.1	3.25	0.0190	< 0.1	< 0.1	1.04	5.93
L 1-20 B20	1.35	< 0.1	8.11	0.0231	< 0.1	< 0.1	0.730	4.54

Table 19. Laboratory irrigation results of the test specimen L 1-20 C: leachate amount, pH value, electrical conductivity and concentrations of sodium, potassium, calcium, chloride, and sulfate.

Day of Testing	Leachate	Amount of Leachate L/m² *	pH<br>-	EC<br>µS/cm	Na<br>mg/L	K<br>mg/L	Ca<br>mg/L	Cl^-<br>mg/L	SO_4^{2-}<br>mg/L
1	L 1-20 C1	6.28	10.19	173	12.7	35.4	1.6	< 0.1	1.0
2	L 1-20 C2	12.5	9.94	165.2	11.8	33.4	2.7	< 0.1	1.9
3	L 1-20 C3	48.4	9.71	69.6	3.4	8.9	4.7	< 0.1	0.7
4	L 1-20 C4	12.6	8.94	97.3	7.2	18.3	4.1	0.2	2.0
5	L 1-20 C5	6.20	8.18	73.1	4.7	12.2	4.1	< 0.1	0.8
8	L 1-20 C6	5.99	8.71	92.9	8.0	16.7	3.8	0.3	5.8
9	L 1-20 C7	12.3	7.72	58.5	3.8	8.9	4.2	< 0.1	1.5
10	L 1-20 C8	43.6	7.42	48.2	2.4	5.9	4.7	0.2	0.8
11	L 1-20 C9	12.5	7.59	47.2	1.6	4.3	5.8	< 0.1	1.2
12	L 1-20 C10	6.30	7.51	63.3	3.6	8.3	5.6	< 0.1	2.0
15	L 1-20 C11	6.25	7.74	71.0	4.7	9.0	4.9	0.2	5.8
16	L 1-20 C12	12.3	7.71	56	2.7	6.2	5.4	< 0.1	2.1
17	L 1-20 C13	36.7	8.84	43.1	1.1	2.8	5.4	< 0.1	1.5
18	L 1-20 C14	12.6	7.92	53.3	2.2	4.9	5.6	0.1	1.6
19	L 1-20 C15	8.75	7.67	59.4	1.6	4.3	6.0	3.0	3.0
22	L 1-20 C16	6.34	7.56	66.5	3.1	7.0	6.0	1.0	4.6
23	L 1-20 C17	13.4	7.49	49.9	1.7	4.2	5.6	0.6	2.2
24	L 1-20 C18	47.6	7.98	37.2	0.7	1.9	4.4	1.3	0.8
25	L 1-20 C19	12.7	7.94	56.4	1.4	3.6	7.1	0.8	2.8
26	L 1-20 C20	8.39	7.51	59.5	1.6	3.7	7.3	1.3	3.3

* corresponding to m² of the test specimens´ surface.

Table S20. Laboratory irrigation results of the test specimen L 1-20 C: heavy metal and trace element concentrations, part 1.

Leachate	Sb<br>µg/l	As<br>µg/l	Ba<br>µg/l	Pb<br>µg/l	B<br>µg/l	Cd<br>µg/l	Cr<br>µg/l	Co<br>µg/l
L 1-20 C1	0.797	< 0.1	8.49	0.120	2.44	< 0.1	0.497	< 0.1
L 1-20 C2	0.312	< 0.1	4.94	< 0.1	5.17	< 0.1	1.10	< 0.1

L 1-20 C3	< 0.1	< 0.1	5.21	< 0.1	2.75	< 0.1	0.482	< 0.1
L 1-20 C4	0.187	< 0.1	4.05	< 0.1	4.59	< 0.1	1.44	< 0.1
L 1-20 C5	0.118	< 0.1	4.05	< 0.1	3.17	< 0.1	0.796	< 0.1
L 1-20 C6	0.290	0.172	3.97	< 0.1	11.4	< 0.1	4.71	< 0.1
L 1-20 C7	0.148	0.164	2.41	< 0.1	6.64	< 0.1	1.90	< 0.1
L 1-20 C8	< 0.1	0.164	1.69	< 0.1	5.04	< 0.1	1.06	< 0.1
L 1-20 C9	< 0.1	0.136	2.19	< 0.1	5.16	< 0.1	1.25	< 0.1
L 1-20 C10	0.192	0.237	2.54	< 0.1	7.96	< 0.1	2.23	< 0.1
L 1-20 C11	0.174	0.278	2.43	< 0.1	10.8	< 0.1	3.45	< 0.1
L 1-20 C12	0.106	0.247	1.88	< 0.1	7.40	< 0.1	1.67	< 0.1
L 1-20 C13	< 0.1	0.170	1.80	< 0.1	4.07	< 0.1	0.797	< 0.1
L 1-20 C14	< 0.1	0.273	1.91	< 0.1	7.18	< 0.1	1.41	< 0.1
L 1-20 C15	< 0.1	0.236	1.67	< 0.1	6.71	< 0.1	1.21	< 0.1
L 1-20 C16	0.131	0.247	1.63	< 0.1	10.2	< 0.1	2.24	< 0.1
L 1-20 C17	< 0.1	0.222	1.32	< 0.1	6.71	< 0.1	1.23	< 0.1
L 1-20 C18	< 0.1	0.173	1.00	< 0.1	4.00	< 0.1	0.470	< 0.1
L 1-20 C19	0.113	0.326	1.68	< 0.1	8.66	< 0.1	1.22	< 0.1
L 1-20 C20	0.178	0.361	1.83	< 0.1	10.8	< 0.1	1.43	< 0.1

Table S21. Laboratory irrigation results of the test specimen L 1-20 C: heavy metal and trace element concentrations, part 2.

Leachate	Cu µg/l	Mo µg/l	Ni µg/l	Hg µg/l	Se µg/l	Tl µg/l	V µg/l	Zn µg/l
L 1-20 C1	0.696	0.435	0.591	< 0.01	< 0.1	< 0.1	0.858	2.26
L 1-20 C2	< 0.1	0.803	0.294	< 0.01	0.123	< 0.1	1.75	< 1
L 1-20 C3	< 0.1	0.349	0.339	< 0.01	< 0.1	< 0.1	0.830	< 1
L 1-20 C4	< 0.1	0.639	0.384	< 0.01	0.143	< 0.1	2.28	< 1
L 1-20 C5	< 0.1	0.339	0.533	< 0.01	< 0.1	< 0.1	1.33	1.35
L 1-20 C6	0.816	0.261	1.11	< 0.01	0.201	< 0.1	5.55	1.34
L 1-20 C7	0.630	< 0.1	1.07	< 0.01	< 0.1	< 0.1	2.93	< 1
L 1-20 C8	1.20	< 0.1	1.24	< 0.01	< 0.1	< 0.1	2.27	1.88
L 1-20 C9	0.866	< 0.1	1.14	< 0.01	< 0.1	< 0.1	2.38	1.78
L 1-20 C10	1.00	< 0.1	1.17	< 0.01	< 0.1	< 0.1	4.17	1.51
L 1-20 C11	0.816	< 0.1	0.992	< 0.01	0.133	< 0.1	5.05	2.89
L 1-20 C12	0.626	< 0.1	1.07	< 0.01	< 0.1	< 0.1	3.38	1.22
L 1-20 C13	0.465	< 0.1	0.778	< 0.01	< 0.1	< 0.1	2.02	1.08
L 1-20 C14	0.522	< 0.1	0.763	< 0.01	< 0.1	< 0.1	3.31	1.25
L 1-20 C15	0.660	0.318	2.03	< 0.01	< 0.1	< 0.1	2.71	< 1
L 1-20 C16	0.377	0.628	1.62	< 0.01	< 0.1	< 0.1	3.63	< 1
L 1-20 C17	0.311	0.307	1.43	< 0.01	< 0.1	< 0.1	2.86	< 1
L 1-20 C18	0.320	0.146	1.30	< 0.01	< 0.1	< 0.1	1.57	< 1
L 1-20 C19	0.681	0.336	1.25	< 0.01	0.103	< 0.1	3.29	< 1
L 1-20 C20	0.946	0.604	1.47	< 0.01	< 0.1	< 0.1	3.67	1.30

Table S22. Laboratory irrigation results of the test specimen L 1-20 D: leachate amount, pH value, electrical conductivity and concentrations of sodium, potassium, calcium, chloride, and sulfate.

Day of Testing	Leachate	Amount of Leachate L/m^2 *	pH -	EC µS/cm	Na mg/L	K mg/L	Ca mg/L	Cl^- mg/L	SO_4^{2-} mg/L
1	L 1-20 D1	7.13	10.76	161	8.2	20.4	1.9	< 0.1	4.1
2	L 1-20 D2	13.2	10.51	128.7	5.2	13.5	2.8	< 0.1	4.6
3	L 1-20 D3	20.3	9.49	75.4	3.1	7.3	8.2	< 0.1	2.4

4	L 1-20 D4	12.5	8.95	62.1	2.5	6.2	7.4	< 0.1	0.9
5	L 1-20 D5	6.24	8.73	82.8	5.1	12.2	5.9	0.1	2.0
8	L 1-20 D6	6.48	7.60	46.8	1.6	2.8	6.6	< 0.1	2.4
9	L 1-20 D7	12.5	7.29	39.2	1.6	3.4	4.7	< 0.1	1.1
10	L 1-20 D8	48.4	7.04	31.6	1.0	2.3	4.2	0.4	0.4
11	L 1-20 D9	13.6	7.50	42.6	1.2	2.9	6.0	0.3	1.0
12	L 1-20 D10	6.46	7.16	47.4	1.4	3.6	6.3	< 0.1	0.7
15	L 1-20 D11	6.43	7.35	64.7	2.0	4.3	8.6	0.1	1.4
16	L 1-20 D12	12.3	7.46	41.6	1.4	2.9	5.3	< 0.1	0.6
17	L 1-20 D13	44.0	7.44	31.1	0.6	1.5	4.2	< 0.1	0.2
18	L 1-20 D14	13.1	7.39	36.9	0.8	1.7	5.2	< 0.1	0.2
19	L 1-20 D15	9.43	7.46	42.6	0.8	2.1	5.7	0.4	0.5
22	L 1-20 D16	8.22	7.59	44.1	1.7	3.5	5.2	0.2	0.5
23	L 1-20 D17	14.6	7.44	34.4	0.9	2.0	4.8	0.2	0.3
24	L 1-20 D18	47.4	7.55	33.6	0.6	1.8	4.3	0.7	< 0.1
25	L 1-20 D19	12.3	7.55	40	0.3	1.2	5.5	1.2	< 0.1
26	L 1-20 D20	7.00	7.49	48.1	0.8	2.0	7.1	0.4	0.4

* corresponding to m² of the test specimens´ surface.

Table S23. Laboratory irrigation results of the test specimen L 1-20 D: heavy metal and trace element concentrations, part 1.

Leachate	Sb µg/l	As µg/l	Ba µg/l	Pb µg/l	B µg/l	Cd µg/l	Cr µg/l	Co µg/l
L 1-20 D1	1.03	< 0.1	14.3	0.111	4.60	< 0.1	0.848	< 0.1
L 1-20 D2	0.377	< 0.1	8.98	< 0.1	6.98	< 0.1	1.23	< 0.1
L 1-20 D3	0.262	< 0.1	5.14	< 0.1	7.04	< 0.1	1.32	< 0.1
L 1-20 D4	0.241	< 0.1	4.96	< 0.1	3.52	< 0.1	0.596	< 0.1
L 1-20 D5	0.222	0.185	3.80	< 0.1	7.96	< 0.1	1.15	< 0.1
L 1-20 D6	0.146	0.150	3.11	< 0.1	4.37	< 0.1	0.953	< 0.1
L 1-20 D7	0.111	0.175	2.26	< 0.1	4.43	< 0.1	0.742	< 0.1
L 1-20 D8	< 0.1	0.123	1.92	< 0.1	3.03	< 0.1	0.377	< 0.1
L 1-20 D9	< 0.1	0.208	2.08	< 0.1	4.63	< 0.1	0.536	< 0.1
L 1-20 D10	0.102	0.214	1.90	< 0.1	4.80	< 0.1	0.545	< 0.1
L 1-20 D11	0.116	0.289	2.27	< 0.1	6.27	< 0.1	0.658	< 0.1
L 1-20 D12	< 0.1	0.210	1.53	< 0.1	4.65	< 0.1	0.309	< 0.1
L 1-20 D13	< 0.1	0.148	1.32	< 0.1	2.62	< 0.1	0.171	< 0.1
L 1-20 D14	< 0.1	0.163	1.42	< 0.1	2.74	< 0.1	0.206	< 0.1
L 1-20 D15	< 0.1	0.190	1.35	< 0.1	4.55	< 0.1	0.227	< 0.1
L 1-20 D16	< 0.1	0.237	1.11	< 0.1	6.18	< 0.1	0.301	< 0.1
L 1-20 D17	< 0.1	0.191	0.938	< 0.1	3.36	< 0.1	0.146	< 0.1
L 1-20 D18	< 0.1	0.162	0.798	< 0.1	3.00	< 0.1	0.103	< 0.1
L 1-20 D19	< 0.1	0.157	1.04	< 0.1	2.65	< 0.1	0.145	< 0.1
L 1-20 D20	< 0.1	0.272	1.54	< 0.1	4.01	< 0.1	0.243	< 0.1

Table S24. Laboratory irrigation results of the test specimen L 1-20 D: heavy metal and trace element concentrations, part 2.

Leachate	Cu µg/l	Mo µg/l	Ni µg/l	Hg µg/l	Se µg/l	Tl µg/l	V µg/l	Zn µg/l
L 1-20 D1	0.220	1.44	0.508	< 0.01	0.220	< 0.1	2.51	< 1
L 1-20 D2	< 0.1	1.14	0.594	< 0.01	0.331	< 0.1	4.80	< 1
L 1-20 D3	< 0.1	0.637	0.908	< 0.01	0.196	< 0.1	4.42	< 1
L 1-20 D4	0.532	0.238	1.17	< 0.01	< 0.1	< 0.1	1.96	1.40

L 1-20 D5	0.216	0.328	0.925	< 0.01	0.136	< 0.1	5.21	1.17
L 1-20 D6	1.08	< 0.1	1.12	< 0.01	< 0.1	< 0.1	2.15	2.13
L 1-20 D7	0.921	< 0.1	1.06	< 0.01	< 0.1	< 0.1	2.35	1.76
L 1-20 D8	1.01	< 0.1	1.16	< 0.01	< 0.1	< 0.1	1.51	2.70
L 1-20 D9	0.879	< 0.1	1.06	< 0.01	< 0.1	< 0.1	1.94	2.15
L 1-20 D10	0.933	< 0.1	1.05	< 0.01	< 0.1	< 0.1	2.09	1.65
L 1-20 D11	0.944	< 0.1	0.868	< 0.01	< 0.1	< 0.1	2.31	3.34
L 1-20 D12	0.688	< 0.1	0.800	< 0.01	< 0.1	< 0.1	1.54	1.94
L 1-20 D13	0.618	< 0.1	0.846	< 0.01	< 0.1	< 0.1	1.01	1.71
L 1-20 D14	0.607	< 0.1	0.834	< 0.01	< 0.1	< 0.1	0.997	2.59
L 1-20 D15	0.729	0.180	1.41	< 0.01	< 0.1	< 0.1	1.21	1.50
L 1-20 D16	0.487	0.174	1.40	< 0.01	< 0.1	< 0.1	1.66	< 1
L 1-20 D17	0.490	< 0.1	1.45	< 0.01	< 0.1	< 0.1	1.03	1.78
L 1-20 D18	0.581	< 0.1	1.43	< 0.01	< 0.1	< 0.1	0.831	< 1
L 1-20 D19	0.951	< 0.1	1.45	< 0.01	< 0.1	< 0.1	0.794	1.92
L 1-20 D20	0.708	< 0.1	1.46	< 0.01	< 0.1	< 0.1	1.35	1.72

Table S25. Outdoor irrigation results of the test specimen F BW: leachate amount, pH value, electrical conductivity and concentrations of sodium, potassium, calcium, chloride, and sulfate.

Date of Sampling	Leachate Label	Amount L/m²	pH -	EC µS/cm	Na mg/L	K mg/L	Ca mg/L	Cl^- mg/L	SO_4^{2-} mg/L
25.10.2018	F-BW-1	3.36	6.8	80.6	4.4	0.5	2.9	5.4	5.8
01.11.2018	F-BW-2	18.0	6.2	11.3	0.6	0.0	0.4	0.7	0.3
08.11.2018	F-BW-3	2.33	6.9	19.2	2.2	0.1	0.8	0.5	5.0
15.11.2018	F-BW-4	7.46	6.8	13.9	0.2	0.0	0.2	1.9	< 0.1
29.11.2018	F-BW-5	7.13	7.0	18.7	0.1	0.1	1.0	0.6	1.9
06.12.2018	F-BW-6	25.3	5.7	52.0	< 0.1	< 0.1	0.1	0.6	< 0.1
13.12.2018	F-BW-7	27.3	5.8	29.0	3	< 0.1	0.2	2.8	< 0.1
20.12.2018	F-BW-8	4.81	5.9	22.0	1	< 0.1	0.4	1.6	< 0.1
27.12.2018	F-BW-9	30.0	5.9	10.0	< 0.1	< 0.1	0.0	0.4	< 0.1
03.01.2019	F-BW-10	12.9	5.6	32.0	3	< 0.1	0.2	2.6	1.3
10.01.2019	F-BW-11	24.0	5.8	42.0	4	1	0.3	3.7	1.7
17.01.2019	F-BW-12	37.1	6.4	12.0	1	< 0.1	0.1	0.8	1.2
24.01.2019	F-BW-13	2.43	5.9	36.0	4	< 0.1	0.4	2.3	1.0
31.01.2019	F-BW-14	1.98	6.7	17.0	< 0.1	< 0.1	0.6	0.2	0.3
07.02.2019	F-BW-15	6.67	5.4	46.0	8.0	< 0.1	0.2	1.0	0.9
14.02.2019	F-BW-16	25.4	6.0	13.0	2.0	< 0.1	0.1	2.7	0.5
21.02.2019	F-BW-17	0.43	5.7	224	7	1	2.9	1.0	10.1
28.02.2019	F-BW-18	0.85	6.6	73.0	2	1	2.4	7.1	5.3
07.03.2019	F-BW-19	24.9	6.6	38.0	0.8	0.3	0.4	1.4	5.2
14.03.2019	F-BW-20	51.7	6.3	34.0	1.5	0.2	0.2	3.1	0.8
21.03.2019	F-BW-21	31.7	5.1	11.6	0.7	0.1	0.0	1.4	< 0.1
28.03.2019	F-BW-22	0.33	4.6	137	18.4	2.2	7.9	39.4	15.1
04.04.2019	F-BW-23	4.98	6.0	136	0.6	0.2	0.8	0.9	1.2
11.04.2019	F-BW-24	0.54	3.8	136	0.6	1.4	3.8	12.3	8.9
18.04.2019	F-BW-25	0.29	4.9	105	0.9	1.3	9.4	15.1	19.0
25.04.2019	F-BW-26	7.38	6.5	39.6	0.2	0.1	0.7	0.5	1.4
02.05.2019	F-BW-27	9.49	6.2	18.9	1.3	0.1	0.3	4.2	4.2
09.05.2019	F-BW-28	15.0	6.2	25.8	0.7	0.0	0.2	1.5	0.6
16.05.2019	F-BW-29	7.97	6.2	24.0	0.1	0.0	0.0	0.6	0.3
23.05.2019	F-BW-30	8.63	6.6	31.7	0.6	0.1	0.8	1.5	1.5
30.05.2019	F-BW-31	12.2	6.4	9.1	0.3	0.1	0.2	0.7	5.5

06.06.2019	F-BW-32	24.2	6.2	7.3	0.1	0.1	0.3	0.2	0.4
13.06.2019	F-BW-33	10.1	6.3	13.0	0.2	0.1	0.4	2.4	1.1
20.06.2019	F-BW-34	7.99	7.1	24.0	0.3	0.2	2.2	0.6	0.6
18.07.2019	F-BW-38	8.84	6.6	17.0	0.1	< 0.1	1.1	0.3	2.2
25.07.2019	F-BW-39	7.95	n. d.	n. d.	0.3	0.1	0.9	2.9	< 0.1
01.08.2019	F-BW-40	20.7	n. d.	n. d.	0.0	0.0	0.5	< 0.1	0.5
08.08.2019	F-BW-41	3.62	6.4	28	0.6	0.2	1.5	3.3	1.4
15.08.2019	F-BW-42	13.6	6.60	18.0	0.3	0.0	0.6	1.4	0.5
22.08.2019	F-BW-43	20.6	6.4	6	0.0	0.0	0.2	0.9	0.4
05.09.2019	F-BW-45	4.27	6.3	43	2.2	0.4	3.6	6.3	2.4
12.09.2019	F-BW-46	8.71	6.3	29	1.5	0.0	0.4	4.7	1.3
19.09.2019	F-BW-47	1.35	3.5	166	0.9	0.0	2.3	21.7	4.6
26.09.2019	F-BW-48	8.39	6.5	12	0.7	< 0.1	0.6	1.2	0.9
03.10.2019	F-BW-49	25.4	6.4	9	0.5	< 0.1	< 0.1	0.9	0.5
10.10.2019	F-BW-50	35.6	6.4	7	0.4	< 0.1	< 0.1	0.8	0.5

Due to the absence of run-off, no samples were taken on the following dates: 18.10.2018, 22.11.2018, 27.06.2019, 04.07.2019, 11.07.2019, 29.08.2019; n. d. = not determined.

Table S26. Outdoor irrigation results of the test specimen F BW: heavy metal and trace element concentrations, part 1.

Leachate Label	Sb µg/l	As µg/l	Ba µg/l	Pb µg/l	B µg/l	Cd µg/l	Cr µg/l	Co µg/l
F-BW-1	0.578	0.492	9.05	2.39	14.7	0.125	0.746	0.369
F-BW-2	0.120	< 0.1	3.39	0.519	1.48	< 0.1	0.228	< 0.1
F-BW-3	< 0.1	< 0.1	1.47	0.212	1.34	< 0.1	0.139	< 0.1
F-BW-4	< 0.1	< 0.1	1.02	0.129	1.23	< 0.1	0.373	< 0.1
F-BW-5	0.295	0.205	5.23	2.35	3.03	0.120	0.351	0.101
F-BW-6	< 0.1	< 0.1	1.00	0.380	1.18	< 0.1	0.251	< 0.1
F-BW-7	< 0.1	< 0.1	0.870	0.315	2.28	< 0.1	< 0.1	< 0.1
F-BW-8	< 0.1	< 0.1	2.67	0.888	0.855	< 0.1	0.209	< 0.1
F-BW-9	< 0.1	< 0.1	0.650	< 0.1	0.619	< 0.1	< 0.1	< 0.1
F-BW-10	0.159	0.142	7.31	1.37	2.48	< 0.1	0.137	< 0.1
F-BW-11	0.195	0.165	2.31	1.80	3.55	< 0.1	0.17	< 0.1
F-BW-12	0.107	< 0.1	0.950	0.726	1.72	< 0.1	< 0.1	< 0.1
F-BW-13	0.114	< 0.1	1.76	1.10	3.39	< 0.1	< 0.1	0.117
F-BW-14	< 0.1	< 0.1	1.49	0.112	1.06	< 0.1	< 0.1	< 0.1
F-BW-15	0.162	0.112	1.58	2.00	1.74	< 0.1	0.240	0.106
F-BW-16	< 0.1	< 0.1	0.790	0.201	2.35	< 0.1	0.108	< 0.1
F-BW-17	0.502	0.231	7.05	7.42	4.50	0.141	0.791	0.225
F-BW-18	0.443	0.374	3.82	0.972	12.6	< 0.1	0.736	0.135
F-BW-19	0.140	< 0.1	1.97	0.542	2.57	< 0.1	0.155	< 0.1
F-BW-20	< 0.1	< 0.1	0.830	0.486	1.84	< 0.1	< 0.1	< 0.1
F-BW-21	< 0.1	< 0.1	0.790	0.454	1.11	< 0.1	< 0.1	< 0.1
F-BW-22	0.891	0.707	17.1	15.8	15.1	0.659	2.26	0.976
F-BW-23	0.212	0.144	4.50	2.79	1.98	< 0.1	0.597	0.166
F-BW-24	0.880	1.25	13.4	6.55	15.7	0.423	1.39	0.520
F-BW-25	1.030	0.954	20.8	9.21	7.70	0.272	2.16	0.904
F-BW-26	0.136	0.116	3.36	1.71	2.82	< 0.1	0.709	0.177
F-BW-27	0.149	< 0.1	1.55	0.546	3.72	< 0.1	0.225	< 0.1
F-BW-28	0.157	< 0.1	1.90	1.87	2.81	< 0.1	0.364	< 0.1
F-BW-29	0.097	< 0.1	1.37	0.559	2.19	< 0.1	0.173	< 0.1
F-BW-30	0.233	0.209	3.15	0.729	5.03	< 0.1	0.283	< 0.1

F-BW-31	< 0.1	< 0.1	1.00	0.574	2.42	< 0.1	0.165	< 0.1
F-BW-32	0.136	< 0.1	1.26	0.430	3.85	< 0.1	0.207	< 0.1
F-BW-33	0.104	< 0.1	1.41	0.654	2.66	< 0.1	0.261	< 0.1
F-BW-34	0.135	0.128	5.43	0.885	4.60	< 0.1	0.381	0.103
F-BW-38	0.199	0.167	4.16	1.43	5.52	< 0.1	0.474	< 0.1
F-BW-39	0.123	< 0.1	3.76	1.18	6.13	< 0.1	0.389	0.14
F-BW-40	0.122	0.126	2.18	0.414	6.64	< 0.1	0.228	< 0.1
F-BW-41	0.196	0.161	3.76	2.01	7.19	< 0.1	0.501	0.118
F-BW-42	< 0.1	< 0.1	1.38	0.515	4.85	< 0.1	0.279	< 0.1
F-BW-43	< 0.1	< 0.1	0.556	0.172	3.11	< 0.1	0.136	< 0.1
F-BW-45	0.399	0.323	8.75	5.42	12.1	0.368	1.29	0.364
F-BW-46	0.176	< 0.1	1.93	0.805	3.95	<0.1	0.230	< 0.1
F-BW-47	0.602	0.383	7.33	4.07	7.73	0.178	1.45	0.382
F-BW-48	0.241	0.138	3.22	2.36	9.78	< 0.1	0.585	< 0.1
F-BW-49	< 0.1	< 0.1	0.782	0.318	3.66	< 0.1	0.151	< 0.1
F-BW-50	< 0.1	< 0.1	0.712	0.317	3.89	< 0.1	0.102	< 0.1

Table S27. Outdoor irrigation results of the test specimen F BW: heavy metal and trace element concentrations, part 2.

Leachate	Cu µg/l	Mo µg/l	Ni µg/l	Hg µg/l	Se µg/l	Tl µg/l	V µg/l	Zn µg/l
F-BW-1	8.87	< 0.1	4.13	< 0.01	0.75	< 0.1	1.16	49.0
F-BW-2	2.52	< 0.1	0.997	< 0.01	< 0.1	< 0.1	0.308	10.7
F-BW-3	1.20	< 0.1	1.26	< 0.01	< 0.1	< 0.1	0.199	9.71
F-BW-4	1.19	< 0.1	1.84	< 0.01	< 0.1	< 0.1	< 0.1	6.63
F-BW-5	5.45	0.329	1.25	0.019	0.222	< 0.1	0.221	25.5
F-BW-6	1.16	0.414	< 0.1	< 0.01	< 0.1	< 0.1	0.235	5.05
F-BW-7	0.643	0.153	0.291	< 0.01	< 0.1	< 0.1	0.146	5.77
F-BW-8	1.70	0.130	0.393	< 0.01	< 0.1	< 0.1	0.168	8.79
F-BW-9	0.734	< 0.1	< 0.1	< 0.01	< 0.1	< 0.1	0.145	3.36
F-BW-10	3.4	< 0.1	< 0.1	< 0.01	0.192	< 0.1	0.349	10.4
F-BW-11	3.49	0.220	1.68	< 0.01	0.379	< 0.1	0.42	14.6
F-BW-12	0.986	0.139	0.562	< 0.01	0.177	< 0.1	0.207	7.26
F-BW-13	1.73	0.185	1.21	< 0.01	0.303	< 0.1	< 0.1	12.4
F-BW-14	0.908	< 0.1	< 0.1	< 0.01	< 0.1	< 0.1	< 0.1	5.03
F-BW-15	2.28	0.279	1.95	< 0.01	0.122	< 0.1	0.108	12.9
F-BW-16	0.358	0.230	1.37	0.0114	< 0.1	< 0.1	< 0.1	5.13
F-BW-17	8.93	0.291	2.49	0.0152	0.339	< 0.1	1.02	40.5
F-BW-18	3.72	0.946	2.82	< 0.01	0.369	< 0.1	1.1	21.6
F-BW-19	1.02	0.234	0.827	< 0.01	0.109	< 0.1	0.245	8.53
F-BW-20	0.521	0.117	0.514	< 0.01	< 0.1	< 0.1	< 0.1	6.15
F-BW-21	0.287	0.103	0.364	< 0.01	< 0.1	< 0.1	< 0.1	5.97
F-BW-22	29.1	1.55	0.862	0.0146	1.14	< 0.1	2.14	154
F-BW-23	4.06	0.329	1.36	< 0.01	0.114	< 0.1	0.519	21.6
F-BW-24	16.5	0.575	4.43	0.0147	0.822	< 0.1	1.16	92.8
F-BW-25	24.4	0.931	6.96	< 0.01	0.495	< 0.1	2.19	105
F-BW-26	2.94	0.356	4.72	< 0.01	0.116	< 0.1	0.393	18.1
F-BW-27	1.36	0.316	0.644	< 0.01	0.101	< 0.1	< 0.1	9.26
F-BW-28	2.21	0.223	0.895	< 0.01	0.115	< 0.1	0.205	25.1
F-BW-29	1.13	0.238	0.646	< 0.01	< 0.1	< 0.1	0.103	12.0
F-BW-30	2.32	0.540	0.974	< 0.01	0.639	< 0.1	0.414	18.8
F-BW-31	0.895	0.337	0.408	< 0.01	0.138	< 0.1	0.148	12.6

F-BW-32	1.15	0.257	0.488	< 0.01	< 0.1	< 0.1	0.257	9.26
F-BW-33	1.44	0.251	0.568	< 0.01	< 0.1	< 0.1	0.321	9.20
F-BW-34	1.67	0.317	0.747	< 0.01	< 0.1	< 0.1	0.844	4.82
F-BW-38	2.79	0.706	1.29	<0.01	0.143	<0.1	0.64	18.4
F-BW-39	2.71	**28.8**	1.84	<0.01	<0.1	<0.1	0.263	20.0
F-BW-40	1.47	**9.29**	1.67	<0.01	0.188	<0.1	0.222	9.29
F-BW-41	4.89	**5.78**	3.49	<0.01	0.148	<0.1	0.497	21.6
F-BW-42	1.05	**2.85**	2.44	<0.01	<0.1	<0.1	0.211	8.43
F-BW-43	0.341	**1.97**	1.31	<0.01	<0.1	<0.1	<0.1	3.02
F-BW-45	7.29	**1100**	2.61	<0.01	0.223	<0.1	1.22	39.3
F-BW-46	1.68	<0.1	0.738	<0.01	0.231	<0.1	0.255	17.1
F-BW-47	6.76	<0.1	2.79	<0.01	0.762	<0.1	1.01	46.5
F-BW-48	3.53	0.959	1.16	<0.01	<0.1	<0.1	0.421	23.2
F-BW-49	0.578	0.463	0.749	<0.01	<0.1	<0.1	0.208	6.60
F-BW-50	0.497	1.00	0.583	<0.1	<0.1	<0.1	<0.1	4.63

* **Bold**: Significantly increased blank value, probably due to contaminations during analysis or particle deposition.

Table S28. Outdoor irrigation results of the test specimen F 1 A: leachate amount, pH value, electrical conductivity and concentrations of sodium, potassium, calcium, chloride, and sulfate.

Date of Sampling	Leachate Label	Amount L/m²	pH -	EC µS/cm	Na mg/L	K mg/L	Ca mg/L	Cl^- mg/L	SO_4^{2-} mg/L
25.10.2018	F1-A 1	3.22	10.2	1051	94.1	218	1.8	3.8	29.2
01.11.2018	F1-A 2	13.0	9.65	166	14.1	42.7	2	0.6	1.8
08.11.2018	F1-A 3	2.01	9.03	188	21.0	46.2	2.5	0.5	2.6
15.11.2018	F1-A 4	6.50	9.22	202	16.9	50.7	1.6	0.8	1.8
29.11.2018	F1-A 5	6.60	9.1	158	16.7	46.9	1.5	0.1	3.3
06.12.2018	F1-A 6	25.3	7.6	42	3	9	3.5	0.5	1
13.12.2018	F1-A 7	21.7	7.3	54	4	5	4	2.2	0.6
20.12.2018	F1-A 8	3.18	7.5	60	7	10	2.8	0.9	1
27.12.2018	F1-A 9	24.2	7.4	45	2	5	4.3	0.7	< 0.1
03.01.2019	F1-A 10	9.37	7.8	22	6	9	5	1.5	1.5
10.01.2019	F1-A 11	16.6	7.5	33	6	5	5.4	3.2	1.5
17.01.2019	F1-A 12	25.4	7.5	16	3	3	5	1.4	0.5
24.01.2019	F1-A 13	1.20	7.5	50	3	3	5.2	2.5	1.1
31.01.2019	F1-A 14	21.7	7.8	22	2	3	4.6	0.7	0.5
07.02.2019	F1-A 15	5.08	6.6	152	2.5	7	5.2	1.0	1.3
14.02.2019	F1-A 16	25.4	6.8	70	3	3	4.6	2.1	0.6
21.02.2019	F1-A 17	0.30	7.1	196	13	14	9.4	1.0	8.1
28.02.2019	F1-A 18	0.69	7.3	149	15	18	6.6	1.0	7
07.03.2019	F1-A 19	19.4	7.2	84	3.6	7.2	3.7	1.2	2.8
14.03.2019	F1-A 20	42.8	7.3	88	2.7	3.8	3.8	2.8	0.7
21.03.2019	F1-A 21	26.2	7.3	42	1.6	2.7	5.2	1.3	0.6
28.03.2019	F1-A 22	0.42	6.9	193	27.3	22.3	7.8	33.5	18.2
04.04.2019	F1-A 23	4.77	6.9	54	4.6	7.4	3.1	0.9	2.8
11.04.2019	F1-A 24	0.70	6.8	148	14.8	27.2	4.9	7.1	14.6
18.04.2019	F1-A 25	0.30	6.8	262	25.6	40.3	8.6	40.2	17.4
25.04.2019	F1-A 26	5.82	6.9	49	1.9	5.4	8.6	1.0	1.9
02.05.2019	F1-A 27	7.36	7.2	68	3.7	7.2	5.1	2.6	0.5
09.05.2019	F1-A 28	16.0	7.1	70	4.8	8.9	3.4	1.3	0.6
16.05.2019	F1-A 29	7.12	7.2	63	3.3	8.7	3.6	0.8	5.4
23.05.2019	F1-A 30	7.22	7.3	9	6.8	15.9	4.5	1.4	3.6

30.05.2019	F1-A 31	9.80	7.7	50	2.5	7.4	4.1	0.9	2.1
06.06.2019	F1-A 32	19.7	6.9	34	0.9	2.8	4.0	0.2	0.9
13.06.2019	F1-A 33	7.50	6.6	51	2.6	5.6	5.0	0.7	2.1
20.06.2019	F1-A 34	7.85	7.1	57	2.7	6.1	7.0	1.3	3.4
18.07.2019	F1-A 38	6.87	6.8	50	1.7	3.3	6.1	0.7	3.9
25.07.2019	F1-A 39	6.35	n. d.	n. d.	1.6	3.3	4.1	1.7	0.7
01.08.2019	F1-A 40	14.5	n. d.	n. d.	2.4	5.2	7.4	0.3	2.4
08.08.2019	F1-A 41	2.33	6.9	138	17.2	19.2	3.2	11.0	8.1
15.08.2019	F1-A 42	11.6	7	74	8.4	16.0	2.5	0.4	2.9
22.08.2019	F1-A 43	12.1	6.9	52	3.6	9.9	2.8	2.0	3.7
05.09.2019	F1-A 45	3.40	7.1	104	7.9	16.6	6.5	2.7	4.9
12.09.2019	F1-A 46	8.25	7.3	95	7.4	13.1	4.0	6.2	2.7
19.09.2019	F1-A 47	1.54	7.3	142	10.9	21.9	3.9	8.8	7.1
26.09.2019	F1-A 48	7.46	7.1	85	7.4	16.2	3.4	0.6	2.1
03.10.2019	F1-A 49	25.3	7.1	48	3.0	7.4	3.2	0.9	1
10.10.2019	F1-A 50	29.8	7.5	44	3.4	7.2	4.0	1.1	0.8

Due to the absence of run-off, no samples were taken on the following dates: 18.10.2018, 22.11.2018, 27.06.2019, 04.07.2019, 11.07.2019, 29.08.2019; n. d. = not determined.

Table S29. Outdoor irrigation results of the test specimen F 1 A: heavy metal and trace element concentrations, part 1.

Leachate	Sb μg/l	As μg/l	Ba μg/l	Pb μg/l	B μg/l	Cd μg/l	Cr μg/l	Co μg/l
F1-A 1	1.30	5.79	4.04	0.768	109	< 0.1	8.93	0.206
F1-A 2	0.194	0.578	1.77	0.524	11.3	< 0.1	0.944	0.118
F1-A 3	0.235	1.1	2.10	0.205	20.5	< 0.1	1.35	< 0.1
F1-A 4	0.174	0.965	0.822	0.151	15.3	< 0.1	1.49	< 0.1
F1-A 5	0.283	1.11	1.8	0.481	16.5	< 0.1	0.719	< 0.1
F1-A 6	< 0.1	< 0.1	0.752	< 0.1	3.85	< 0.1	0.158	< 0.1
F1-A 7	< 0.1	< 0.1	0.628	< 0.1	3.35	< 0.1	0.12	< 0.1
F1-A 8	< 0.1	0.130	1.19	0.388	3.92	< 0.1	0.273	< 0.1
F1-A 9	< 0.1	< 0.1	0.877	0.107	2.04	< 0.1	< 0.1	< 0.1
F1-A 10	0.159	0.21	1.79	0.276	4.29	< 0.1	0.173	< 0.1
F1-A 11	0.177	0.181	1.81	0.387	4.38	< 0.1	0.127	< 0.1
F1-A 12	0.115	0.112	1.17	0.447	2.29	< 0.1	0.154	< 0.1
F1-A 13	< 0.1	< 0.1	1.74	0.212	2.82	< 0.1	< 0.1	< 0.1
F1-A 14	< 0.1	< 0.1	1.21	0.182	1.81	< 0.1	< 0.1	< 0.1
F1-A 15	0.176	0.171	2.50	0.954	2.95	< 0.1	0.390	< 0.1
F1-A 16	< 0.1	0.110	0.794	0.253	2.22	< 0.1	0.114	< 0.1
F1-A 17	0.530	0.325	6.33	2.99	10.9	< 0.1	1.90	< 0.1
F1-A 18	0.509	0.564	3.80	0.774	21.2	< 0.1	1.18	0.126
F1-A 19	0.150	0.166	1.35	0.510	4.18	< 0.1	0.312	< 0.1
F1-A 20	< 0.1	< 0.1	1.24	0.481	3.04	< 0.1	0.129	< 0.1
F1-A 21	< 0.1	< 0.1	0.86	0.120	2.09	< 0.1	< 0.1	< 0.1
F1-A 22	0.850	1.02	11.5	5.12	22.5	0.127	2.15	0.753
F1-A 23	0.227	0.232	6.08	1.77	8.16	< 0.1	2.89	0.401
F1-A 24	0.975	1.42	8.74	3.91	27.4	< 0.1	2.98	0.602
F1-A 25	1.40	2.09	20.9	7.46	41.5	0.136	4.73	1.06
F1-A 26	0.163	0.181	3.32	1.54	4.17	< 0.1	1.32	0.347
F1-A 27	0.172	0.197	2.16	1.18	9.22	< 0.1	0.942	0.155
F1-A 28	0.151	0.327	1.66	0.993	7.84	< 0.1	0.602	< 0.1
F1-A 29	0.111	0.259	1.39	0.264	7.78	< 0.1	0.520	0.116

F1-A 30	0.287	0.586	2.80	0.709	15.7	< 0.1	0.848	0.110
F1-A 31	0.125	0.232	1.53	0.618	7.52	< 0.1	0.389	0.105
F1-A 32	0.131	0.118	1.15	0.372	6.47	< 0.1	0.529	< 0.1
F1-A 33	0.136	0.223	1.57	0.427	10.3	< 0.1	0.628	< 0.1
F1-A 34	0.153	0.279	3.83	0.859	13.4	< 0.1	0.848	< 0.1
F1-A 38	0.201	0.245	3.84	1.28	12.1	< 0.1	1.58	0.520
F1-A 39	0.117	0.208	2.50	0.989	12.6	< 0.1	0.992	0.165
F1-A 40	0.147	0.280	2.12	0.263	16.1	< 0.1	0.981	0.121
F1-A 41	0.357	0.944	4.91	2.70	28.2	< 0.1	3.09	0.315
F1-A 42	0.148	0.672	1.36	0.357	20.7	< 0.1	1.35	< 0.1
F1-A 43	< 0.1	0.364	1.07	0.374	9.52	< 0.1	0.564	< 0.1
F1-A 45	0.376	0.707	4.84	2.31	31.3	0.134	2.08	0.322
F1-A 46	0.208	0.748	1.91	0.367	24.9	< 0.1	1.56	< 0.1
F1-A 47	0.574	1.11	4.34	1.97	31.9	< 0.1	1.94	0.277
F1-A 48	0.215	0.888	2.18	0.575	28.8	< 0.1	1.21	< 0.1
F1-A 49	< 0.1	0.306	0.845	0.134	9.36	< 0.1	0.415	< 0.1
F1-A 50	< 0.1	0.317	1.06	0.171	34.0	< 0.1	0.253	< 0.1

Table S30. Outdoor irrigation results of the test specimen F 1 A: heavy metal and trace element concentrations, part 2.

Leachate	Cu µg/l	Mo µg/l	Ni µg/l	Hg µg/l	Se µg/l	Tl µg/l	V µg/l	Zn µg/l
F1-A 1	4.00	3.06	2.39	< 0.01	2.72	< 0.1	53.0	4.80
F1-A 2	1.25	< 0.1	1.20	< 0.01	0.213	< 0.1	4.15	4.10
F1-A 3	1.55	< 0.1	1.03	< 0.01	0.261	< 0.1	7.63	2.19
F1-A 4	1.25	0.343	2.65	< 0.01	0.202	< 0.1	5.30	< 0.1
F1-A 5	2.16	0.439	1.31	< 0.01	0.343	< 0.1	6.31	3.81
F1-A 6	0.284	0.307	< 0.1	< 0.01	< 0.1	< 0.1	0.926	< 0.1
F1-A 7	0.333	0.216	< 0.1	< 0.01	< 0.1	< 0.1	0.623	< 0.1
F1-A 8	1.12	0.229	< 0.1	< 0.01	< 0.1	< 0.1	0.884	3.03
F1-A 9	0.343	0.109	< 0.1	< 0.01	< 0.1	< 0.1	0.503	< 0.1
F1-A 10	1.39	0.171	< 0.1	< 0.01	0.239	< 0.1	0.969	1.49
F1-A 11	1.13	0.224	< 0.1	< 0.01	0.362	< 0.1	0.691	4.08
F1-A 12	0.994	0.146	< 0.1	< 0.01	0.210	< 0.1	0.448	3.03
F1-A 13	0.931	0.149	< 0.1	< 0.01	0.309	< 0.1	< 0.1	3.57
F1-A 14	0.432	< 0.1	< 0.1	< 0.01	0.115	< 0.1	0.108	2.94
F1-A 15	2.71	0.316	1.43	< 0.01	0.172	< 0.1	0.650	8.59
F1-A 16	0.640	0.148	0.930	< 0.01	0.154	< 0.1	0.374	2.68
F1-A 17	11.7	0.397	1.42	< 0.01	0.345	< 0.1	1.89	38.6
F1-A 18	5.86	1.32	2.21	< 0.01	0.586	< 0.1	3.25	8.72
F1-A 19	1.26	0.225	0.483	< 0.01	< 0.1	< 0.1	1.01	3.66
F1-A 20	0.557	0.127	0.239	< 0.01	< 0.1	< 0.1	0.366	2.91
F1-A 21	0.170	< 0.1	< 0.1	< 0.01	< 0.1	< 0.1	0.412	1.03
F1-A 22	20.1	1.55	3.63	0.0156	1.05	< 0.1	5.88	47.2
F1-A 23	4.11	1.84	14.5	0.0160	0.181	< 0.1	1.63	17.1
F1-A 24	17.0	2.48	24.2	0.0115	0.941	< 0.1	3.83	26.6
F1-A 25	32.0	2.76	13.7	0.0134	0.644	< 0.1	12.1	58.4
F1-A 26	3.56	0.620	13.9	< 0.01	0.145	< 0.1	1.02	13.0
F1-A 27	4.95	0.962	17.8	< 0.01	0.166	< 0.1	1.20	14.4
F1-A 28	2.97	0.309	1.36	< 0.01	0.157	< 0.1	1.66	11.2
F1-A 29	0.943	0.292	4.32	< 0.01	0.113	< 0.1	1.37	3.44
F1-A 30	3.16	0.879	5.56	< 0.01	0.610	< 0.1	3.59	6.47

F1-A 31	1.33	0.360	0.630	< 0.01	0.183	< 0.1	1.09	6.32
F1-A 32	1.02	0.342	0.820	< 0.01	< 0.1	< 0.1	0.801	4.28
F1-A 33	1.74	0.370	0.395	< 0.01	0.116	< 0.1	1.96	6.56
F1-A 34	1.72	0.395	0.647	< 0.01	0.119	< 0.1	2.75	4.28
F1-A 38	3.73	1.99	45.3	< 0.01	0.225	< 0.1	1.87	14.9
F1-A 39	2.40	**23.3**	11.0	< 0.01	< 0.1	< 0.1	1.68	6.50
F1-A 40	1.13	**9.28**	5.73	< 0.01	0.216	< 0.1	3.14	12.2
F1-A 41	6.80	**6.77**	6.50	< 0.01	0.487	< 0.1	7.22	34.1
F1-A 42	1.17	**3.22**	2.50	< 0.01	0.189	< 0.1	6.37	3.42
F1-A 43	0.775	**1.83**	1.73	< 0.01	< 0.1	< 0.1	2.77	3.82
F1-A 45	5.12	**591**	4.89	< 0.01	0.407	< 0.1	5.00	16.4
F1-A 46	1.32	< 0.1	1.57	< 0.01	0.331	< 0.1	6.56	4.37
F1-A 47	4.24	0.429	4.80	< 0.01	1.07	< 0.1	6.01	15.9
F1-A 48	1.71	1.34	5.05	< 0.01	0.167	< 0.1	6.34	6.44
F1-A 49	0.493	0.608	5.36	< 0.01	0.115	< 0.1	2.02	< 1
F1-A 50	0.524	1.00	0.658	< 0.01	0.108	< 0.1	1.51	< 1

Bold: Significantly increased blank values for molybdenum, probably through contaminations during analysis or by particle deposits, led to likewise increased concentrations for the test specimens.

Table S31. Outdoor irrigation results of the test specimen F 1 B: leachate amount, pH value, electrical conductivity and concentrations of sodium, potassium, calcium, chloride, and sulfate.

Date of Sampling	Leachate Label	Amount L/m²	pH -	EC µS/cm	Na mg/L	K mg/L	Ca mg/L	Cl^- mg/L	SO_4^{2-} mg/L
25.10.2018	F1 B-1	3.20	10.3	997	87.9	207.8	1.9	3.8	42.3
01.11.2018	F1 B-2	13.5	9.67	161	12.5	42.6	2.1	0.7	3.9
08.11.2018	F1 B-3	1.89	9.17	187	22.1	48.6	2.5	0.2	4.4
15.11.2018	F1 B-4	6.18	9.35	212	17.4	53.5	1.8	0.5	2.6
29.11.2018	F1 B-5	6.48	9.04	170	16.1	47	1.8	0.2	3.3
06.12.2018	F1 B-6	25.3	7.1	53	3	9	3.7	0.4	0.6
13.12.2018	F1 B-7	21.7	6.8	57	5	6	4.6	2.5	1.1
20.12.2018	F1 B-8	5.52	7.4	55	6	7	3.3	0.6	< 0.1
27.12.2018	F1 B-9	25.1	7.2	41	2	4	4.6	0.3	< 0.1
03.01.2019	F1 B-10	10.1	7.3	71	6	8	6	1.6	1.1
10.01.2019	F1 B-11	19.4	7	68	6	5	6	3.2	1.1
17.01.2019	F1 B-12	29.7	7.2	46	3	3	5.3	0.9	0.6
24.01.2019	F1 B-13	1.89	7.3	60	3	3	7.1	2.9	0.9
31.01.2019	F1 B-14	2.59	7	182	0	0	2.9	0.3	0.4
07.02.2019	F1 B-15	6.14	7.4	82	9	5	6.1	1.0	1.8
14.02.2019	F1 B-16	24.8	7.4	76	11	2	5	1.0	2.4
21.02.2019	F1 B-17	0.32	5.2	367	13	13	10.9	1.0	7.6
28.02.2019	F1 B-18	0.60	7.3	148	14	16	8.1	6.4	10.8
07.03.2019	F1 B-19	17.8	7.2	80	4	8.1	4	1.2	1.5
14.03.2019	F1 B-20	44.9	7.3	75	3.2	4.8	4.1	3.3	0.8
21.03.2019	F1 B-21	28.7	7.2	42	1.4	2.2	5.4	1.1	0.2
28.03.2019	F1 B-22	0.43	6.9	231	27.4	23	8.8	30.9	15.2
04.04.2019	F1 B-23	5.02	7.1	59	4.6	7.5	3.7	0.6	2.6
11.04.2019	F1 B-24	0.67	6.8	184	15.5	28.1	6.3	5.8	14.6
18.04.2019	F1 B-25	0.31	6.4	339	31.9	48.4	8.4	52.2	20.9
25.04.2019	F1 B-26	4.74	6.9	70	2.6	7.1	4.8	1.7	1.6
02.05.2019	F1 B-27	7.24	7.1	79	3.9	8	5.5	4.6	0.6
09.05.2019	F1 B-28	15.0	7.1	77	4.9	9.7	4	1.1	0.7

16.05.2019	F1 B-29	7.63	7.2	68	3	8.7	3.8	3.2	1.2
23.05.2019	F1 B-30	8.00	7.3	55	6.4	15.9	5.1	1.6	4.7
30.05.2019	F1 B-31	11.5	7.5	55	2.9	8.2	4.3	1.2	1.6
06.06.2019	F1 B-32	20.5	7	32	0.9	3.2	4.4	0.7	0.7
13.06.2019	F1 B-33	8.37	6.9	42	2.4	5.6	5.1	0.3	1.9
20.06.2019	F1 B-34	7.39	7.2	61	2.2	4.9	7.4	0.9	2.4
18.07.2019	F1 B-38	8.03	7.2	50	1.4	3.6	5.7	2.0	2.5
25.07.2019	F1 B-39	6.70	n. d.	n. d.	1.7	3.7	4.6	0.9	0.7
01.08.2019	F1 B-40	20.3	n. d.	n. d.	2.1	5	7.3	0.3	0.6
08.08.2019	F1 B-41	2.71	7.1	133	18.1	27	2.8	3.1	7.4
15.08.2019	F1 B-42	10.4	7.10	79.00	8	17.5	2.9	0.6	2.0
22.08.2019	F1 B-43	14.5	7	51	3.2	9.7	3	0.9	1.2
05.09.2019	F1 B-45	3.37	7.1	102	7.8	17.3	6.8	6.9	5.5
12.09.2019	F1 B-46	7.92	7.3	97	7.2	14.1	4.5	3.1	2.9
19.09.2019	F1 B-47	1.33	7.2	151	11.2	25.6	4.3	16.7	6.6
26.09.2019	F1 B-48	7.01	7.3	95	7.4	17.8	3.7	0.7	2.8
03.10.2019	F1 B-49	25.3	7.2	40	2.8	7.1	3.2	1.1	1.0
10.10.2019	F1 B-50	29.8	7.3	42	3.1	7.0	4.5	0.8	0.4

Due to the absence of run-off, no samples were taken on the following dates: 18.10.2018, 22.11.2018, 27.06.2019, 04.07.2019, 11.07.2019, 29.08.2019; n. d. = not determined.

Table S32. Outdoor irrigation results of the test specimen F 1 B: heavy metal and trace element concentrations, part 1.

Leachate	Sb µg/l	As µg/l	Ba µg/l	Pb µg/l	B µg/l	Cd µg/l	Cr µg/l	Co µg/l
F1 B-1	1.39	6.56	2.90	1.05	131	< 0.1	13.4	0.276
F1 B-2	0.163	0.562	1.57	0.279	13.0	< 0.1	1.24	< 0.1
F1 B-3	0.281	0.961	2.88	0.226	25.6	< 0.1	1.96	< 0.1
F1 B-4	0.201	0.839	1.13	0.208	20.0	< 0.1	1.50	< 0.1
F1 B-5	0.367	0.984	1.88	0.549	19.6	< 0.1	0.828	< 0.1
F1 B-6	< 0.1	0.133	0.542	< 0.1	3.85	< 0.1	0.202	< 0.1
F1 B-7	< 0.1	0.111	0.862	0.187	3.86	< 0.1	0.120	< 0.1
F1 B-8	< 0.1	0.121	1.21	0.146	3.54	< 0.1	0.257	< 0.1
F1 B-9	< 0.1	< 0.1	0.588	< 0.1	2.85	< 0.1	0.116	< 0.1
F1 B-10	0.179	0.216	1.72	0.255	5.16	< 0.1	0.283	< 0.1
F1 B-11	0.198	0.193	1.94	0.287	5.87	< 0.1	0.193	< 0.1
F1 B-12	0.109	0.128	1.11	0.257	3.22	< 0.1	0.157	< 0.1
F1 B-13	< 0.1	< 0.1	1.64	0.148	3.60	< 0.1	0.117	< 0.1
F1 B-14	< 0.1	< 0.1	0.52	< 0.1	< 0.1	< 0.1	< 0.1	< 0.1
F1 B-15	0.16	0.174	2.15	0.615	3.59	< 0.1	0.386	< 0.1
F1 B-16	< 0.1	< 0.1	0.679	0.139	2.00	< 0.1	< 0.1	< 0.1
F1 B-17	0.551	0.305	6.57	2.91	12.4	< 0.1	2.36	0.233
F1 B-18	0.473	0.612	3.75	0.574	23.8	< 0.1	1.49	0.105
F1 B-19	0.187	0.189	1.32	0.464	6.17	< 0.1	0.311	< 0.1
F1 B-20	< 0.1	< 0.1	0.878	0.301	3.57	< 0.1	0.117	< 0.1
F1 B-21	< 0.1	< 0.1	0.767	0.107	2.09	< 0.1	< 0.1	< 0.1
F1 B-22	0.690	0.715	6.89	2.56	24	< 0.1	1.88	0.419
F1 B-23	0.170	0.242	2.18	0.718	7.68	< 0.1	0.554	< 0.1
F1 B-24	0.964	1.53	8.59	3.55	35.6	< 0.1	1.55	0.336
F1 B-25	1.30	2.14	15.8	4.83	49.4	0.103	3.85	0.720
F1 B-26	0.138	0.200	3.15	1.22	5.17	< 0.1	0.584	0.109
F1 B-27	0.166	0.244	2.14	0.423	9.76	< 0.1	0.589	< 0.1

F1 B-28	0.139	0.341	1.81	0.516	8.95	< 0.1	0.418	< 0.1
F1 B-29	0.113	0.281	1.45	0.317	8.12	< 0.1	0.500	< 0.1
F1 B-30	0.285	0.529	2.96	0.522	17.2	< 0.1	0.738	< 0.1
F1 B-31	< 0.1	0.210	1.35	0.429	7.16	< 0.1	0.382	< 0.1
F1 B-32	0.129	0.134	1.28	0.291	6.74	< 0.1	0.364	< 0.1
F1 B-33	0.126	0.260	1.52	0.349	10.4	< 0.1	0.477	< 0.1
F1 B-34	0.155	0.289	3.51	0.596	15.0	< 0.1	0.666	< 0.1
F1 B-38	0.160	0.230	3.25	0.761	11.6	< 0.1	0.705	< 0.1
F1 B-39	0.124	0.218	2.29	0.646	12.9	< 0.1	0.571	< 0.1
F1 B-40	0.132	0.268	2.06	0.210	13.9	< 0.1	0.633	< 0.1
F1 B-41	0.294	0.983	2.88	1.22	29.5	< 0.1	2.14	< 0.1
F1 B-42	0.168	0.607	1.54	0.484	19.4	< 0.1	1.14	< 0.1
F1 B-43	< 0.1	0.275	0.915	0.160	10.1	< 0.1	0.399	< 0.1
F1 B-45	0.362	0.634	4.75	2.31	28.7	< 0.1	1.55	0.156
F1 B-46	0.226	0.718	2.43	0.547	22.9	< 0.1	0.954	< 0.1
F1 B-47	0.547	1.11	3.68	1.28	33.1	< 0.1	1.11	0.114
F1 B-48	0.213	0.913	3.38	0.828	28.1	< 0.1	1.11	< 0.1
F1 B-49	< 0.1	0.277	0.840	0.148	8.71	< 0.1	0.295	< 0.1
F1 B-50	< 0.1	0.277	1.05	0.205	28.4	< 0.1	0.211	< 0.1

Table S33. Outdoor irrigation results of the test specimen F 1 B: heavy metal and trace element concentrations, part 2.

Leachate	Cu µg/l	Mo µg/l	Ni µg/l	Hg µg/l	Se µg/l	Tl µg/l	V µg/l	Zn µg/l
F1 B-1	3.77	4.02	1.93	< 0.01	3.19	< 0.1	61.2	7.27
F1 B-2	0.837	< 0.1	1.15	< 0.01	0.206	< 0.1	4.79	2.10
F1 B-3	1.17	< 0.1	0.950	< 0.01	0.316	< 0.1	9.25	1.97
F1 B-4	1.48	0.497	2.41	< 0.01	0.244	< 0.1	6.55	1.45
F1 B-5	8.81	0.496	1.34	< 0.01	0.324	< 0.1	6.58	3.71
F1 B-6	0.330	0.120	< 0.1	< 0.01	< 0.1	< 0.1	1.07	< 0.1
F1 B-7	1.30	0.457	< 0.1	< 0.01	< 0.1	< 0.1	1.01	1.90
F1 B-8	0.602	0.142	< 0.1	< 0.01	< 0.1	< 0.1	0.931	33.8
F1 B-9	0.253	0.286	< 0.1	< 0.01	< 0.1	< 0.1	0.506	< 0.1
F1 B-10	1.24	0.305	< 0.1	< 0.01	0.246	< 0.1	1.32	1.55
F1 B-11	0.808	0.334	< 0.1	< 0.01	0.394	< 0.1	0.885	3.03
F1 B-12	0.634	0.166	< 0.1	< 0.01	0.233	< 0.1	0.554	2.08
F1 B-13	0.822	0.157	< 0.1	< 0.01	0.298	< 0.1	0.251	3.57
F1 B-14	0.374	< 0.1	< 0.1	< 0.01	< 0.1	< 0.1	< 0.1	1.46
F1 B-15	1.57	0.301	1.21	< 0.01	0.175	< 0.1	0.855	7.76
F1 B-16	0.371	0.106	0.854	< 0.01	0.155	< 0.1	0.285	1.64
F1 B-17	9.16	0.612	1.38	< 0.01	0.387	< 0.1	1.81	28.0
F1 B-18	6.29	1.26	1.56	< 0.01	0.589	< 0.1	4.17	6.40
F1 B-19	1.24	0.396	0.380	< 0.01	0.115	< 0.1	1.31	2.84
F1 B-20	0.429	0.126	0.124	< 0.01	0.115	< 0.1	0.503	2.59
F1 B-21	0.136	< 0.1	< 0.1	< 0.01	< 0.1	< 0.1	0.458	1.40
F1 B-22	16.7	1.76	3.32	0.0154	1.02	< 0.1	3.83	26.0
F1 B-23	2.58	0.500	0.554	0.0106	0.191	< 0.1	1.94	6.10
F1 B-24	16.0	1.36	2.51	0.0159	0.760	< 0.1	4.94	23.0
F1 B-25	27.9	2.11	4.59	0.0139	0.841	< 0.1	14.4	49.6
F1 B-26	2.97	0.330	0.533	< 0.01	0.110	< 0.1	1.29	9.66
F1 B-27	1.47	0.297	0.489	< 0.01	0.168	< 0.1	1.62	14.3
F1 B-28	1.52	0.252	0.358	< 0.01	0.208	< 0.1	1.95	6.72

F1 B-29	1.09	0.222	0.434	< 0.01	0.138	< 0.1	1.57	5.04
F1 B-30	2.70	0.679	0.336	< 0.01	0.787	< 0.1	4.17	5.19
F1 B-31	1.01	0.297	0.189	< 0.01	0.184	< 0.1	1.22	4.22
F1 B-32	0.975	0.244	0.820	< 0.01	< 0.1	< 0.1	0.830	6.16
F1 B-33	2.04	0.283	0.290	< 0.01	0.109	< 0.1	1.76	4.37
F1 B-34	1.61	0.324	0.356	< 0.01	0.131	< 0.1	2.59	3.32
F1 B-38	2.62	0.480	0.575	< 0.01	0.176	< 0.1	1.70	11.1
F1 B-39	1.90	**22.2**	1.80	< 0.01	< 0.1	< 0.1	1.66	5.21
F1 B-40	1.37	**6.96**	2.99	< 0.01	0.265	< 0.1	2.44	2.56
F1 B-41	4.00	**5.93**	4.37	< 0.01	0.342	< 0.1	7.74	10.8
F1 B-42	1.30	**2.76**	1.93	< 0.01	0.180	< 0.1	5.56	4.50
F1 B-43	0.443	**1.47**	0.620	< 0.01	< 0.1	< 0.1	2.28	0.826
F1 B-45	4.43	**363**	1.11	< 0.01	0.437	< 0.1	4.27	16.1
F1 B-46	1.33	< 0.1	0.143	< 0.01	0.342	< 0.1	5.95	6.61
F1 B-47	3.42	0.147	0.667	< 0.01	1.04	< 0.1	5.57	8.83
F1 B-48	1.67	0.947	0.722	< 0.01	0.191	< 0.1	6.49	6.35
F1 B-49	0.452	0.371	0.348	< 0.01	0.110	< 0.1	1.78	< 1
F1 B-50	0.517	1.00	0.430	< 0.01	< 0.1	< 0.1	1.28	1.20

Bold: Significantly increased blank values for molybdenum, probably through contaminations during analysis or by particle deposits, led to likewise increased concentrations for the test specimens.

Table 34. Outdoor irrigation results of the test specimen F 1 C: leachate amount, pH value, electrical conductivity and concentrations of sodium, potassium, calcium, chloride, and sulfate.

Date of Sampling	Leachate Label	Amount L/m²	pH -	EC µS/cm	Na mg/L	K mg/L	Ca mg/L	Cl^- mg/L	SO_4^{2-} mg/L
31.01.2019	F1 C-14	2.26	7.2	56	4	2	6.8	2.4	0.8
07.02.2019	F1 C-15	5.40	9.9	460	5.75	2.2	11.7	2.5	2.4
14.02.2019	F1 C-16	25.3	9.7	124	12	24	2.5	2.8	1.5
21.02.2019	F1 C-17	0.27	7.7	367	38	75	2.4	2	18.9
28.02.2019	F1 C-18	0.53	8.9	1279	27.3	302	1.6	2	15.4
07.03.2019	F1 C-19	18.9	8.7	186	18.6	47.2	1.4	1	2.1
14.03.2019	F1 C-20	51.2	8.6	80	7	13.6	2.5	3.3	0.9
21.03.2019	F1 C-21	35.8	7.5	54	3.3	7.1	4.2	1.1	0.5
28.03.2019	F1 C-22	0.42	6.9	194	334	40.4	3.6	51.5	9.9
04.04.2019	F1 C-23	5.94	7.1	81	6.4	12.8	2	1.6	1
11.04.2019	F1 C-24	0.68	6.8	311	29.4	59.1	2.6	12.2	12.9
18.04.2019	F1 C-25	0.29	7.2	493	59.3	121.2	3.4	41.3	9.7
25.04.2019	F1 C-26	5.43	7.0	75	4.2	11.7	3.5	< 0.1	1.1
02.05.2019	F1 C-27	7.43	7.3	106	9.3	20.4	2.3	2.1	0.6
09.05.2019	F1 C-28	13.8	7.4	119	10.1	25.1	2.3	0.6	1.4
16.05.2019	F1 C-29	7.20	7.3	90	5.8	16.8	2.4	1.5	3.1
23.05.2019	F1 C-30	7.57	7.5	135	11.7	30.4	2.7	3.1	2.9
30.05.2019	F1 C-31	11.1	7.5	52	3.6	10.6	3.3	3.7	1.7
06.06.2019	F1 C-32	21.8	7.0	35	1.7	4.6	3.7	< 0.1	0.6
13.06.2019	F1 C-33	8.62	7.0	62	4.7	9.8	3.2	< 0.1	1.5
20.06.2019	F1 C-34	8.09	7.3	73	4.5	10.1	5.3	1	0.3
18.07.2019	F1 C-38	7.19	7.3	49	1.9	5.0	4.6	1.8	1
25.07.2019	F1 C-39	6.11	n. d.	n. d.	2.5	5.5	4	6.5	< 0.1
01.08.2019	F1 C-40	19.3	n. b	n. d.	2.5	6.3	6.3	< 0.1	1
08.08.2019	F1 C-41	2.94	7.2	126	15	23.7	2.8	2.3	3.4
15.08.2019	F1 C-42	11.4	7.2	79	8.6	16.3	2.8	0.5	1.7

22.08.2019	F1 C-43	16.4	7.1	50	3.6	8.9	3.2	1.2	0.5
05.09.2019	F1 C-45	3.71	7.0	95	6.4	13.3	7.0	2.4	4.6
12.09.2019	F1 C-46	7.63	7.3	94	8.3	13.7	3.6	3.2	2
19.09.2019	F1 C-47	1.17	6.6	146	9.9	21.5	4.5	38.1	7
26.09.2019	F1 C-48	7.29	7.1	90	7.1	15.2	3.6	0.5	1.9
03.10.2019	F1 C-49	25.4	7.1	45	3	5.80	3.3	0.8	0.9
10.10.2019	F1 C-50	28.7	7.5	34	3	5.40	4.5	0.8	0.6

Due to the absence of run-off, no samples were taken on the following dates: 27.06.2019, 04.07.2019, 11.07.2019, 29.08.2019; n. d. = not determined.

Table S35. Outdoor irrigation results of the test specimen F 1 C: heavy metal and trace element concentrations, part 1.

Leachate	Sb µg/l	As µg/l	Ba µg/l	Pb µg/l	B µg/l	Cd µg/l	Cr µg/l	Co µg/l
F1 C-14	0.120	0.178	10.5	0.785	3.96	< 0.1	0.617	0.503
F1 C-15	0.169	0.377	3.07	0.426	5.95	< 0.1	0.982	< 0.1
F1 C-16	0.101	0.403	1.49	0.370	7.60	< 0.1	0.677	< 0.1
F1 C-17	0.525	1.03	5.44	1.40	23.5	< 0.1	3.45	0.439
F1 C-18	0.724	2.79	3.13	1.86	60.4	< 0.1	7.55	0.273
F1 C-19	0.196	0.542	1.39	0.480	11.4	< 0.1	0.924	< 0.1
F1 C-20	< 0.1	0.230	1.37	0.469	6.13	< 0.1	0.348	< 0.1
F1 C-21	< 0.1	< 0.1	0.798	< 0.1	3.97	< 0.1	0.137	< 0.1
F1 C-22	0.490	1.58	7.51	4.44	25.3	0.135	1.51	0.448
F1 C-23	0.157	0.431	2.14	0.933	7.51	< 0.1	0.532	< 0.1
F1 C-24	0.929	3.57	5.16	3.42	41.5	0.103	1.71	0.518
F1 C-25	0.975	5.72	12.1	4.94	63.9	0.125	3.70	0.803
F1 C-26	0.185	0.294	5.17	2.69	4.80	< 0.1	1.73	0.358
F1 C-27	0.164	0.378	1.70	0.732	10.0	< 0.1	0.639	< 0.1
F1 C-28	0.168	0.663	1.92	0.857	9.69	< 0.1	0.652	< 0.1
F1 C-29	0.113	0.410	1.08	0.317	7.19	< 0.1	0.528	< 0.1
F1 C-30	0.271	0.920	2.83	0.600	16.6	< 0.1	0.976	< 0.1
F1 C-31	0.121	0.271	2.62	1.07	7.23	< 0.1	0.649	0.120
F1 C-32	0.129	0.146	1.30	0.261	5.53	< 0.1	0.339	< 0.1
F1 C-33	0.115	0.361	1.40	0.386	9.11	< 0.1	0.517	< 0.1
F1 C-34	0.159	0.494	4.37	1.28	12.1	< 0.1	0.839	0.132
F1 C-38	0.215	0.272	5.05	1.99	9.64	< 0.1	2.91	0.525
F1 C-39	0.115	0.235	2.54	0.750	11.7	< 0.1	1.00	0.112
F1 C-40	0.128	0.244	1.57	0.121	11.9	< 0.1	0.897	< 0.1
F1 C-41	0.235	0.894	2.85	1.56	22.1	< 0.1	2.61	0.123
F1 C-42	0.145	0.683	1.54	0.587	15.3	< 0.1	1.06	< 0.1
F1 C-43	< 0.1	0.271	0.962	0.184	8.38	< 0.1	0.486	< 0.1
F1 C-45	0.302	0.524	4.33	2.13	21.7	< 0.1	2.12	0.307
F1 C-46	0.188	0.681	1.72	0.611	18.3	< 0.1	0.920	< 0.1
F1 C-47	0.437	0.885	2.65	0.789	23.6	< 0.1	1.75	0.104
F1 C-48	0.147	0.806	1.46	0.398	20.2	< 0.1	1.06	< 0.1
F1 C-49	< 0.1	0.254	0.717	0.111	7.67	< 0.1	0.358	< 0.1
F1 C-50	< 0.1	0.240	0.907	< 0.1	23.6	< 0.1	0.321	< 0.1

Table S36. Outdoor irrigation results of the test specimen F 1 C: heavy metal and trace element concentrations, part 2.

Leachate	Cu µg/l	Mo µg/l	Ni µg/l	Hg µg/l	Se µg/l	Tl µg/l	V µg/l	Zn µg/l

F1 C-14	6.13	0.249	0.625	< 0.01	< 0.1	< 0.1	0.613	22.0
F1 C-15	1.70	0.689	1.12	< 0.01	0.306	< 0.1	3.26	3.52
F1 C-16	0.531	0.394	0.877	< 0.01	0.296	< 0.1	4.74	1.87
F1 C-17	8.68	1.27	2.37	< 0.01	0.934	< 0.1	9.68	12.0
F1 C-18	4.83	3.23	5.17	< 0.01	1.35	< 0.1	20.6	5.63
F1 C-19	1.20	0.542	0.816	< 0.01	0.201	< 0.1	4.08	2.56
F1 C-20	0.679	0.266	0.199	< 0.01	< 0.1	< 0.1	1.46	4.42
F1 C-21	0.146	0.206	0.108	< 0.01	0.117	< 0.1	0.870	< 1
F1 C-22	14.4	1.16	2.14	0.0168	0.968	< 0.1	6.05	45.8
F1 C-23	2.51	0.346	0.734	< 0.01	0.157	< 0.1	2.11	7.93
F1 C-24	17.1	1.47	4.02	0.0129	1.06	< 0.1	10.8	26.1
F1 C-25	18.7	1.99	5.97	< 0.01	0.669	< 0.1	27.3	41.5
F1 C-26	5.05	1.64	11.2	< 0.01	< 0.1	< 0.1	1.69	24.0
F1 C-27	1.63	0.637	0.909	< 0.01	0.197	< 0.1	1.85	7.35
F1 C-28	2.00	0.407	0.730	< 0.01	0.263	< 0.1	3.05	8.31
F1 C-29	1.05	0.248	0.213	< 0.01	0.106	< 0.1	1.92	4.28
F1 C-30	2.72	0.681	0.491	< 0.01	0.795	< 0.1	4.82	5.88
F1 C-31	3.14	0.344	0.820	< 0.01	0.165	< 0.1	1.52	16.4
F1 C-32	1.16	0.259	0.474	< 0.01	< 0.1	< 0.1	0.955	4.07
F1 C-33	2.10	0.280	0.389	< 0.01	0.140	< 0.1	2.32	5.17
F1 C-34	2.43	0.321	0.788	< 0.01	0.132	< 0.1	3.15	9.67
F1 C-38	12.7	3.00	33.6	< 0.01	0.150	< 0.1	1.32	33.4
F1 C-39	1.98	**19.6**	4.14	< 0.01	< 0.1	< 0.1	1.46	6.71
F1 C-40	0.922	**6.65**	3.52	< 0.01	0.194	< 0.1	1.55	1.64
F1 C-41	3.53	**4.53**	4.21	0.0121	0.267	< 0.1	5.58	13.0
F1 C-42	1.52	**2.98**	2.05	< 0.01	0.139	< 0.1	4.94	3.07
F1 C-43	0.491	**1.44**	0.991	< 0.01	< 0.1	< 0.1	1.73	1.88
F1 C-45	4.83	**87.4**	6.76	< 0.01	0.316	< 0.1	3.14	16.5
F1 C-46	1.25	< 0.1	0.858	< 0.01	0.304	< 0.1	5.01	4.71
F1 C-47	2.62	0.25	1.51	< 0.01	1.01	< 0.1	4.59	9.75
F1 C-48	2.16	0.885	1.33	< 0.01	0.159	< 0.1	4.92	6.20
F1 C-49	0.417	0.458	0.974	< 0.01	0.115	< 0.1	1.61	< 1
F1 C-50	0.377	1.00	0.746	< 0.01	< 0.1	< 0.1	1.37	< 1

Bold: Significantly increased blank values for molybdenum, probably through contaminations during analysis or by particle deposits, led to likewise increased concentrations for the test specimens.

Table S37. Outdoor irrigation results of the test specimen F 1g A: leachate amount, pH value, electrical conductivity and concentrations of sodium, potassium, calcium, chloride, and sulfate.

Date of Sampling	Leachate Label	Amount L/m²	pH -	EC µS/cm	Na mg/L	K mg/L	Ca mg/L	Cl^- mg/L	SO_4^{2-} mg/L
25.10.2018	F1g A-1	3.15	10.0	1123	103	239	1.7	4.2	30.5
01.11.2018	F1g A-2	17.6	8.6	172	15.3	44.7	1.8	0.6	4.4
08.11.2018	F1g A-3	2.00	8.4	197	19.2	46.4	2.9	0.4	8.1
15.11.2018	F1g A-4	7.11	8.5	200	16.6	50.3	2.1	0.7	2.4
29.11.2018	F1g A-5	6.70	9.1	169	16.9	47.6	1.3	0.2	4.9
06.12.2018	F1g A-6	25.5	7.6	59	4	11	3.8	0.3	< 0.1
13.12.2018	F1g A-7	26.0	7.6	114	13	10	6.9	1.7	2.7
20.12.2018	F1g A-8	4.63	7.7	66	7	10	3.5	0.4	0.2
27.12.2018	F1g A-9	32.5	7.2	44	2	5	4.6	0.5	< 0.1
03.01.2019	F1g A-10	11.8	7.3	79	6	9	6.8	1.7	1.3

10.01.2019	F1g A-11	22.6	7.1	75	6	6	6.3	2.8	1.7
17.01.2019	F1g A-12	35.4	7.0	50	3	4	5.4	0.9	< 0.1
24.01.2019	F1g A-13	2.29	7.3	63	4	4	7.1	3	2.9
31.01.2019	F1g A-14	2.46	6.9	10	< 0.1	< 0.1	1.3	0.3	< 0.1
07.02.2019	F1g A-15	6.13	7.1	183	8	7	7.1	5.8	0.9
14.02.2019	F1g A-16	25.3	7.4	42	3	3	4.7	1.7	1
21.02.2019	F1g A-17	0.29	6.8	269	8	27	17.2	5.8	17.7
28.02.2019	F1g A-18	0.62	7.5	179	19	19	10.5	6.2	9.9
07.03.2019	F1g A-19	24.6	7.2	80	4	8.2	4	1.3	1.6
14.03.2019	F1g A-20	51.7	7.5	74	2.6	3.8	4.3	2.4	0.8
21.03.2019	F1g A-21	34.8	7.3	44	1.6	2.6	5.6	1.5	0.4
28.03.2019	F1g A-22	0.20	7.0	178	28.7	22.3	15.8	37.8	18
04.04.2019	F1g A-23	5.10	7.1	50	4.3	6.6	3	0.6	2.7
11.04.2019	F1g A-24	0.44	6.9	192	16.8	27.3	9.2	6.5	16.3
18.04.2019	F1g A-25	0.09	7.3	290	35.7	27.3	9.2	3.4	21.7
25.04.2019	F1g A-26	7.43	7.0	83	2.1	6.4	4.5	0.2	1
02.05.2019	F1g A-27	8.59	7.1	73	3.8	7.3	5.9	3.2	1.1
09.05.2019	F1g A-28	14.2	7.2	73	4.9	9.6	4.8	0.7	1.8
16.05.2019	F1g A-29	7.97	7.2	71	3.2	8.8	4.7	2.7	0.8
23.05.2019	F1g A-30	8.26	7.5	32	6.6	15.4	6.4	1.9	5
30.05.2019	F1g A-31	12.2	7.3	41	2.5	7.9	4.6	0.5	1.3
06.06.2019	F1g A-32	23.8	7.1	33	1.1	3.5	4.4	0.3	1.3
13.06.2019	F1g A-33	9.41	6.9	50	2.5	5.2	5.6	0.2	2
20.06.2019	F1g A-34	9.11	7.2	56	2.4	5.6	7.8	0.8	3.8
18.07.2019	F1g A-38	7.91	7	59	n. d.	n. d.	n. d.	0.1	0.4
25.07.2019	F1g A-39	7.84	n. d.	n. d.	2.0	4.2	4.4	1.5	< 0.1
01.08.2019	F1g A-40	19.7	n. d.	n. d.	2.7	6.1	7.5	0.2	0.6
08.08.2019	F1g A-41	3.20	7.2	127	17.8	24.4	3.1	1.5	5.5
15.08.2019	F1g A-42	12.5	7.1	71	7.5	15.9	3.3	2.7	2.8
22.08.2019	F1g A-43	19.4	7.2	55	2.7	8.7	3.7	0.5	0.5
05.09.2019	F1g A-45	3.91	7.1	93	8.3	18.0	6.9	3.6	3.9
12.09.2019	F1g A-46	8.66	7.3	95	7.1	13.4	4.7	3	2.8
19.09.2019	F1g A-47	1.47	4.1	202	10.7	23.5	5.5	54.4	5.3
26.09.2019	F1g A-48	8.42	7.4	59	7.2	16.4	3.7	0.5	2.2
03.10.2019	F1g A-49	25.4	7.5	41	2.6	6.5	3.4	0.6	0.5
10.10.2019	F1g A-50	36.5	7.5	46	3.0	6.2	4.4	0.6	0.6

Due to the absence of run-off, no samples were taken on the following dates: 18.10.2018, 22.11.2018, 27.06.2019, 04.07.2019, 11.07.2019, 29.08.2019; n. d. = not determined.

Table S38. Outdoor irrigation results of the test specimen F 1g A: heavy metal and trace element concentrations, part 1.

Leachate	Sb µg/l	As µg/l	Ba µg/l	Pb µg/l	B µg/l	Cd µg/l	Cr µg/l	Co µg/l
F1g A-1	1.35	8.04	4.84	1.98	147	< 0.1	8.02	0.709
F1g A-2	0.195	0.736	1.47	0.317	14.7	< 0.1	1.52	< 0.1
F1g A-3	0.332	0.982	2.10	0.227	22.3	< 0.1	1.72	< 0.1
F1g A-4	0.201	0.872	0.867	0.149	19.5	< 0.1	1.18	< 0.1
F1g A-5	0.327	1.04	1.56	0.570	20.6	< 0.1	1.09	0.108
F1g A-6	< 0.1	0.158	0.73	0.117	5.02	< 0.1	0.346	< 0.1
F1g A-7	< 0.1	< 0.1	0.822	0.135	4.51	< 0.1	0.192	< 0.1
F1g A-8	0.111	0.211	1.39	0.432	4.98	< 0.1	0.531	< 0.1
F1g A-9	< 0.1	< 0.1	0.580	< 0.1	2.05	< 0.1	0.211	< 0.1

F1g A-10	0.168	0.284	1.40	0.179	5.64	< 0.1	0.419	< 0.1
F1g A-11	0.194	0.254	1.36	0.308	5.03	< 0.1	0.288	< 0.1
F1g A-12	< 0.1	0.148	0.717	0.243	2.58	< 0.1	0.193	< 0.1
F1g A-13	0.103	< 0.1	0.988	0.137	4.70	< 0.1	0.268	0.101
F1g A-14	< 0.1	< 0.1	2.25	0.358	< 0.1	< 0.1	< 0.1	< 0.1
F1g A-15	0.168	0.250	1.51	0.457	4.08	< 0.1	0.552	0.106
F1g A-16	< 0.1	< 0.1	0.612	0.157	2.10	< 0.1	0.214	< 0.1
F1g A-17	0.707	1.15	6.06	1.42	24.0	< 0.1	4.65	0.249
F1g A-18	0.498	0.810	2.94	0.319	27.7	< 0.1	1.78	0.119
F1g A-19	0.148	0.201	1.26	0.580	5.23	< 0.1	0.408	< 0.1
F1g A-20	< 0.1	< 0.1	0.773	0.307	2.83	< 0.1	0.142	< 0.1
F1g A-21	< 0.1	< 0.1	0.644	< 0.1	2.10	< 0.1	0.155	< 0.1
F1g A-22	0.772	0.798	8.61	3.12	26.4	< 0.1	3.02	0.49
F1g A-23	0.183	0.265	2.35	1.23	6.56	< 0.1	2.39	0.373
F1g A-24	0.896	1.44	6.75	2.15	32.2	< 0.1	1.70	0.350
F1g A-25	1.38	2.33	16.3	5.50	50.1	0.106	13.2	1.49
F1g A-26	0.155	0.220	3.53	1.72	4.13	< 0.1	1.41	0.296
F1g A-27	0.157	0.275	1.69	0.41	9.54	< 0.1	0.808	< 0.1
F1g A-28	0.126	0.333	1.37	0.413	8.30	< 0.1	0.493	< 0.1
F1g A-29	0.117	0.313	2.10	0.316	7.75	< 0.1	0.723	< 0.1
F1g A-30	0.280	0.584	2.70	0.412	16.4	< 0.1	0.747	< 0.1
F1g A-31	0.101	0.241	1.34	0.409	6.76	< 0.1	0.485	< 0.1
F1g A-32	0.113	0.151	0.990	0.234	5.83	< 0.1	0.373	< 0.1
F1g A-33	0.138	0.268	1.44	0.310	9.93	< 0.1	0.933	< 0.1
F1g A-34	0.154	0.273	3.77	0.362	12.7	< 0.1	0.967	< 0.1
F1g A-38	0.209	0.249	4.29	1.52	12.4	< 0.1	23.3	3.17
F1g A-39	0.127	0.206	2.03	0.673	13.6	< 0.1	4.42	0.267
F1g A-40	0.134	0.317	1.84	0.217	14.7	< 0.1	1.29	0.179
F1g A-41	0.327	1.14	3.29	2.08	30.6	< 0.1	6.85	0.225
F1g A-42	0.143	0.651	1.33	0.397	18.0	< 0.1	2.08	0.172
F1g A-43	< 0.1	0.272	0.992	0.0793	9.28	< 0.1	0.881	< 0.1
F1g A-45	0.362	0.636	4.89	1.93	27.7	< 0.1	11.1	0.993
F1g A-46	0.214	0.776	2.1	0.652	22.2	< 0.1	1.76	0.111
F1g A-47	0.586	0.954	4.58	1.92	28.8	< 0.1	22.1	0.733
F1g A-48	0.220	0.963	2.31	0.734	24.1	< 0.1	4.06	< 0.1
F1g A-49	< 0.1	0.264	0.737	0.122	7.12	< 0.1	0.430	< 0.1
F1g A-50	< 0.1	0.290	0.903	0.225	19.7	< 0.1	0.343	< 0.1

Table S39. Outdoor irrigation results of the test specimen F 1g A: heavy metal and trace element concentrations, part 2.

Leachate	Cu µg/l	Mo µg/l	Ni µg/l	Hg µg/l	Se µg/l	Tl µg/l	V µg/l	Zn µg/l
F1g A-1	7.28	2.94	2.34	0.0113	2.60	< 0.1	64.2	16.0
F1g A-2	1.33	< 0.1	1.32	< 0.01	0.236	< 0.1	5.75	12.0
F1g A-3	1.82	< 0.1	1.16	< 0.01	0.268	< 0.1	7.53	3.46
F1g A-4	1.87	0.698	2.63	< 0.01	0.260	< 0.1	5.9	1.06
F1g A-5	1.90	0.718	3.70	< 0.01	0.321	< 0.1	7.4	3.76
F1g A-6	0.483	0.190	< 0.1	< 0.01	< 0.1	< 0.1	1.56	1.20
F1g A-7	0.571	0.337	0.442	< 0.01	< 0.1	< 0.1	0.577	1.74
F1g A-8	1.08	0.231	0.708	< 0.01	< 0.1	< 0.1	1.8	4.42
F1g A-9	0.381	0.129	< 0.1	< 0.01	< 0.1	< 0.1	0.805	1.37
F1g A-10	0.774	0.185	< 0.1	< 0.01	0.227	< 0.1	1.91	1.48

F1g A-11	0.857	0.294	1.6	< 0.01	0.398	< 0.1	1.23	2.22
F1g A-12	0.603	0.147	0.483	< 0.01	0.212	< 0.1	0.704	2.15
F1g A-13	0.687	0.468	1.84	< 0.01	0.286	< 0.1	0.385	3.91
F1g A-14	1.48	< 0.1	< 0.1	< 0.01	< 0.1	< 0.1	< 0.1	13.2
F1g A-15	1.44	0.372	2.52	< 0.01	0.172	< 0.1	1.21	6.83
F1g A-16	0.342	0.140	1.20	< 0.01	< 0.1	< 0.1	0.549	1.16
F1g A-17	9.46	1.94	2.01	0.0123	0.859	< 0.1	9.94	15.2
F1g A-18	5.44	1.87	5.47	< 0.01	0.545	< 0.1	5.73	5.05
F1g A-19	1.17	0.393	0.918	< 0.01	0.148	< 0.1	1.35	4.25
F1g A-20	0.356	0.135	0.283	< 0.01	< 0.1	< 0.1	0.451	1.87
F1g A-21	0.150	0.120	0.170	< 0.01	< 0.1	< 0.1	0.642	< 1
F1g A-22	18.1	1.89	4.05	0.0137	1.12	< 0.1	5.38	33
F1g A-23	2.98	1.64	15.4	< 0.01	0.180	< 0.1	1.97	7.94
F1g A-24	14.8	2.35	6.34	0.0105	0.972	< 0.1	4.87	15.3
F1g A-25	29.1	6.20	52.5	0.0144	0.731	< 0.1	12.6	44.9
F1g A-26	3.98	0.484	11.7	< 0.01	0.115	< 0.1	1.27	13.3
F1g A-27	1.55	0.455	2.56	< 0.01	0.122	< 0.1	1.63	5.54
F1g A-28	1.28	0.276	1.31	< 0.01	0.189	< 0.1	1.94	5.08
F1g A-29	1.69	0.382	1.79	< 0.01	0.181	< 0.1	1.63	6.09
F1g A-30	2.21	1.01	1.70	< 0.01	0.717	< 0.1	3.55	4.79
F1g A-31	1.07	0.406	0.766	< 0.01	0.175	< 0.1	1.14	4.87
F1g A-32	0.996	0.271	0.642	< 0.01	0.112	< 0.1	0.903	2.80
F1g A-33	1.40	0.369	0.749	< 0.01	0.149	< 0.1	2.14	3.93
F1g A-34	1.34	0.395	0.623	< 0.01	0.142	< 0.1	2.80	2.10
F1g A-38	10.4	**9.34**	25.2	< 0.01	0.194	< 0.1	1.77	13.8
F1g A-39	2.37	**15.8**	13.8	< 0.01	< 0.1	< 0.1	1.74	5.73
F1g A-40	1.23	**8.08**	10.7	< 0.01	0.215	< 0.1	2.61	1.53
F1g A-41	4.12	**6.95**	7.02	< 0.01	0.367	< 0.1	7.96	14.9
F1g A-42	1.08	**3.28**	5.14	< 0.01	0.206	< 0.1	4.94	1.65
F1g A-43	0.339	**1.91**	1.07	< 0.01	< 0.1	< 0.1	2.08	< 1
F1g A-45	6.68	**67.5**	34.6	< 0.01	0.425	< 0.1	4.12	22.3
F1g A-46	1.40	0.564	7.65	< 0.01	0.329	< 0.1	5.94	5.47
F1g A-47	4.65	0.424	12.0	< 0.01	0.893	< 0.1	4.43	23.0
F1g A-48	1.68	1.52	2.54	< 0.01	0.231	< 0.1	6.36	5.81
F1g A-49	0.387	0.579	1.73	< 0.01	0.128	< 0.1	1.61	< 1
F1g A-50	0.452	1.00	0.763	< 0.01	0.112	< 0.1	1.21	< 1

Bold: Significantly increased blank values for molybdenum, probably through contaminations during analysis or by particle deposits, led to likewise increased concentrations for the test specimens.

Table S40. Outdoor irrigation results of the test specimen F 1g B: leachate amount, pH value, electrical conductivity and concentrations of sodium, potassium, calcium, chloride, and sulfate.

Date of Sampling	Leachate Label	Amount L/m²	pH -	EC µS/cm	Na mg/L	K mg/L	Ca mg/L	Cl^- mg/L	SO_4^{2-} mg/L
25.10.2018	F1g B-1	2.80	9.9	966	83.2	212.3	2.6	4.5	31.3
01.11.2018	F1g B-2	15.22	9.3	140	11.5	36.2	2.7	0.6	2.4
08.11.2018	F1g B-3	1.72	8.0	147.1	14.1	30.4	4.7	0.2	1.8
15.11.2018	F1g B-4	6.23	8.3	205.7	17.1	51.7	2.6	0.8	2.5
29.11.2018	F1g B-5	6.32	8.2	147.9	13.4	40.4	1.6	0.3	3.5
06.12.2018	F1g B-6	25.4	7.1	52	3	8	4.1	0.3	0.6
13.12.2018	F1g B-7*	26.0	7.2	54	4	5	4.7	1.7	< 0.1
20.12.2018	F1g B-8	5.47	7.3	67	7	9	3.9	0.7	1.1

27.12.2018	F1g B-9	29.3	7.1	41	2	4	4.6	< 0.1	1.3
03.01.2019	F1g B-10	11.6	7.3	69	5	7	6.2	1.4	1.5
10.01.2019	F1g B-11	22.4	7.2	69	6	5	6.1	2.8	1.5
17.01.2019	F1g B-12	34.5	7.2	47	3	3	5.4	0.9	< 0.1
24.01.2019	F1g B-13	2.31	7.6	62	3	3	7.1	2.8	1.4
31.01.2019	F1g B-14	2.63	6.9	7	< 0.1	< 0.1	1.1	< 0.1	< 0.1
07.02.2019	F1g B-15	5.66	7.0	82	8	6	6.6	5.3	1.1
14.02.2019	F1g B-16	25.3	7.3	43	3	2	6	1.8	< 0.1
21.02.2019	F1g B-17	0.16	5.3	204	8	16	10.9	5.8	13
28.02.2019	F1g B-18	0.37	7.5	164	18	21	7.4	5	10
07.03.2019	F1g B-19	21.6	7.3	80	3.9	8.6	4.2	1.2	1.5
14.03.2019	F1g B-20	47.8	7.3	60	2.4	2.7	4.5	3.7	0.9
21.03.2019	F1g B-21	32.0	7.2	43	1.4	2.2	5.8	1	0.4
28.03.2019	F1g B-22	0.30	7.0	265	29.4	24.3	11.1	48.3	16.5
04.04.2019	F1g B-23	5.44	7.1	69	5.2	7.3	4.8	3.4	2.9
11.04.2019	F1g B-24	0.53	6.9	214	16.5	31.1	9.8	11	18.4
18.04.2019	F1g B-25	0.24	7.4	455	43.2	66.8	11.3	33	30.7
25.04.2019	F1g B-26	5.28	6.9	71	3.4	8	5.2	0.2	2.1
02.05.2019	F1g B-27	7.58	7.1	78	3.8	7.9	5.5	3.8	0.8
09.05.2019	F1g B-28	13.4	7.1	70	4.4	8.9	4.7	1.1	0.9
16.05.2019	F1g B-29	7.68	7.2	61	2.5	7	4.7	1.5	1
23.05.2019	F1g B-30	7.90	7.4	99	5.2	13.1	6.6	1.1	2.7
30.05.2019	F1g B-31	11.8	7.2	43	1.9	5.8	5.0	2.1	1
06.06.2019	F1g B-32	21.3	7.1	34	0.8	2.7	4.9	< 0.1	1.3
13.06.2019	F1g B-33	8.54	6.9	48	2.2	4.9	5.8	0.4	2.3
20.06.2019	F1g B-34	6.84	7.2	51	1.8	4.6	7.3	0.6	2
18.07.2019	F1g B-38	8.00	6.9	51	1.9	3.8	7.2	2.7	5
25.07.2019	F1g B-39	6.39	n. d.	n. d.	1.8	3.8	4.9	2.0	< 0.1
01.08.2019	F1g B-40	19.6	n. d.	n. d.	2.6	6.2	7.4	0.2	1.7
08.08.2019	F1g B-41	2.35	7.2	131	18.6	29.9	3.1	5.7	7.4
15.08.2019	F1g B-42	10.5	7.2	49	7.4	17.8	3.7	2.2	3.2
22.08.2019	F1g B-43	16.8	7.2	37	3.2	9.3	3.3	0.8	0.5
05.09.2019	F1g B-45	3.5	7.2	101	7.2	16.5	7.7	9.6	6
12.09.2019	F1g B-46	7.8	7.3	96	6.4	12.6	5.2	7.4	3.5
19.09.2019	F1g B-47	1.19	3.1	457	10	24.3	5.6	81.2	6.9
26.09.2019	F1g B-48	7.37	7.3	81	6.5	16.3	4.2	0.7	2
03.10.2019	F1g B-49	25.4	7.2	48	3.7	9.7	3.7	0.6	1.6
10.10.2019	F1g B-50	32.8	7.4	49	2.8	6.3	5.0	0.9	0.4

Due to the absence of run-off, no samples were taken on the following dates: 18.10.2018, 22.11.2018, 27.06.2019, 04.07.2019, 11.07.2019, 29.08.2019; n. d. = not determined .

Table S41. Outdoor irrigation results of the test specimen F 1g B: heavy metal and trace element concentrations, part 1.

Leachate	Sb µg/l	As µg/l	Ba µg/l	Pb µg/l	B µg/l	Cd µg/l	Cr µg/l	Co µg/l
F1g B-1	1.11	4.69	2.89	0.481	99.5	< 0.1	7.18	0.348
F1g B-2	0.160	0.442	1.88	0.121	10.5	< 0.1	1.09	< 0.1
F1g B-3	0.194	0.369	3.51	< 0.1	11.6	< 0.1	0.937	< 0.1
F1g B-4	0.166	0.818	1.24	0.142	18.6	< 0.1	0.960	< 0.1
F1g B-5	0.271	0.787	2.10	0.317	15.5	< 0.1	0.661	< 0.1
F1g B-6	< 0.1	< 0.1	0.678	< 0.1	3.50	< 0.1	0.212	< 0.1
F1g B-7*	< 0.1	< 0.1	0.822	0.135	4.51	< 0.1	0.192	< 0.1

F1g B-8	0.101	0.134	1.44	0.330	4.45	< 0.1	0.301	< 0.1
F1g B-9	< 0.1	< 0.1	0.684	< 0.1	2.02	< 0.1	0.109	< 0.1
F1g B-10	0.159	0.228	2.63	0.244	4.58	< 0.1	0.237	< 0.1
F1g B-11	0.179	0.191	1.66	0.637	4.95	< 0.1	0.184	< 0.1
F1g B-12	0.104	0.122	1.04	0.287	2.18	< 0.1	0.144	< 0.1
F1g B-13	< 0.1	< 0.1	1.22	0.128	3.94	< 0.1	0.130	< 0.1
F1g B-14	< 0.1	< 0.1	0.52	< 0.1	< 0.1	< 0.1	< 0.1	< 0.1
F1g B-15	0.157	0.158	2.23	0.389	3.43	< 0.1	0.390	< 0.1
F1g B-16	< 0.1	< 0.1	0.807	0.137	1.85	< 0.1	0.126	< 0.1
F1g B-17	0.554	0.559	6.12	2.85	17.3	< 0.1	2.15	0.257
F1g B-18	0.449	0.488	3.43	0.495	21.9	< 0.1	1.15	0.257
F1g B-19	0.155	0.196	1.33	0.336	5.25	< 0.1	0.335	< 0.1
F1g B-20	< 0.1	< 0.1	1.10	0.591	4.16	< 0.1	0.255	< 0.1
F1g B-21	< 0.1	< 0.1	0.873	< 0.1	2.41	< 0.1	0.141	< 0.1
F1g B-22	0.640	0.731	7.89	2.14	25.9	< 0.1	1.62	0.676
F1g B-23	0.165	0.254	2.17	0.518	6.86	< 0.1	0.533	< 0.1
F1g B-24	1.02	1.63	8.42	3.48	34.7	< 0.1	1.71	0.391
F1g B-25	1.52	2.74	12.9	2.84	62.6	< 0.1	3.14	0.541
F1g B-26	0.204	0.289	3.09	1.49	5.81	< 0.1	0.775	0.182
F1g B-27	0.162	0.233	2.13	0.533	8.88	< 0.1	0.886	< 0.1
F1g B-28	0.131	0.324	1.40	0.256	7.80	< 0.1	0.329	< 0.1
F1g B-29	< 0.1	0.225	1.64	0.246	6.71	< 0.1	0.406	< 0.1
F1g B-30	0.279	0.458	3.46	0.441	15.5	< 0.1	0.688	< 0.1
F1g B-31	< 0.1	0.218	1.44	0.326	6.58	< 0.1	0.450	< 0.1
F1g B-32	0.128	0.123	1.14	0.230	5.42	< 0.1	0.337	< 0.1
F1g B-33	0.138	0.208	1.62	0.278	10.4	< 0.1	0.525	< 0.1
F1g B-34	0.148	0.257	4.47	0.861	10.7	< 0.1	0.979	0.201
F1g B-38	0.183	0.200	3.03	1.07	13.7	< 0.1	15.7	3.88
F1g B-39	0.119	0.227	2.08	0.727	13.0	< 0.1	4.24	0.266
F1g B-40	0.134	0.302	2.24	0.137	14.5	< 0.1	2.75	0.259
F1g B-41	0.300	1.19	2.63	0.987	34.6	< 0.1	9.22	0.255
F1g B-42	0.148	0.612	1.63	0.345	19.7	< 0.1	2.15	0.247
F1g B-43	< 0.1	0.33	0.957	0.170	9.4	< 0.1	0.667	< 0.1
F1g B-45	0.324	0.658	3.57	1.13	28.8	< 0.1	4.15	0.625
F1g B-46	0.212	0.663	2.3	0.421	23.2	< 0.1	1.91	< 0.1
F1g B-47	0.577	1.09	4.39	2.35	32.5	< 0.1	7.58	0.599
F1g B-48	0.202	0.838	2.25	0.416	25.5	< 0.1	2.17	< 0.1
F1g B-49	0.126	0.352	1.48	0.206	12.3	< 0.1	0.806	< 0.1
F1g B-50	< 0.1	0.270	1.2	< 0.1	17.2	< 0.1	0.388	< 0.1

Table S42. Outdoor irrigation results of the test specimen F 1g B: heavy metal and trace element concentrations, part 2.

Leachate	Cu µg/l	Mo µg/l	Ni µg/l	Hg µg/l	Se µg/l	Tl µg/l	V µg/l	Zn µg/l
F1g B-1	7.06	2.96	6.7	0.0146	2.49	< 0.1	42.7	5.17
F1g B-2	1.12	< 0.1	1.86	< 0.01	0.222	< 0.1	3.91	1.83
F1g B-3	1.32	< 0.1	1.14	< 0.01	0.132	< 0.1	3.18	1.58
F1g B-4	1.58	0.605	2.59	< 0.01	0.229	< 0.1	5.58	1.21
F1g B-5	1.84	0.675	1.66	< 0.01	0.275	< 0.1	4.82	2.54
F1g B-6	0.422	0.225	< 0.1	< 0.01	< 0.1	< 0.1	1.1	0.578
F1g B-7*	0.571	0.337	0.442	< 0.01	< 0.1	< 0.1	0.577	1.74
F1g B-8	1.36	0.289	< 0.1	< 0.01	< 0.1	< 0.1	1.23	4.41

F1g B-9	0.279	0.154	< 0.1	< 0.01	< 0.1	< 0.1	0.478	1.18
F1g B-10	1.19	0.157	< 0.1	< 0.01	0.216	< 0.1	1.1	2.74
F1g B-11	1.69	0.253	0.284	< 0.01	0.379	< 0.1	0.789	5.10
F1g B-12	0.569	0.234	0.204	< 0.01	0.198	< 0.1	0.463	2.52
F1g B-13	0.520	0.234	0.209	< 0.01	0.287	< 0.1	0.157	2.66
F1g B-14	0.374	< 0.1	< 0.1	< 0.01	< 0.1	< 0.1	< 0.1	1.46
F1g B-15	2.02	0.415	2.39	< 0.01	0.178	< 0.1	0.786	6.58
F1g B-16	0.363	0.130	1.51	< 0.01	< 0.1	< 0.1	0.383	2.31
F1g B-17	12.1	1.43	2.74	0.0248	0.787	< 0.1	3.68	31.9
F1g B-18	4.67	2.26	29.0	< 0.01	0.561	< 0.1	3.04	4.40
F1g B-19	1.86	0.406	2.12	< 0.01	0.106	< 0.1	1.18	3.15
F1g B-20	0.546	0.331	1.16	< 0.01	0.116	< 0.1	0.409	4.74
F1g B-21	< 0.1	0.135	0.444	< 0.01	< 0.1	< 0.1	0.600	< 1
F1g B-22	13.7	1.83	4.97	0.017	1.14	< 0.1	3.89	20.4
F1g B-23	2.93	0.513	2.14	< 0.01	0.194	< 0.1	1.79	4.7
F1g B-24	18.7	1.63	4.37	0.0101	1.04	< 0.1	5.69	21.8
F1g B-25	28.6	3.48	7.07	0.0168	1.06	< 0.1	14.1	23.1
F1g B-26	4.88	0.403	3.27	< 0.01	0.229	< 0.1	1.75	11.6
F1g B-27	1.68	0.349	2.05	< 0.01	< 0.1	< 0.1	1.38	8.37
F1g B-28	1.23	0.233	0.793	< 0.01	0.186	< 0.1	1.52	3.58
F1g B-29	0.848	0.224	2.28	< 0.01	< 0.1	< 0.1	1.18	4.68
F1g B-30	2.44	0.719	1.61	< 0.01	0.681	< 0.1	2.96	5.66
F1g B-31	1.14	0.412	2.19	< 0.01	0.169	< 0.1	1.07	4.47
F1g B-32	1.44	0.235	1.51	< 0.01	< 0.1	< 0.1	0.865	3.73
F1g B-33	1.56	0.333	1.09	< 0.01	0.127	< 0.1	1.78	3.5
F1g B-34	1.76	0.299	1.15	< 0.01	0.121	< 0.1	2.64	8.97
F1g B-38	12.8	**13.1**	41.9	< 0.01	0.234	< 0.1	1.82	11.6
F1g B-39	2.23	**15.6**	13.2	< 0.01	< 0.1	< 0.1	1.73	5.06
F1g B-40	1.11	**8.37**	7.32	< 0.01	0.238	< 0.1	2.58	1.24
F1g B-41	4.82	**6.61**	6.75	< 0.01	0.417	< 0.1	8.84	7.19
F1g B-42	1.46	**3.27**	4.23	< 0.01	0.188	< 0.1	5.05	4.52
F1g B-43	0.458	**1.62**	1.69	< 0.01	< 0.1	< 0.1	2.19	1.34
F1g B-45	3.73	**54.9**	5.36	< 0.01	0.354	< 0.1	4.38	8.46
F1g B-46	1.19	0.228	1.30	< 0.01	0.336	< 0.1	5.31	5.88
F1g B-47	5.16	0.490	6.06	< 0.01	1.01	< 0.1	5.61	22.7
F1g B-48	1.51	1.21	2.26	< 0.01	0.187	< 0.1	5.44	3.84
F1g B-49	0.733	0.685	1.32	< 0.01	0.132	< 0.1	2.44	1.19
F1g B-50	0.357	1.00	0.679	< 0.01	< 0.1	< 0.1	1.14	< 1

Bold: Significantly increased blank values for molybdenum, probably through contaminations during analysis or by particle deposits, led to likewise increased concentrations for the test specimens.

Table S43. Outdoor irrigation results of the test specimen F 4g A: leachate amount, pH value, electrical conductivity, and concentrations of sodium, potassium, calcium, chloride, and sulfate.

Date of Sampling	Leachate Label	Amount L/m²	pH -	EC µS/cm	Na mg/L	K mg/L	Ca mg/L	Cl^- mg/L	SO_4^{2-} mg/L
25.10.2018	F4g A-1	2.73	10.1	1219	107	258	1.9	4.1	31.8
01.11.2018	F4g A-2	14.4	10.1	236	20.5	57.0	1.9	0.6	3.8
08.11.2018	F4g A-3	1.87	9.62	286	37.6	68.4	2.1	0.3	2.5
15.11.2018	F4g A-4	6.52	10.0	310	27.4	69.0	1.5	1.3	3.9
29.11.2018	F4g A-5	6.38	9.67	266	30.6	70.9	1.9	0.3	7.4
06.12.2018	F4g A-6	25.3	7.5	77	7	16	3.0	0.4	0.4

13.12.2018	F4g A-7	26.1	7.1	80	8	13	3.5	2.3	< 0.1
20.12.2018	F4g A-8	5.71	7.5	107	13	21	2.4	0.5	0.6
27.12.2018	F4g A-9	27.6	7.4	61	5	11	3.6	0.5	0.6
03.01.2019	F4g A-10	11.0	7.2	115	12	22	3.8	0.3	< 0.1
10.01.2019	F4g A-11	21.2	7.2	91	9	13	4.6	1.7	0.4
17.01.2019	F4g A-12	32.8	7.3	60	5	8	4.4	1.0	0.3
24.01.2019	F4g A-13	2.16	7.7	79	7	10	5.3	2.7	1.1
31.01.2019	F4g A-14	21.7	6.8	30	5	< 0.1	1.3	4.7	0.5
07.02.2019	F4g A-15	6.34	7.0	223	36	20	3.8	3.8	2.3
14.02.2019	F4g A-16	25.3	8.0	63	6	8	4.2	2.1	0.8
21.02.2019	F4g A-17	0.23	7.6	675	7	118	6.6	9.0	37.3
28.02.2019	F4g A-18	0.56	7.7	310	7	53	4.8	3.0	12.6
07.03.2019	F4g A-19	21.7	7.1	107	6.5	14.8	3.0	1.2	1.4
14.03.2019	F4g A-20	47.1	7.7	70	3.8	6.3	3.4	2.9	0.9
21.03.2019	F4g A-21	33.9	7.5	45	2.1	3.9	4.9	1.1	0.2
28.03.2019	F4g A-22	0.36	6.5	405	52.7	65.9	5.5	117	245
04.04.2019	F4g A-23	5.31	7.2	79	7.3	13.1	3.4	1.9	3.2
11.04.2019	F4g A-24	0.57	6.9	328	34.7	61.1	4.2	4.2	25.2
18.04.2019	F4g A-25	0.34	6.9	551	64.3	110.5	5.2	101.0	0.2
25.04.2019	F4g A-26	5.85	6.9	65	3.6	9.0	3.9	0.2	1.5
02.05.2019	F4g A-27	8.17	7.2	87	7.2	14.6	3.3	2.5	1.0
09.05.2019	F4g A-28	14.8	7.5	115	9.8	20.8	2.4	1.7	1.1
16.05.2019	F4g A-29	7.88	7.4	87	6.3	16.5	2.4	0.7	0.4
23.05.2019	F4g A-30	7.98	7.7	131	13.5	33.2	2.6	1.6	2.9
30.05.2019	F4g A-31	12.1	7.2	63	4.5	12.8	3.0	2.4	1.4
06.06.2019	F4g A-32	23.5	7.0	39	1.7	4.8	3.6	0.3	1.2
13.06.2019	F4g A-33	9.33	7.1	66	5.4	11.5	3.2	0.6	2.0
20.06.2019	F4g A-34	8.84	7.3	68	4.9	11.0	4.9	0.4	2.2
18.07.2019	F4g A-38	8.22	8.3	50	3.0	6.1	4.6	0.7	2.4
25.07.2019	F4g A-39	7.30	n. d.	n. d.	2.9	5.9	3.4	2.5	0.4
01.08.2019	F4g A-40	19.3	n. d.	n. d.	3.4	8.2	6.1	0.4	1.4
08.08.2019	F4g A-41	3.11	7.3	135	19.2	30.0	2.2	4.1	4.8
15.08.2019	F4g A-42	12.1	7.3	74	11.1	22.5	2.3	1.8	1.8
22.08.2019	F4g A-43	17.9	7.2	51	4.9	12.4	2.5	0.5	0.7
05.09.2019	F4g A-45	3.57	7.2	96	9.4	20.2	4.4	2.5	3.5
12.09.2019	F4g A-46	8.00	7.5	118	11.3	21.2	2.9	2.6	4.0
19.09.2019	F4g A-47	1.23	6.9	170	16.0	33.7	2.7	20.2	7.9
26.09.2019	F4g A-48	7.42	7.4	119	10.0	21.8	2.4	0.6	1.9
03.10.2019	F4g A-49	25.3	7.4	47	3.4	7.9	2.4	0.4	0.9
10.10.2019	F4g A-50	33.7	7.4	47	3.9	8.2	3.6	0.8	0.5

Due to the absence of run-off, no samples were taken on the following dates: 18.10.2018. 22.11.2018. 27.06.2019. 04.07.2019. 11.07.2019. 29.08.2019; n. d. = not determined.

Table S44. Outdoor irrigation results of the test specimen F 4g A: heavy metal and trace element concentrations, part 1.

Leachate	Sb µg/l	As µg/l	Ba µg/l	Pb µg/l	B µg/l	Cd µg/l	Cr µg/l	Co µg/l
F4g A-1	1.30	5.58	3.63	1.10	106	< 0.1	8.63	0.236
F4g A-2	0.210	1.02	2.07	0.342	15.9	< 0.1	1.35	< 0.1
F4g A-3	0.295	1.85	3.60	0.326	27.1	< 0.1	2.21	0.148
F4g A-4	0.326	1.40	1.32	0.457	22.4	< 0.1	1.35	< 0.1
F4g A-5	0.36	1.92	1.89	0.757	25.3	< 0.1	1.58	0.143

F4g A-6	< 0.1	0.194	0.900	0.133	3.79	< 0.1	0.299	< 0.1
F4g A-7	< 0.1	0.233	0.862	0.193	4.08	< 0.1	0.381	< 0.1
F4g A-8	< 0.1	0.285	0.951	0.292	3.64	< 0.1	0.440	< 0.1
F4g A-9	< 0.1	0.133	0.718	0.115	2.00	< 0.1	0.203	< 0.1
F4g A-10	0.190	0.398	1.64	0.429	6.25	< 0.1	0.490	< 0.1
F4g A-11	0.218	0.297	1.90	0.479	5.05	< 0.1	0.387	< 0.1
F4g A-12	0.113	0.154	0.932	0.317	2.70	< 0.1	0.213	< 0.1
F4g A-13	< 0.1	0.114	1.13	0.179	3.69	< 0.1	0.203	< 0.1
F4g A-14	< 0.1	< 0.1	2.25	0.358	1.00	< 0.1	< 0.1	< 0.1
F4g A-15	0.162	0.295	1.55	0.575	3.25	< 0.1	0.519	< 0.1
F4g A-16	< 0.1	0.198	0.640	0.172	2.20	< 0.1	0.275	< 0.1
F4g A-17	0.816	4.25	5.98	2.25	35.4	0.21	5.37	0.715
F4g A-18	0.507	1.24	3.17	2.07	27.7	< 0.1	4.31	0.933
F4g A-19	0.170	0.298	1.33	0.613	5.59	< 0.1	0.477	< 0.1
F4g A-20	< 0.1	< 0.1	0.979	0.735	3.76	< 0.1	0.167	< 0.1
F4g A-21	< 0.1	< 0.1	0.796	0.129	1.99	< 0.1	0.109	< 0.1
F4g A-22	0.719	1.48	8.38	5.03	27.6	0.148	2.83	0.745
F4g A-23	0.177	0.412	2.24	1.18	8.37	< 0.1	1.97	0.335
F4g A-24	1.07	3.00	5.34	2.95	42.2	< 0.1	2.81	0.979
F4g A-25	1.26	5.03	13.0	4.85	65.8	0.114	6.62	1.28
F4g A-26	0.190	0.292	4.50	3.19	4.61	< 0.1	1.61	0.367
F4g A-27	0.170	0.368	1.42	0.396	9.88	< 0.1	1.06	0.114
F4g A-28	0.142	0.540	1.18	0.314	9.56	< 0.1	0.708	0.107
F4g A-29	0.129	0.358	1.42	0.505	7.74	< 0.1	0.696	< 0.1
F4g A-30	0.291	0.875	2.35	0.416	16.8	< 0.1	1.16	< 0.1
F4g A-31	< 0.1	0.311	1.05	0.272	6.84	< 0.1	0.494	0.14
F4g A-32	0.128	0.156	1.22	0.390	5.32	< 0.1	0.415	< 0.1
F4g A-33	0.189	0.376	1.63	0.472	12.1	< 0.1	0.645	< 0.1
F4g A-34	0.140	0.340	3.06	0.372	12.3	< 0.1	0.995	< 0.1
F4g A-38	0.199	0.321	3.98	1.31	17.6	< 0.1	1.21	0.273
F4g A-39	0.121	0.281	2.08	0.799	12.1	< 0.1	0.884	0.106
F4g A-40	0.131	0.307	1.86	0.265	13.6	< 0.1	1.07	< 0.1
F4g A-41	0.266	1.14	2.30	1.22	27.8	< 0.1	2.55	0.179
F4g A-42	0.192	0.818	1.72	0.771	20.2	< 0.1	1.35	< 0.1
F4g A-43	< 0.1	0.334	0.696	0.187	9.14	< 0.1	0.614	< 0.1
F4g A-45	0.329	0.733	4.06	1.77	27.0	< 0.1	1.90	0.322
F4g A-46	0.240	0.880	1.63	0.427	24.7	< 0.1	1.17	< 0.1
F4g A-47	0.492	1.34	2.51	0.988	32.5	< 0.1	1.76	0.231
F4g A-48	0.228	1.13	6.32	0.891	25.4	< 0.1	1.42	< 0.1
F4g A-49	< 0.1	0.299	0.642	0.108	8.49	< 0.1	0.515	< 0.1
F4g A-50	< 0.1	0.259	0.779	0.215	12.0	< 0.1	0.327	< 0.1

Table S45. Outdoor irrigation results of the test specimen F 4g A: heavy metal and trace element concentrations, part 2.

Leachate	Cu µg/l	Mo µg/l	Ni µg/l	Hg µg/l	Se µg/l	Tl µg/l	V µg/l	Zn µg/l
F4g A-1	5.12	4.84	3.08	< 0.01	2.79	< 0.1	49.3	9.29
F4g A-2	1.55	0.302	1.30	< 0.01	0.341	< 0.1	9.24	2.84
F4g A-3	1.95	0.990	1.50	< 0.01	0.584	< 0.1	16.5	1.62
F4g A-4	2.28	1.91	3.12	< 0.01	0.349	< 0.1	10.1	3.75
F4g A-5	4.54	1.22	2.61	< 0.01	0.574	< 0.1	14.1	5.94
F4g A-6	1.47	0.339	< 0.1	< 0.01	< 0.1	< 0.1	1.66	1.81

F4g A-7	0.684	0.317	< 0.1	< 0.01	0.140	< 0.1	1.74	1.70
F4g A-8	1.70	0.689	< 0.1	< 0.01	0.115	< 0.1	2.10	2.38
F4g A-9	0.598	0.194	< 0.1	< 0.01	< 0.1	< 0.1	1.02	1.26
F4g A-10	2.10	0.487	< 0.1	< 0.01	0.268	< 0.1	2.21	2.84
F4g A-11	2.45	0.480	< 0.1	< 0.01	0.436	< 0.1	1.42	3.11
F4g A-12	0.749	0.264	< 0.1	< 0.01	0.233	< 0.1	0.725	1.98
F4g A-13	0.681	0.278	< 0.1	< 0.01	0.307	< 0.1	0.400	2.48
F4g A-14	1.48	< 0.1	< 0.1	< 0.01	< 0.1	< 0.1	< 0.1	13.2
F4g A-15	2.18	0.663	1.45	< 0.01	0.187	< 0.1	1.39	5.60
F4g A-16	0.531	0.322	1.15	< 0.01	0.148	< 0.1	1.20	1.09
F4g A-17	23.5	4.95	6.46	0.0275	2.66	< 0.1	28.7	14.9
F4g A-18	8.24	3.21	66.2	< 0.01	0.795	< 0.1	8.16	11.6
F4g A-19	1.72	0.453	1.74	< 0.01	0.149	< 0.1	2.19	5.75
F4g A-20	0.648	0.272	0.316	< 0.01	< 0.1	< 0.1	0.546	3.14
F4g A-21	0.173	0.112	0.148	< 0.01	< 0.1	< 0.1	0.462	< 1
F4g A-22	24.7	2.83	7.85	0.017	1.67	< 0.1	8.06	63.5
F4g A-23	3.57	1.40	11.2	< 0.01	0.235	< 0.1	2.94	8.85
F4g A-24	22.0	4.29	30.7	0.0141	1.60	< 0.1	14.0	19.5
F4g A-25	27.5	4.30	26.7	< 0.01	1.37	< 0.1	33.0	46.1
F4g A-26	4.74	0.796	11.9	< 0.01	0.114	< 0.1	1.98	20.7
F4g A-27	1.41	0.97	9.28	< 0.01	0.131	< 0.1	2.68	4.45
F4g A-28	1.75	1.04	3.92	< 0.01	0.249	< 0.1	3.57	2.85
F4g A-29	1.32	0.543	1.24	< 0.01	0.182	< 0.1	2.12	6.16
F4g A-30	3.03	1.20	2.07	< 0.01	0.919	< 0.1	5.96	5.41
F4g A-31	1.61	0.547	1.74	< 0.01	0.194	< 0.1	1.66	4.76
F4g A-32	1.14	0.346	0.862	< 0.01	< 0.1	< 0.1	1.02	5.21
F4g A-33	1.69	0.739	0.916	< 0.01	0.140	< 0.1	2.90	4.58
F4g A-34	1.50	0.729	0.73	< 0.01	0.121	< 0.1	3.37	3.10
F4g A-38	4.19	2.02	11.5	< 0.01	0.279	< 0.1	2.41	16.9
F4g A-39	2.30	**10.5**	4.00	< 0.01	0.107	< 0.1	2.14	5.74
F4g A-40	1.28	**6.82**	4.47	< 0.01	0.224	< 0.1	2.72	4.06
F4g A-41	3.80	**4.36**	3.81	< 0.01	0.354	< 0.1	9.03	17.5
F4g A-42	1.71	**2.68**	1.56	< 0.01	0.215	< 0.1	7.14	6.96
F4g A-43	0.634	**1.46**	0.891	< 0.01	< 0.1	< 0.1	2.56	< 1
F4g A-45	4.46	**43.2**	9.00	< 0.01	0.340	< 0.1	5.30	15.4
F4g A-46	1.50	0.490	6.47	< 0.01	0.354	< 0.1	7.66	4.27
F4g A-47	3.69	1.20	2.50	< 0.01	1.12	< 0.1	7.54	8.39
F4g A-48	3.39	1.18	1.13	< 0.01	0.242	< 0.1	7.68	8.25
F4g A-49	0.500	0.512	1.27	< 0.01	0.125	< 0.1	1.98	< 1
F4g A-50	0.578	1.00	0.556	< 0.01	< 0.1	< 0.1	1.37	1.67

Bold: Significantly increased blank values for molybdenum. probably through contaminations during analysis or by particle deposits. led to likewise increased concentrations for the test specimens.

Table S46. Outdoor irrigation results of the test specimen F 4g B: leachate amount, pH value, electrical conductivity, and concentrations of sodium, potassium, calcium, chloride, and sulfate.

Date of Sampling	Leachate Label	Amount L/m²	pH -	EC µS/cm	Na mg/L	K mg/L	Ca mg/L	Cl^- mg/L	SO_4^{2-} mg/L
25.10.2018	F4g B-1	2.96	9.87	1046	92.3	224.5	2.0	4.9	27.9
01.11.2018	F4g B-2	14.1	9.63	195.9	17.8	49.0	2.2	0.7	3.1
08.11.2018	F4g B-3	2.21	9.14	267	29.4	59.0	2.6	0.4	5.9
15.11.2018	F4g B-4	6.43	8.49	260.8	23.8	63.1	1.8	1.4	3.9

29.11.2018	F4g B-5	6.56	9.47	245	26.6	64.7	2.7	0.3	6.9
06.12.2018	F4g B-6	25.3	7.2	71	6.0	14.0	3.0	0.8	< 0.1
13.12.2018	F4g B-7	23.1	7.2	72	7.0	11.0	3.5	2.3	0.3
20.12.2018	F4g B-8	5.85	7.4	99	12.0	19.0	2.6	0.7	1.4
27.12.2018	F4g B-9	26.9	7.2	55	4.0	9.0	3.7	1.1	0.5
03.01.2019	F4g B-10	11.3	7.1	101	11.0	19.0	4.0	1.4	2.5
10.01.2019	F4g B-11	20.4	7.1	86	9.0	12.0	4.8	1.4	1.1
17.01.2019	F4g B-12	31.8	7.2	56	4.0	7.0	4.4	0.9	0.4
24.01.2019	F4g B-13	2.33	7.1	76	7.0	9.0	5.2	2.7	1.1
31.01.2019	F4g B-14	21.7	7	24	2.0	1.0	2.3	2.0	0.7
07.02.2019	F4g B-15	6.87	7.5	111	5.0	16.0	3.9	8.3	1.4
14.02.2019	F4g B-16	25.4	8.5	55	5.0	6.0	4.1	2.0	< 0.1
21.02.2019	F4g B-17	0.35	6.7	303	5.0	45.0	4.1	3.0	11.6
28.02.2019	F4g B-18	0.69	7.7	287	5.0	50.0	4.1	3.0	10.0
07.03.2019	F4g B-19	19.8	7.1	84	5.7	12.7	3.1	1.0	0.9
14.03.2019	F4g B-20	45.1	7.7	69	3.6	5.7	3.4	2.9	0.9
21.03.2019	F4g B-21	38.4	7.4	50	2.6	5.0	5.0	1.3	0.4
28.03.2019	F4g B-22	0.63	7.2	321	49.0	60.7	4.7	47.4	24.6
04.04.2019	F4g B-23	6.31	7.2	78	6.4	11.4	3.2	0.5	3.6
11.04.2019	F4g B-24	0.77	6.8	256	26.8	48.8	4.1	7.3	19.5
18.04.2019	F4g B-25	0.43	7.2	469	54.1	78.7	4.4	33.5	18.2
25.04.2019	F4g B-26	5.86	6.9	60	2.9	7.0	3.9	0.3	1.0
02.05.2019	F4g B-27	7.82	7.2	101	7.2	14.1	3.3	3.1	1.8
09.05.2019	F4g B-28	13.7	7.5	117	9.9	21.0	2.6	0.7	1.0
16.05.2019	F4g B-29	7.55	7.4	72	6.1	15.8	2.4	0.8	0.8
23.05.2019	F4g B-30	7.83	7.6	109	13.1	32.0	2.7	1.9	3.1
30.05.2019	F4g B-31	11.2	7.3	50	4.4	12.1	3.0	0.6	1.4
06.06.2019	F4g B-32	22.3	7.1	33	1.7	4.7	3.5	< 0.1	0.8
13.06.2019	F4g B-33	9.09	7	62	5.3	10.9	3.4	0.4	1.5
20.06.2019	F4g B-34	7.99	7.3	64	5.1	11.3	5.2	0.4	3.3
18.07.2019	F4g B-38	7.81	8.3	53	2.5	5.2	4.6	1.1	2.1
25.07.2019	F4g B-39	6.87	n. d.	n. d.	2.8	5.6	4.0	4.9	2.1
01.08.2019	F4g B-40	19.2	n. b	n. d.	3.1	7.5	6.3	0.3	0.8
08.08.2019	F4g B-41	2.80	7.3	157	19.7	30.7	2.3	5.4	8.0
15.08.2019	F4g B-42	11.9	7.3	95	10.9	21.2	2.3	1.8	2.8
22.08.2019	F4g B-43	17.5	7.3	63	5.0	12.5	2.5	0.4	0.4
05.09.2019	F4g B-45	3.39	7.3	110	9.5	20.1	5.6	3.4	4.1
12.09.2019	F4g B-46	7.91	7.4	107	10.7	19.9	2.8	3.1	2.7
19.09.2019	F4g B-47	1.19	6.7	191	15.4	32.3	3.0	48.9	7.6
26.09.2019	F4g B-48	7.81	7.4	91	11.2	24.9	2.4	0.9	2.6
03.10.2019	F4g B-49	25.4	7.3	44	3.8	9.1	2.4	0.9	0.5
10.10.2019	F4g B-50	33.2	7.5	44	3.7	7.7	3.5	0.8	0.3

Due to the absence of run-off, no samples were taken on the following dates: 18.10.2018. 22.11.2018. 27.06.2019. 04.07.2019. 11.07.2019. 29.08.2019; n. d. = not determined.

Table S47. Outdoor irrigation results of the test specimen F 4g B: heavy metal and trace element concentrations, part 1.

Leachate	Sb µg/l	As µg/l	Ba µg/l	Pb µg/l	B µg/l	Cd µg/l	Cr µg/l	Co µg/l
F4g B-1	1.20	4.65	4.83	1.74	108	< 0.1	8.33	0.34
F4g B-2	0.180	0.993	1.54	0.250	14.7	< 0.1	1.34	< 0.1
F4g B-3	0.271	1.81	3.45	0.427	22.8	< 0.1	1.95	0.144

F4g B-4	0.195	1.30	0.856	0.133	21.5	< 0.1	1.10	< 0.1
F4g B-5	0.36	1.85	2.85	1.28	23.5	< 0.1	1.47	0.136
F4g B-6	< 0.1	0.185	0.588	< 0.1	3.56	< 0.1	0.268	< 0.1
F4g B-7	< 0.1	0.160	0.816	0.383	3.53	< 0.1	0.310	< 0.1
F4g B-8	< 0.1	0.253	1.69	0.509	3.03	< 0.1	0.422	< 0.1
F4g B-9	< 0.1	0.131	0.629	< 0.1	1.50	< 0.1	0.165	< 0.1
F4g B-10	0.179	0.328	2.26	0.426	4.52	< 0.1	0.371	< 0.1
F4g B-11	0.201	0.273	1.95	0.649	4.70	< 0.1	0.242	< 0.1
F4g B-12	0.104	0.119	0.949	0.449	2.10	< 0.1	0.150	< 0.1
F4g B-13	0.117	< 0.1	1.80	0.746	3.74	< 0.1	0.208	< 0.1
F4g B-14	< 0.1	< 0.1	4.05	0.248	2.34	< 0.1	0.135	0.137
F4g B-15	0.149	0.236	1.99	1.21	2.88	< 0.1	0.407	0.103
F4g B-16	< 0.1	0.151	0.767	0.398	1.86	< 0.1	0.208	< 0.1
F4g B-17	0.566	0.821	6.11	2.43	13.7	< 0.1	2.21	0.355
F4g B-18	0.463	1.28	3.19	1.17	26.5	< 0.1	2.17	0.949
F4g B-19	0.148	0.243	1.21	0.444	4.66	< 0.1	0.317	< 0.1
F4g B-20	< 0.1	< 0.1	0.941	0.623	3.25	< 0.1	0.139	< 0.1
F4g B-21	< 0.1	< 0.1	0.773	0.175	3.92	< 0.1	0.166	< 0.1
F4g B-22	0.749	1.63	8.59	4.29	29.2	0.131	2.54	0.748
F4g B-23	0.177	0.424	2.53	1.13	6.70	< 0.1	0.606	0.143
F4g B-24	0.903	2.71	5.85	2.59	36.8	< 0.1	1.77	0.379
F4g B-25	0.922	4.28	8.22	2.77	53.6	< 0.1	3.25	0.591
F4g B-26	0.199	0.278	5.68	3.02	3.60	< 0.1	2.25	0.52
F4g B-27	0.166	0.382	1.41	0.488	9.45	< 0.1	1.06	< 0.1
F4g B-28	0.182	0.642	1.75	0.706	11.1	< 0.1	0.640	< 0.1
F4g B-29	0.130	0.367	1.56	0.531	7.68	< 0.1	0.562	< 0.1
F4g B-30	0.330	0.888	2.72	0.630	18.7	< 0.1	0.900	< 0.1
F4g B-31	0.115	0.302	1.23	0.541	9.31	< 0.1	0.460	< 0.1
F4g B-32	0.121	0.147	1.14	0.286	4.57	< 0.1	0.301	< 0.1
F4g B-33	0.138	0.412	1.31	0.407	12.0	< 0.1	0.638	< 0.1
F4g B-34	0.146	0.395	3.05	0.144	13.1	< 0.1	0.690	< 0.1
F4g B-38	0.185	0.288	3.62	1.04	14.5	< 0.1	3.54	1.37
F4g B-39	0.135	0.291	2.69	0.910	12.4	< 0.1	1.30	0.108
F4g B-40	0.135	0.295	1.73	0.158	13.7	< 0.1	1.68	< 0.1
F4g B-41	0.269	1.27	2.53	0.962	30.2	< 0.1	7.90	0.157
F4g B-42	0.160	0.877	1.11	0.341	20.6	< 0.1	1.73	0.141
F4g B-43	< 0.1	0.378	0.743	0.277	10.4	< 0.1	0.687	< 0.1
F4g B-45	0.352	0.826	5.03	2.76	26.8	0.132	4.57	1.12
F4g B-46	0.224	0.910	1.61	0.682	25.3	< 0.1	1.88	< 0.1
F4g B-47	0.600	1.42	2.97	1.82	33.5	< 0.1	3.72	0.764
F4g B-48	0.191	1.10	1.59	0.497	23.3	< 0.1	2.07	< 0.1
F4g B-49	< 0.1	0.271	0.584	0.111	8.25	< 0.1	0.426	< 0.1
F4g B-50	< 0.1	0.261	0.731	0.171	12.0	< 0.1	0.290	< 0.1

Table S48. Outdoor irrigation results of the test specimen F 4g B: heavy metal and trace element concentrations, part 2.

Leachate	Cu µg/l	Mo µg/l	Ni µg/l	Hg µg/l	Se µg/l	Tl µg/l	V µg/l	Zn µg/l
F4g B-1	4.27	5.00	4.06	0.0108	2.62	< 0.1	46.4	10.7
F4g B-2	0.988	< 0.1	1.22	< 0.01	0.343	< 0.1	8.89	1.86
F4g B-3	2.36	0.728	1.42	< 0.01	0.541	< 0.1	13.6	3.10
F4g B-4	1.37	1.55	2.73	< 0.01	0.423	< 0.1	8.89	1.34

F4g B-5	3.48	1.49	2.27	< 0.01	0.547	< 0.1	12.6	8.59
F4g B-6	0.529	0.266	< 0.1	< 0.01	0.120	< 0.1	1.58	0.833
F4g B-7	0.652	0.288	< 0.1	< 0.01	0.127	< 0.1	1.22	1.77
F4g B-8	2.08	0.520	< 0.1	< 0.01	< 0.1	< 0.1	1.71	3.03
F4g B-9	0.396	0.175	< 0.1	< 0.01	< 0.1	< 0.1	0.736	1.33
F4g B-10	4.10	0.317	< 0.1	< 0.01	0.341	< 0.1	1.78	2.54
F4g B-11	1.32	0.350	< 0.1	< 0.01	0.445	< 0.1	1.11	3.64
F4g B-12	0.797	0.185	< 0.1	< 0.01	0.251	< 0.1	0.514	2.68
F4g B-13	1.70	0.270	< 0.1	< 0.01	0.293	< 0.1	0.284	5.52
F4g B-14	3.45	0.189	< 0.1	< 0.01	< 0.1	< 0.1	0.103	16.4
F4g B-15	2.55	0.488	1.55	< 0.01	0.184	< 0.1	1.03	6.87
F4g B-16	0.645	0.182	0.991	< 0.01	0.184	< 0.1	0.825	2.77
F4g B-17	10.6	1.60	2.68	0.0142	0.693	< 0.1	5.75	27.0
F4g B-18	5.27	3.18	48.5	< 0.01	0.776	< 0.1	9.18	9.09
F4g B-19	1.06	0.422	1.06	< 0.01	0.171	< 0.1	1.67	3.30
F4g B-20	0.696	0.214	0.278	< 0.01	0.108	< 0.1	0.422	2.76
F4g B-21	0.184	0.281	0.210	< 0.01	< 0.1	< 0.1	0.973	1.29
F4g B-22	19.4	2.51	4.61	0.0147	1.69	< 0.1	9.51	41.7
F4g B-23	3.36	0.539	0.868	< 0.01	0.215	< 0.1	3.01	8.87
F4g B-24	15.6	1.93	2.74	0.0119	1.40	< 0.1	12.0	16.8
F4g B-25	18.6	2.85	3.93	< 0.01	1.32	< 0.1	29.0	25.0
F4g B-26	6.06	2.66	19.0	< 0.01	< 0.1	< 0.1	1.68	20.9
F4g B-27	1.22	0.777	2.16	< 0.01	0.175	< 0.1	2.81	3.78
F4g B-28	2.05	0.614	0.711	< 0.01	0.280	< 0.1	4.08	6.11
F4g B-29	1.26	0.458	0.366	< 0.01	0.157	< 0.1	2.15	6.10
F4g B-30	3.24	1.00	0.555	< 0.01	0.998	< 0.1	6.19	6.82
F4g B-31	1.34	0.641	0.409	< 0.01	0.172	< 0.1	1.82	5.77
F4g B-32	0.921	0.271	0.338	< 0.01	< 0.1	< 0.1	1.01	3.84
F4g B-33	1.76	0.562	0.545	< 0.01	0.136	< 0.1	3.02	4.44
F4g B-34	1.06	0.466	0.474	< 0.01	0.155	< 0.1	3.40	0.789
F4g B-38	5.18	**3.74**	80.1	< 0.01	0.229	< 0.1	2.14	15.7
F4g B-39	3.82	**10.9**	3.75	< 0.01	0.119	< 0.1	2.40	10.2
F4g B-40	0.984	**6.51**	5.54	< 0.01	0.208	< 0.1	2.91	3.38
F4g B-41	4.43	**5.48**	3.35	< 0.01	0.338	< 0.1	10.1	***90***
F4g B-42	1.14	**3.07**	1.86	< 0.01	0.204	< 0.1	8.01	2.24
F4g B-43	0.801	**1.59**	0.796	< 0.01	0.109	< 0.1	2.89	2.59
F4g B-45	5.14	**36.8**	45.4	< 0.01	0.414	< 0.1	5.95	18.0
F4g B-46	1.40	1.11	8.70	< 0.01	0.382	< 0.1	8.45	7.5
F4g B-47	4.08	1.13	8.14	< 0.01	1.12	< 0.1	8.22	16.0
F4g B-48	1.53	1.65	2.09	< 0.01	0.209	< 0.1	7.86	3.51
F4g B-49	0.443	0.631	0.946	< 0.01	0.116	< 0.1	1.97	< 1
F4g B-50	0.500	1.00	0.646	< 0.01	< 0.1	< 0.1	1.38	< 1

Bold: Significantly increased blank values for molybdenum, probably through contaminations during analysis or by particle deposits. led to likewise increased concentrations for the test specimens. ***Bold and italic***: Outlier due to contamination or analytical error.
